# Post-translational Lysine Ac(et)ylation in Bacteria: A Biochemical, Structural, and Synthetic Biological Perspective

**DOI:** 10.3389/fmicb.2021.757179

**Published:** 2021-10-11

**Authors:** Michael Lammers

**Affiliations:** Synthetic and Structural Biochemistry, Institute for Biochemistry, University of Greifswald, Greifswald, Germany

**Keywords:** lysine acetylation, lysine deacetylases, lysine acetyltransferases, genetic code expansion, sirtuin

## Abstract

Ac(et)ylation is a post-translational modification present in all domains of life. First identified in mammals in histones to regulate RNA synthesis, today it is known that is regulates fundamental cellular processes also in bacteria: transcription, translation, metabolism, cell motility. Ac(et)ylation can occur at the ε-amino group of lysine side chains or at the α-amino group of a protein. Furthermore small molecules such as polyamines and antibiotics can be acetylated and deacetylated enzymatically at amino groups. While much research focused on N-(ε)-ac(et)ylation of lysine side chains, much less is known about the occurrence, the regulation and the physiological roles on N-(α)-ac(et)ylation of protein amino termini in bacteria. Lysine ac(et)ylation was shown to affect protein function by various mechanisms ranging from quenching of the positive charge, increasing the lysine side chains’ size affecting the protein surface complementarity, increasing the hydrophobicity and by interfering with other post-translational modifications. While N-(ε)-lysine ac(et)ylation was shown to be reversible, dynamically regulated by lysine acetyltransferases and lysine deacetylases, for N-(α)-ac(et)ylation only N-terminal acetyltransferases were identified and so far no deacetylases were discovered neither in bacteria nor in mammals. To this end, N-terminal ac(et)ylation is regarded as being irreversible. Besides enzymatic ac(et)ylation, recent data showed that ac(et)ylation of lysine side chains and of the proteins N-termini can also occur non-enzymatically by the high-energy molecules acetyl-coenzyme A and acetyl-phosphate. Acetyl-phosphate is supposed to be the key molecule that drives non-enzymatic ac(et)ylation in bacteria. Non-enzymatic ac(et)ylation can occur site-specifically with both, the protein primary sequence and the three dimensional structure affecting its efficiency. Ac(et)ylation is tightly controlled by the cellular metabolic state as acetyltransferases use ac(et)yl-CoA as donor molecule for the ac(et)ylation and sirtuin deacetylases use NAD^+^ as co-substrate for the deac(et)ylation. Moreover, the accumulation of ac(et)yl-CoA and acetyl-phosphate is dependent on the cellular metabolic state. This constitutes a feedback control mechanism as activities of many metabolic enzymes were shown to be regulated by lysine ac(et)ylation. Our knowledge on lysine ac(et)ylation significantly increased in the last decade predominantly due to the huge methodological advances that were made in fields such as mass-spectrometry, structural biology and synthetic biology. This also includes the identification of additional acylations occurring on lysine side chains with supposedly different regulatory potential. This review highlights recent advances in the research field. Our knowledge on enzymatic regulation of lysine ac(et)ylation will be summarized with a special focus on structural and mechanistic characterization of the enzymes, the mechanisms underlying non-enzymatic/chemical ac(et)ylation are explained, recent technological progress in the field are presented and selected examples highlighting the important physiological roles of lysine ac(et)ylation are summarized.

## Introduction

While humans contain a genome size of 6.2 Mbp in the diploid state, in which only 3% encode for approximately 20,000 proteins, bacteria contain genome sizes ranging from <0.5 to 10 Mbp (Ponomarenko et al., 2016; Willyard, 2018; Piovesan et al., 2019). Free-living bacteria such as *Escherichia*, *Bacillus*, and *Salmonella* species encode 1,500–7,500 proteins (Fredens et al., 2019). Assuming the rather low number of protein encoding genes in bacteria raises the question how the complex cellular functions can be exerted with such a small protein repertoire. Although the number of proteins is limited, their functional diversity can be enlarged significantly by post-translational modifications (Cain et al., 2014; Macek et al., 2019). These modifications can occur co-translationally during protein synthesis at the ribosome or post-translationally following translation and protein folding is completed. In eukaryotes, the acetylation of the protein amino (N)-terminus, N-terminal acetylation, can occur co-translationally and post-translationally (Drazic et al., 2016). In bacteria the N-terminal N-formyl-methionine is removed by methionine aminopeptidase and N-terminal acetylation is post-translational at least for the characterized proteins in bacteria (Gordiyenko et al., 2008; Roy-Chaudhuri et al., 2008; Drazic et al., 2016; Christensen et al., 2019b). However, co-translational acetylation might also occur in bacteria. Post-translational modifications in bacteria are very diverse and they can modify protein structure and function (Macek et al., 2019). Half of all 20 proteinogenic amino acids can be modified following translation. This includes the attachment of chemical groups such as phosphorylation, methylation, lipidation, ac(et)ylation or other acylations and prenylations. Moreover, proteins can be modified in bacteria by addition of another protein such as the prokaryotic ubiquitin-like protein (Pup), or by attachment of sugar moieties called glycosylation (Macek et al., 2019). Similar as observed in eukaryotes, PTMs can be dynamic and reversible such as phosphorylation which are attached by kinases and which can be removed by phosphatases. Another important dynamic and reversible PTM is the ac(et)ylation of the epsilon (ε)-amino group of lysine side chains. These dynamic PTMs allows cells to react with a fast energy saving response to altered conditions without the need to degrade or synthesize novel proteins. Lysine acetylation was identified in mammals already in the 1960s to occur on histones (Phillips, 1963; Allfrey and Mirsky, 1964; Allfrey et al., 1964). The discovery that the sirtuin deacetylase SIR2 in *Saccharomyces cerevisiae* affects its replicative lifespan was remarkable and showed that the PTM is an important cellular regulatory PTM (Kaeberlein et al., 1999; Imai et al., 2000). While the study of lysine ac(et)ylation, the enzymes involved in its regulation, and the consequences of lysine ac(et)ylation on protein function was studied thoroughly in eukaryotes, its investigation lacked behind in bacteria. Notably, almost 35 years after the discovery of lysine acetylation in mammalian histones, the enzyme ac(et)yl-CoA-synthetase (Acs) and the protein CheY involved in chemotaxis were the first bacterial proteins shown to be regulated by lysine ac(et)ylation (Barak and Eisenbach, 2001; Starai et al., 2002). Today, it is known that up to 40% of all bacterial proteins are lysine acetylated and that lysine ac(et)ylation plays important roles in regulation of transcription, translation, metabolism, stress response, chemotaxis, and virulence (Barak et al., 2006; Yan et al., 2008; Castano-Cerezo et al., 2014; Meng et al., 2016; Ren et al., 2016, 2019; Sang et al., 2016; Zhang et al., 2020; Li et al., 2021). Lysine ac(et)ylation was shown to be regulated in bacteria enzymatically by the action of lysine acetyltransferases (KATs) and lysine deacetylases, e.g., NAD^+^-dependent sirtuins (SIRT; silent information regulator) and classical Zn^2+^-dependent lysine deacetylases (KDACs) (VanDrisse and Escalante-Semerena, 2019). Pathogenic Gram-negative bacterial species such as *Legionella*, *Salmonella*, *Rickettsia*, *Chlamydia*, *Vibrio*, and *Yersinia* inject virulence factors into host cells that have a dual deubiquitinase and/or acetyltransferase activity supporting an efficient infection process (Viboud and Bliska, 2005; Mittal et al., 2006; Ma and Ma, 2016; Pruneda et al., 2016, 2018; Hermanns and Hofmann, 2019; Hermanns et al., 2020). These enzymes are CE-clan protease-related enzymes, and future studies are needed to unravel the physiological roles of these enzymatic activities during the infection process (Pruneda et al., 2016, 2018; Hermanns and Hofmann, 2019; Hermanns et al., 2020). Moreover, non-enzymatic ac(et)ylation was described to occur both in eukaryotes and in prokaryotes. However, while the thioester and central metabolism molecule ac(et)yl-CoA drives most non-enzymatic ac(et)ylation in eukaryotes, the high-energy molecule acetyl-phosphate is reported to mediate most non-enzymatic ac(et)ylation in bacteria (Verdin and Ott, 2013; Weinert et al., 2013a; Ren et al., 2019). Recent mass spectrometric data performed with bacterial cells revealed that besides from acetylation, other acylations, such as aliphatic butyrylation and propionylation or negatively charged succinylation, can also occur at lysine side chains (Colak et al., 2013; Weinert et al., 2013b; Xu et al., 2018a, b; Christensen et al., 2019b; Zhao et al., 2020). Future studies will reveal to which extent further lysine acylations exist in bacteria, how they are regulated, and how they exert mechanistically different effects to regulate protein function. Another important technological progress in studying the role of lysine acetylation to regulate protein function was the development of a system that allows to genetically encode acetyl-L-lysine in proteins (genetic code expansion concept; GCEC) (Neumann et al., 2008, 2009; Lammers et al., 2010). This system applies a synthetically evolved acetyl-lysyl-tRNA_CUA_ (AcKRS3)//tRNA_CUA_ (PylT) pair based on the pyrrolysyl-tRNA-synthetase (PylS)/PylT pair from archaea of the genera *Methanosarcina*.

This review summarizes findings on regulation of lysine ac(et)ylation by enzymatic and non-enzymatic mechanisms and it reports novel technological progress that allows to deepen our understanding on lysine ac(et)ylation including mass spectrometric workflows to determine stoichiometry of lysine ac(et)ylation at a systemic resolution. This information is essential in order to judge the physiological significance of a specific lysine ac(et)ylation PTM. Furthermore, we describe recent developments in synthetic biological approaches including genetic code expansion to unravel the real consequences of lysine ac(et)ylation for protein function rather than performing mutational approaches which are sometimes misleading. This review also reports the current knowledge on novel CE-clan protease related bacterial pathogenicity factors with dual deubiquitinase and/or acetyltransferase activities (Pruneda et al., 2016, 2018; Hermanns and Hofmann, 2019; Hermanns et al., 2020). Importantly, these enzymes use the same active site for catalysis of both activities and these enzymes catalyze also ac(et)ylation of Ser and Thr residues next to Lys. We will set emphasize on structure function analyses of the enzymes involved. Finally, a summary of knowledge of physiologically important roles of lysine ac(et)ylations describing selected examples is presented before closing with giving a perspective for future research directions to further characterize this important PTM for bacterial physiology.

## Ac(Et)Ylation Is Regulated Enzymatically and Non-Enzymatically

**FIGURE 1:**
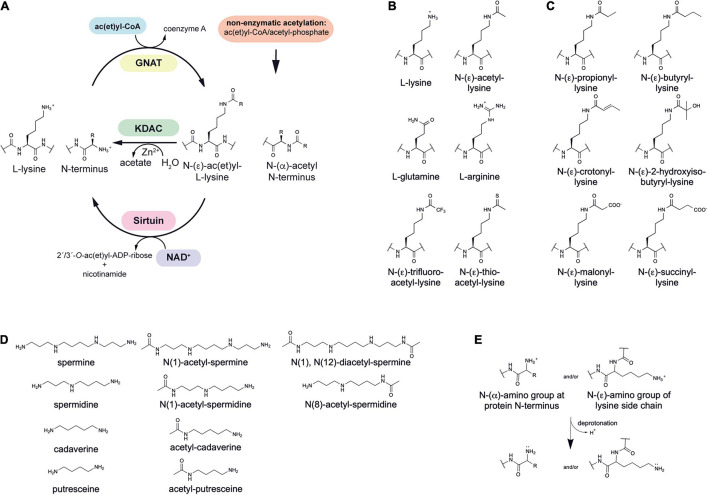
Post-translational lysine ac(et)ylation is dynamically regulated by lysine deac(et)ylases and N-(α)-/N-(ε)-ac(et)yltransferases. **(A)** The ε-amino group of lysine side chains and the α-amino group at the protein N-termini can be ac(et)ylated by lysine acetyltransferases (KATs) using acetyl-CoA and/or further acyl-CoA donor molecules for the ac(et)ylation. So far, all bacterial protein acetyltransferases belong to the Gcn5-related N-terminal acetyltransferases (GNAT). Next to the enzymatic ac(et)ylation, lysine side chains and protein N-termini can ac(et)ylated non-enzymatically by ac(et)yl-CoA and acetyl-phosphate, the major source for non-enzymatic acetylation in bacteria. Bacteria use Zn^2+^-dependent classical lysine deac(et)ylases and NAD^+^-dependent sirtuin deac(et)ylases to catalyze the deac(et)ylation of lysine side chains. **(B)** Structures of amino acids used to study lysine acetylation. L-glutamine is often used to mimic lysine acetylation and L-arginine to conserve a non-acetylated state in studies performed *in vivo*. Trifluoroacetyl-L-lysine and thioacetyl-L-lysine are used as mechanistic inhibitors for sirtuins to stabilize the acetylation at an analyzed site. Notably, these analogs can be potently deacetylated by some classical deacetylases. **(C)** Diverse acylations identified to occur at lysine side chains in bacteria. Lysine side chains can be modified by various aliphatic or negatively charged acylations in bacteria. Further acylations might be discovered in future. To which extend acetyltransferases are capable to catalyze acylation of the lysine side chains and/or protein N-termini needs further investigation. In general, all acyl-CoA thioesters generated in metabolism can be transferred to the ε-amino group of lysine side chains and/or the α-amino group at the protein N-termini in terms of an non-enzymatic reaction. **(D)** Polyamines in bacteria shown to be acetylated by acetyltransferases and deacetylated by classical deacetylases. These polyamines might form buffers for acetyl-groups to avoid systemic non-enzymatic protein acetylation. The acetyl-groups can be transferred from acetyl-CoA by the action of polyamine specific acetyltransferases. **(E)** Increasing the reactivity at the N-(ε)-amino group of lysine side chains or the N-(α)-amino group of the protein N-termini for enzymatic or non-enzymatic ac(et)ylation. Deprotonation of the α- or ε-amino groups by protein acetyltransferases in an important step for acetyl-group transfer from the ac(et)yl-CoA donor molecule during catalysis. A deprotonation can also occur non-enzymatically and is supported by the presence of the lysine side chain in a poly-basic sequence context resulting in the reduction of the substrate lysine side chain’s pK_a_ value. Moreover, non-enzymatic ac(et)ylation is preferred under alkaline conditions and under high concentrations of the reactive ac(et)yl-CoA thioesters. A deprotonation of the substrate amino group results in an increase in its nucleophilicity for attack of the ac(et)yl-CoA thioesters.

Lysine ac(et)ylation is a PTM that is catalyzed either enzymatically by the actions of KATs or SIRT/KDACs, or non-enzymatically by the appearance and accumulation of ac(et)yl-CoA and/or acetyl-phosphate (Figure 1A). Next to acetylation many further acylations were discovered to occur at lysine side chains and/or on at protein N-termini (Figures 1B,C). Moreover, acetyltransferases and deacetylases for acetylated polyamines are reported (Figure 1D). While the enzymatic regulation of lysine ac(et)ylation allows the cells to dynamically accumulate or remove ac(et)yl groups from lysine side chains, i.e., they control the presence and stoichiometry of a specific lysine ac(et)ylation in a protein, non-enzymatic ac(et)ylation can be a unwanted side product on ac(et)yl-CoA or acetyl-phosphate accumulation dependent on the cellular metabolic state. However, also site-specific non-enzymatic ac(et)ylation was reported which depends on the primary sequence and on the three dimensional structure of the target protein. Basis for enzymatic and non-enzymatic ac(et)ylation is that the reactivity of the substrate amino group is enhanced by deprotonation (Figure 1E). The following sections describes the current state of knowledge on enzymatic and non-enzymatic ac(et)ylation in bacteria.

## Lysine Acetyltransferases in Bacteria

In mammals three families of lysine acetyltransferases can be distinguished based on sequence and structure: P300/CBP (p300/CREB-binding proteins), MYST (Moz, Ybf2, Sas2, and Tip60) and GNAT (Gcn5-related N-terminal acetyltransferases) (Friedmann and Marmorstein, 2013; McCullough and Marmorstein, 2016). Additional KATs were reported which cannot be categorized in any of these families based on sequence and structure. Members of the P300/CBP family exert a Theorell-Chance hit-and-run mechanism for catalysis. This catalytic strategy involves an active site tyrosine residue that orients the substrate lysine side chain increasing its nucleophilicity (Zhang et al., 2014; Ali et al., 2018; Blasl et al., 2021). The lysine is able to attack the ac(et)yl-CoA carbonyl carbon while the tyrosine residue acts as catalytic acid protonating the sulfhydryl group of the ac(et)yl-CoA finally resulting in formation of acetyl-lysine and coenzyme A. In contrast, members of the MYST and GNAT families use a catalytic glutamate acting as catalytic base for catalysis (Friedmann and Marmorstein, 2013; McCullough and Marmorstein, 2016; Ali et al., 2018; Blasl et al., 2021). This glutamate abstracts a proton from the lysine side chain increasing its nucleophilicity for attack of the carbonyl carbon of the ac(et)yl-CoA. A tetrahedral intermediate is formed, which subsequently is resolved to form acetyl-lysine and coenzyme A.

The first GNAT enzyme was identified in multi-drug resistant *E. coli* already in 1965 and showed an activity as aminoglycoside acetyltransferase conferring resistance toward chloramphenicol and kanamycin (Okamoto and Suzuki, 1965). The first lysine acetyltransferase (KAT) identified in bacteria was Pat (protein acetyl transferase) in *Salmonella enterica* (Starai and Escalante-Semerena, 2004b; Thao and Escalante-Semerena, 2011; Crosby et al., 2012; VanDrisse and Escalante-Semerena, 2019). Later studies showed that many bacterial species including *Escherichia coli*, *Bacillus subtilis*, *Rhodopseudomonas palustris*, *Mycobacterium tuberculosis*, encode a Pat homolog (Wolfe, 2016; VanDrisse and Escalante-Semerena, 2019).

**FIGURE 2:**
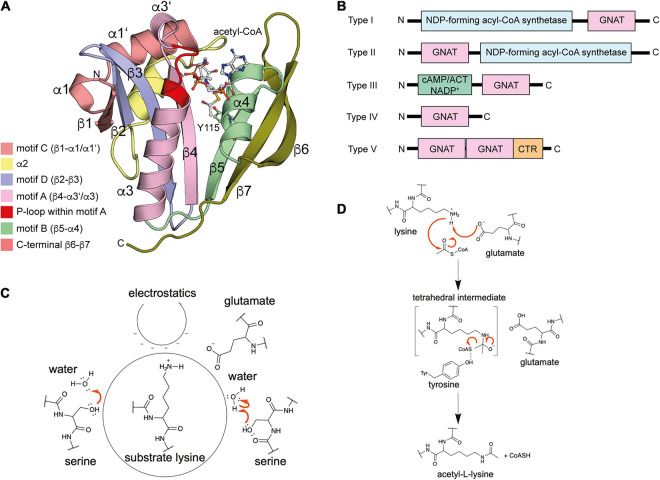
Enzymatic protein ac(et)ylation is catalyzed by GNAT ac(et)yltransferases in bacteria. **(A)** Structure of the *E. coli* GNAT acetyltransferase RimI in complex with acetyl-CoA (PDB: 2CNS). All bacterial protein acetyltransferases belong to the GNATs. These are characterized by sequence motifs A-D as indicated. Motifs A and B are important for CoA-binding. Motif A contains the characteristic sequence motif Arg/Gln-x-x-Gly-x-Gly/Ala (x: any amino acid), known as P-loop, which contacts the phosphates of the acetyl-CoA/CoA. The acetyl-CoA is shown in ball-and-stick representation [the figure was generated with PyMOL v.2.3.4 (Schrödinger LLC, 2000)]. **(B)** Domain organization of different bacterial GNAT types. Type I GNATs contain an N-terminal and type II GNATs a C-terminal NDP-forming acyl-CoA synthetase domain. These domains are catalytically inactive, but they bind acetyl-CoA and are important for allosteric regulation of GNAT activity. Type III GNATs encompass an N-terminal ligand binding domain such as a cAMP-binding domain with high similarity for cAMP-binding domains of EPAC, PKA, CAP/CRP, a Rossmann-fold domain specific for NADP^+^-binding, or an ACT domain for binding to amino acids cysteine, arginine and/or asparagine. Binding of these metabolic molecules to the N-terminal domain activates the C-terminal GNAT activity. Type IV GNATs contain only the catalytic GNAT domain and no accessory domain. Type V GNATs are composed of a tandem GNAT and a C-terminal region important for oligomerization and catalytic activity. Only the N-terminal GNAT domain is active, the central GNAT domain is important for structural integrity. **(C)** Several mechanisms contribute to catalytic activity of bacterial GNATs. Mammalian GNATs are shown to use a general base catalytic mechanism for acetyl-group transfer. In bacteria, not all GNATs contain a catalytic glutamate acting as general base and other mechanisms contribute to catalysis. The electrostatics in the active site might favor substrate amino group deprotonation. Some GNATs use a glutamate as general base for deprotonation of the substrate amino group. Other GNATs were reported to use a remote base, such as an activated serine residue to orient and polarize a catalytic water molecule acting as general base during catalysis. Other GNATs were reported to use an serine residue as catalytic base after activation by an active site water molecule. This catalytic strategy involves the formation of a serine-bound acetyl-enzyme intermediate. **(D)** Catalytic mechanism exerted by GNATs using a general base catalyst. Many GNATs use a catalytic glutamate as general base that abstracts a proton from the substrate amino group increasing its nucleophilicity for attack of the electrophilic carbonyl carbon of ac(et)yl-CoA. A tetrahedral intermediate is formed, which is resolved to yield the ac(et)ylated substrate amino group and CoA. For some GNATs an active site tyrosine contributes as catalytic acid resolving the tetrahedral intermediate by protonating the sulfhydryl group of the leaving CoA [figure redrawn and modified from Ali et al. (2018) and Blasl et al. (2021)].

The name GNAT for N-terminal acetyltransferases was derived from the enzyme Gcn5 in yeast, which was found to be an histone acetyltransferase (Brownell et al., 1996). So far more than 100,000 members of the GNAT family were discovered in eukaryotes, prokaryotes and archaea (Xie et al., 2014; Favrot et al., 2016). All protein lysine acetyltransferases (KATs) identified so far in bacteria belong to the GNAT family (Figure 2). However, this does not exclude that members of other families exist in prokaryotes and await their identification. The low level of sequence conservation (2–23%) makes the identification of novel protein acetyltransferases challenging and more members might be identified in the future (Vetting et al., 2005; Salah Ud-Din et al., 2016). The bacterial GNAT family comprises next to lysine acetyltransferases also N-terminal acetyltransferases and small molecule acetyltransferases (Xie et al., 2014). GNAT enzymes are structurally characterized by a topology containing six to seven β-strands and four α-helices (β_0_-β_1_-α_1_-α_2_-β_2_-β_3_-β_4_-α_3_-β_5_-α_4_-β_6_ (Figure 2A). GNATs are characterized by four conserved sequence motifs, A-D, which are arranged in the order C-D-A-B in the primary sequence (Figure 2A; Neuwald and Landsman, 1997; Dyda et al., 2000). Motif A encompasses α3 and β4, motif B α4 and β5, motif C α1 and β1 and motif D β2 and β3 (Salah Ud-Din et al., 2016). While motifs C and D were shown to contribute to stabilization of the GNAT fold, motifs A and B are directly involved in ac(et)yl-CoA/CoA and substrate binding. Motif A is often conserved in GNAT members and contains in the middle the consensus sequence Arg/Gln-x-x-Gly-x-Gly/Ala (x: any amino acid) (Wolf et al., 1998). This sequence motif is known as P-loop (phosphate-binding loop), which is involved in binding to acetyl-CoA/CoA pyrophosphate (Figure 2A; Salah Ud-Din et al., 2016). Different bacterial species encode for different number of acetyltransferases for ac(et)ylation of proteins at their lysine side chains and/or N-termini or for ac(et)ylation of small molecules. As an example, *Streptomyces* encodes for 72 acetyltransferases, while *E. coli* and *S. enterica* contain 26 genes with annotated GNAT gene product (Kawamoto and Ochi, 1998).

In *E. coli*, five protein lysine acetyltransferases were experimentally validated: PatZ, RimI, YjaB, YiaC, and PhnO (Christensen et al., 2018). All belong to the GNAT family. The KATs RimI and YjaB were structurally characterized by X-ray crystallography or nuclear magnetic resonance (NMR), respectively (Vetting et al., 2008; Lu et al., 2009). Mass-spectrometry was performed to identify potential substrates and/or interaction partners. These studies revealed that the KATs RimI and PhnO have a very narrow substrate range (11 and 10 potential substrate proteins, respectively), while for YiaC and YjaB a broader substrate spectrum was observed (391 and 171 potential substrates, respectively) (Christensen et al., 2018). In that context it is important to note that these substrates are determined by mass-spectrometry and further experiments are needed to validate those proteins as *bona fide* substrates. The *Mycobacterium tuberculosis* KAT RimI was reported to acetylate several peptides *in vitro*, which suggests that it has a broader substrate range as originally assumed (Pathak et al., 2016). However, these results obtained with peptides need to be validated to show if these are confirmed in the context of the natively-folded proteins. Along that line our laboratory showed that the three dimensional structure is important to determine sirtuin substrate specificity and the same might hold true also for substrate recognition by acetyltransferases (Knyphausen et al., 2016a). Bacterial acetyltransferases can be categorized into five types based on its domain organization and the arrangement of the GNAT domain (Figure 2B). These are discussed in the subsequent section.

## The Five Types of Bacterial GNAT Protein Acetyltransferases

### Type I and Type II GNATs Contain a Regulatory Nucleotide-Diphosphate-Forming acyl-CoA Synthetase Domain

Type I acetyltransferases encompass *E. coli* and *S. enterica* Pat. These enzymes are large enzymes (>80 kDa) composed of an N-terminal domain homologous to nucleotide-diphosphate (NDP)-forming acyl-CoA ligase/synthetase (~700 aa) but with lack in activity. At the C-terminus the enzymes contain a GNAT domain (~200 aa). For the *S. enterica* Pat (*Se*Pat) it was shown that the N-terminal domain binds to acetyl-CoA with a potential regulatory role on GNAT activity as mutations in the N-terminal domain impaired *Se*Pat activity (Thao and Escalante-Semerena, 2012). These mutations also cause alterations in the *Se*Pat structure as studied by circular dichroism suggesting that the N-terminal domain is important for the structural integrity of *Se*Pat (Thao and Escalante-Semerena, 2012).

The type II acetyltransferases are similar in size compared to type I GNAT enzymes but their domain organization is different, i.e., the NDP-forming acyl-CoA synthetase domain is located at their C-termini (~900 aa) while the GNAT catalytic domain (~200 aa) is located in their N-termini (Figure 2B). As described for class I enzymes also in class II enzymes the NDP-forming acyl-CoA synthetase domain is inactive due to the absence of a catalytic histidine residue. For many NDP-forming acyl-CoA synthetase domain containing proteins the formation of oligomers was reported (Marchler-Bauer et al., 2015; Yang et al., 2020). The type II enzyme, PatA from *Streptomyces lividans* (*Sl*PatA) contains a regulatory C-terminus with a collagen G-P-S motif that is important for its catalytic acetyltransferase activity. It is furthermore under debate if this C-terminal region is important for the observed oligomerization of *Sl*PatA.

Although the exact role of the regulatory NDP-forming acyl-CoA synthetase domain in bacterial class I and class II enzymes is not completely understood, all data suggest that it might be important for binding and sensing of acetyl-CoA to adjust the KAT activity to the cellular metabolic state. Moreover, it might be important for structural integrity of the enzyme and/or for allosteric regulation of the GNAT catalytic activity (Thao and Escalante-Semerena, 2012). For *S. enterica Se*Pat it was shown that it binds two molecules of acetyl-CoA, one molecule with the N-terminal and another with the C-terminal domain, respectively. Mutational analyses revealed that the N-terminal NDP-forming acyl-CoA synthetase domain is essential for the catalytic activity in *E. coli* PatZ and *Se*Pat (Thao and Escalante-Semerena, 2011; de Diego Puente et al., 2015).

### Type III GNATs Are Allosterically Regulated by Different Metabolic Molecules

Type III protein acetyltransferases encompass enzymes that are similar in the domain organization as class I enzymes (Figure 2B). However, they do not contain a NDP-forming acyl-CoA synthetase domain but instead a smaller regulatory domain (~300–400 aa) located at their N-termini. In *Mycobacterium smegmatis Ms*Pat (MSMEG_5458) and *M. tuberculosis Mt*Pat (Rv0998), a cAMP-binding domain precedes the GNAT domain (Supplementary Figures 1A, 2). The type III cAMP-GNAT from *Mycobacterium tuberculosis* H37Rv was structurally characterized by X-ray crystallography. The GNAT domain shows presence of the important sequence motifs, such as a glutamate that could act as catalytic base (MSMEG_5458: E234) and the motifs important for acetyl-CoA-binding (Nambi et al., 2010; Lee et al., 2012). The cAMP-binding domain strongly resembles cAMP-binding domains present in eukaryotic proteins such as protein kinase A (PKA) and its isoforms, in phosphodiesterases (PDEs), in cyclic nucleotide-gated ion channels (CNGs) and in the small G protein Rap guanine-nucleotide exchange factor (GEF) EPAC (exchange protein activated by cAMP) (Xu et al., 2011; Jager et al., 2012; Lee et al., 2012; Gancedo, 2013; Steegborn, 2014). Moreover, it is structurally highly similar and homologous to the cAMP-binding site of the CAP (catabolite gene activator protein)/CRP (cAMP responsive protein) from *E. coli* suggesting that the cAMP-binding sites are evolutionary related (Supplementary Figure 1A; Weber et al., 1982). In CAP/CRP, PKA, and EPAC, binding of cAMP to the regulatory subunits or the allosteric cAMP-binding site, results in conformational changes ultimately leading to activation of protein function (Beebe, 1994; de Rooij et al., 2000; Rehmann et al., 2003a, b; Rehmann, 2006, 2017; Harper et al., 2008). In analogy, it was shown that cAMP results in allosteric activation of *Ms*Pat (MSMEG_5458) activity as detected by assessing acetylation of the identified physiological substrate protein USP (universal stress protein) in presence/absence of cAMP (Nambi et al., 2010, 2013; Xu et al., 2011; Lee et al., 2012). Notably, mutation of the catalytic glutamate E234 to alanine in *M. smegmatis Ms*Pat (MSMEG_5458) resulted in a reduction in its catalytic activity, while cAMP-binding was not affected by the mutation (Nambi et al., 2010). However, the activity was not completely abolished in the E234A mutant and could be restored upon addition of cAMP. This shows that other mechanisms than the presence of the supposed catalytic base glutamate contribute to activation of the substrate lysine for acetyl-group transfer.

Another domain identified in type III acetyltransferases is the ACT (ACT: aspartate kinase, chorismate mutase, TyrA) domain preceding the C-terminal GNAT domain (Figure 2B). ACT-GNAT acetyltransferases are so far only identified in actinomycetes (Hentchel and Escalante-Semerena, 2015). ACT domains are found in many enzymes involved in regulation of the metabolism, such as in amino acid and purine biosynthesis. ACT domains are structurally adopting a ferredoxin-like βαββαβ topology of which two pairs form an eight-stranded antiparallel β-sheet which is flanked by the four α-helices at one side (Chipman and Shaanan, 2001). These domains bind to small regulatory ligands mostly amino acids. For the PatB enzyme from *Streptomyces lividans* (*Sl*PatB) and *Micromonospora aurantiaca* (*Ma*PatB; Micau_1670) and other acetyltransferases in actinobacteria it was shown that binding of L-Cys, L-Arg, and/or L-Asn improved its capacity to acetylate the acetyl-CoA-synthetase (Acs) (Xu et al., 2014; Lu et al., 2017). Bioinformatics analyses suggested the presence of more than 150 potential ACT-GNAT acetyltransferases to be encoded by actinobacteria (Lu et al., 2017). Why this type of GNATs is so broadly distributed in actinobacteria needs further investigation.

Finally, another type III enzyme was identified in *Myxococcus xanthus*, the protein acetyltransferase *Mx*Kat. *Mx*Kat was shown to sense NADP^+^ with its N-terminal domain preceding the GNAT domain (Figure 2B and Supplementary Figures 1B, 2; Liu et al., 2015). As also observed for cAMP and amino acid sensing KATs, also in the NADP^+^ sensing KAT *Mx*Kat, binding of the ligand NADP^+^ to the N-terminal domain allosterically regulates its acetyltransferase activity (Liu et al., 2015). The primary sequence and structural modeling suggests that the NADP^+^-binding domain adopts a typical Rossmann-fold consisting of two repeats of the topology β-α-β-α-β forming a six-stranded parallel β-sheet with two α-helices on each site of the sheet (Liu et al., 2015; Supplementary Figure 1B). Interestingly, binding of NADP^+^ but not of NAD^+^, NADH or NADPH to the N-terminal Rossmann-fold domain of *Mx*Kat resulted in inhibition of the acetyltransferase activity rather than activation as observed upon ligand binding for cAMP- and ACT-GNATs (Supplementary Figures 1, 2; Liu et al., 2015).

### The Type IV GNATs Are Almost Exclusively Formed by the GNAT Domain

Type IV GNATs encompass most bacterial acetyltransferases (Figure 2B). These enzymes do not contain any regulatory domain and consist almost entirely only of the GNAT domain. Examples for the class IV acetyltransferases are the recently identified novel *E. coli* lysine acetyltransferases RimI, YiaC, YjaB, and PhnO, *Rheudopseudomonas palustris Rp*Pat and *Mycobacterium smegmatis Ms*Pat (Christensen et al., 2018).

Notably, sometimes the classification of GNATs into type III or IV is not straightforward. As an example, the acetyltransferase AcuA in *B. subtilis* (*Bs*AcuA) is encoded in the *acuABC*-operon, which was originally identified to be important for acetoin and butanediol metabolism (Grundy et al., 1993, 1994). AcuC is a classical Zn^2+^-dependent deacetylase with structural homology to mammalian HDACs containing all important sequence motifs important for catalytic activity. The acetyl-CoA synthetase AcsA in *Bacillus subtilis* (*Bs*AcsA) is encoded in reverse orientation upstream of the *acuABC*-operon and *Bs*AcuA was shown to acetylate and inactivate *Bs*AcsA and *Bs*AcuC to deacetylate and activate *Bs*AcsA (Gardner et al., 2006). The exact role of *Bs*AcuB is not known. Interestingly, structural modeling with Phyre2 reveals homologies to an ACT domain in the N-terminus and a CBS (cystathionine beta synthase)-domain in the C-terminus (Supplementary Figure 1C; Kelley et al., 2015). The CBS domain is found in enzymes binding to adenosyl-group containing molecules such as S-adenosylmethionine or ATP. To this end, *Bs*AcuB might directly affect *Bs*AcuA and/or *Bs*AcuC function. This needs additional investigation. The fact that *Bs*AcuA activity might also depend on a regulatory ACT domain, albeit not present in one polypeptide chain but supplied *in trans* by *Bs*AcuB, makes a direct classification of *Bs*AcuB in either type III or type IV difficult. Based on the domain organization the *B. subtitlis* GNAT *Bs*AcuA might belong to class IV. However, if AcuB is important for AcuA activity, it could also be classified into class III, although the regulatory subunit is supplied *in trans* rather than *in cis.* Future studies are needed to clarify the role of *Bs*AcuB for *Bs*AcuA and/or *Bs*AcuC function (Gardner et al., 2006).

### Type V GNATs Are Tandem GNAT Proteins

Finally, type V GNATs encompasses acetyltransferases that contain a dual arrangement of GNAT domains as exemplified on the KAT Eis (enhanced intracellular survival) from *Mycobacterium tuberculosis* (Figure 2B). The two GNAT domains cover the N-terminal and central part of the enzyme and at the C-terminal region folds into a five-stranded β-sheet that is surrounded by four α-helices on one side. The crystal structure of Eis shows that it forms a hexamer in solution (Chen W. et al., 2011). The C-terminus is directly involved in catalysis. As the central GNAT domain lacks an Arg involved in binding to CoA phosphates it is likely that it does not show catalytic activity (Chen W. et al., 2011). However, the central GNAT domain might be important for the overall fold of the protein and for the oligomeric state and might therefore also indirectly be needed for full activity of the N-terminal GNAT domain (Chen W. et al., 2011). Evolutionary this domain arrangement might be developed by gene duplication. It resembles the domain organization and function of mammalian KDAC6, which also contains two catalytic domains of which only the C-terminal domain is highly active while the presence of the N-terminal domain stimulates the activity of the C-terminal domain *via* affecting the protein structural integrity (Zou et al., 2006; Boyault et al., 2007). Eis was shown to act as lysine acetyltransferase for mitogen-activated protein kinase-phosphatase 7 (MKP-7) and additionally as small molecule acetyltransferase acetylating and thereby inactivating aminoglycoside antibiotics. Both activities are important for suppression of the host cell response upon *M. tuberculosis* infection (Lella and Sharma, 2007; Zaunbrecher et al., 2009; Chen W. et al., 2011; Houghton et al., 2013).

Apart from presence of different regulatory domains all bacterial protein acetyltransferases use similar catalytic strategies to achieve acyl-group transfer to the substrate amino-group as explained in the next paragraph.

## Catalytic Strategies Exerted by Bacterial Protein GNATs

All bacterial acetyltransferases identified in bacteria show structural homologies to the mammalian GNAT acetyltransferases (Figure 2A). This class encompasses enzymes with specificity for proteins N-(ε)- or N-(α)-amino groups. A similar catalytic mechanism including formation of a ternary complex between ac(et)yl-CoA, enzyme and substrate is assumed also for the bacterial enzymes (Favrot et al., 2016).

In the mammalian GNAT enzymes and in several bacterial protein GNAT enzymes, a catalytic glutamate is suggested to be involved as catalytic base to abstract a proton from the substrate amino group [either N-(α)- or N-(ε)-amino group] to increase its nucleophilicity for attack of the electrophilic ac(et)yl carbonyl group in ac(et)yl-CoA (Figure 2D; Friedmann and Marmorstein, 2013; McCullough and Marmorstein, 2016). However, to achieve complete abolishment of the catalytic activity *in vitro*, single mutation of the catalytic glutamate is not sufficient for human KAT2A (Gcn5) and KAT2B (pCAF) so that additionally a conserved aspartate is often mutated (KAT2A: E575 and D615; KAT2B: E570 and D610) (Orpinell et al., 2010; Fournier et al., 2016; Blasl et al., 2021). Data on *S. cerevisiae Sc*Gcn5 suggested that the sole mutation of E173A (analog to E575/E570 in human *Hs*Gcn5/*Hs*pCAF) resulted in defects in transcriptional activation *in vivo* and histone acetylation *in vitro* while mutation of *Sc*Gcn5 D215 (analog to D615/D610 in *Hs*pGcn5/*Hs*pCAF) had almost no effects. This suggests that E173 in *Sc*Gcn5 (E570 in *Hs*Gcn5) is the important residue for catalysis (Kuo et al., 1998; Wang et al., 1998). Mechanistically, the additional contribution of *Sc*Gcn5/*Hs*Gcn5/*Hs*pCAF D214/D615/D610 in catalysis might be mediated by its impact on the electrostatics in the active site favoring deprotonation of the substrate amino group, by mediating substrate association *via* long rage electrostatic steering or just by affecting substrate binding (Figure 2C).

Inspection of the data reported for different members of bacterial GNAT acetyltransferases shows that these might use slightly different catalytic strategies several of which might contribute to certain extend to achieve efficient ac(et)yl-group transfer: (a) different residues can act as general base, such as glutamate or serine acting directly as base or indirectly as remote base activating a water molecule, (b) the enzymes C-terminal carboxylate can activate a catalytic water for nucleophilic attack, (c) the active site electrostatics is used to achieve deprotonation of the substrate amino group, (d) a catalytic tyrosine residue can act as general acid to protonate the CoA sulfhydryl group for collapse of the tetrahedral intermediate, (e) binding of second messengers such as cAMP or amino acids *via* accessory domains (cAMP-binding domain, ACT domain) can modulate enzyme activity, and/or (f) multimerization induced by auto-ac(et)ylation, ac(et)yl-CoA-binding or by intermolecular interactions is needed for full enzymatic activity (Figure 2C). In the following section these strategies are explained describing important examples.

### Oligomerization Contributes to Catalytic Activity as a Postulated Catalytic Base Glutamate Is Not Sufficient for Efficient Catalysis

The mutation of the postulated conserved catalytic glutamate (E809) in *E. coli* PatZ (also known as YfiQ or Pka) and *S. enterica Se*Pat did reduce catalytic activity but it did not switch off catalytic activity completely. Furthermore, *Se*Pat E809Q was not defective *in vivo* suggesting a catalytic mechanism without this glutamate acting as general base (de Diego Puente et al., 2015). It was reported for *Se*Pat but not for PatZ that this E809 is important for Pat structure rather than for catalysis (Thao and Escalante-Semerena, 2012). Also for other GNAT KATs it was shown that a catalytic glutamate is either missing or not important for catalysis and it is suggested that the deprotonation of the substrate lysine is conducted by other residues such as active site histidine residues or that the positive electrostatics in direct vicinity of substrate lysine side chain lowers its pK_a_ value favoring deprotonation and increasing its nucleophilicity (Angus-Hill et al., 1999; Hickman et al., 1999; de Diego Puente et al., 2015). In fact, a more basic pH could restore the catalytic activity of *E. coli* PatZ E809A (de Diego Puente et al., 2015). Several bacterial KATs were shown to possess auto-ac(et)ylation activity. For *E. coli* PatZ it was reported it forms a tetramer in solution which is independent of acetyl-CoA-binding (de Diego Puente et al., 2015). However, auto-ac(et)ylation of lysine residues in the N-terminal domain and the catalytic GNAT domain induces oligomerization into an octamer increasing its catalytic activity (de Diego Puente et al., 2015). For *E. coli* PatZ the N-terminal domain is essential for enzymatic activity as the isolated GNAT domain was catalytically inactive (de Diego Puente et al., 2015). For the *S. enterica* enzyme Pat (*Se*Pat) acetyl-CoA-dependent oligomerization was reported from a monomer form to a tetramer. In analogy to *E. coli* PatZ also for *Se*Pat this effect was due to acetyl-CoA-binding to the N-terminal NDP-forming acyl-CoA synthetase domain (Thao and Escalante-Semerena, 2011). Also this oligomerization resulted in an increase in its activity. For both enzymes a positive cooperativity was observed for acetyl-CoA-binding resulting in an increase in acetyltransferase activity, suggesting that this might be a general mechanism observed in these bacterial class I GNAT enzymes (de Diego Puente et al., 2015). Notably, also for the class II GNAT Pat from *Streptomyces lividans* formation of a higher oligomer, an octamer, was observed. It is not clear if this is driven by ac(et)ylation, acyl-CoA-binding or by a different mechanism postulated, i.e., by intermolecular interactions driven by the affinity of the collagen signature G-P-S that was found in the C-terminus and as being essential for its catalytic activity (Tucker and Escalante-Semerena, 2013, 2014).

Future studies will show if and which residues are important to act as general base during catalysis of PatZ, *Se*Pat and other class I and class II enzymes. As stated above, oligomerization is an important mechanism to achieve full activity in class I and class II enzymes. To this end, oligomerization might induce conformational changes in the enzymes structure that are essential for the correct arrangement of the catalytic machinery into a competent state for catalysis or for substrate binding. To fully understand, how these enzymes achieve substrate acetylation, structural data of the full length PatZ, *Se*Pat, or *Sl*Pat will be needed.

The type III enzymes contain N-terminal domains, e.g., cAMP-binding domains or ACT-domains, that mediate oligomerization and regulate the enzymatic activity. Binding of cAMP to the N-terminal domain of *Mycobacterium smegmatis Ms*Pat was shown to increase the catalytic activity (Xu et al., 2011; Nambi et al., 2013; Podobnik et al., 2014). This was due to inducing a huge conformational change in the protein upon cAMP-binding that relives autoinhibition and replaces a pseudo-substrate sequence from the protein-substrate binding site. This mechanism reminds of regulation of PKA function and EPAC activation. For the cAMP-regulated protein acetyltransferase from *Mycobacterium smegmatis Ms*Pat MSMEG_5458 it was shown that is has a catalytic core which resembles other GNAT proteins (Podobnik et al., 2014). The suggested catalytic base E235, contributes to catalysis. However, mutation of the catalytic glutamate E235 to alanine in *M. smegmatis* MSMEG_5458 resulted only in a reduction in its catalytic activity. The binding to cAMP was not affected by the mutation (Nambi et al., 2010). However, the activity of MSMEG_5458 E235A was not completely abolished and was restored upon addition of cAMP. This shows again that other mechanisms than the glutamate contribute to catalysis. Mutation of further residues lining the substrate lysine binding site, such as R223 and V225, in *M. tuberculosis Mt*Pat Rv0998, also impaired the catalytic activity (Lee et al., 2012). Studies using different acyl-CoA as donor molecules for ac(et)yl transfer, such as propionyl-CoA and butyryl-CoA, showed that *Mt*Pat Rv0998 has a high degree of acyl-chain promiscuity as it can efficiently turn over diverse acyl-group donor molecules (Nambi et al., 2013). This might reflect the role of the cAMP-binding site to precisely control promiscuous *Mt*Pat Rv0998 activity dependent on the prevalence of cellular cAMP, which is a second messenger produced by adenylyl-cyclase (Lee et al., 2012). Functionally, *Mt*Pat was shown to regulate fatty acid and propionate metabolism (Nambi et al., 2013).

As stated above, ACT-domain containing GNATs were only found in actinomycetes so far. Binding of amino acids such as L-Cys, L-Arg, and/or L-Asn was shown to allosterically activate GNAT activity (Xu et al., 2014; VanDrisse and Escalante-Semerena, 2018). ACT-GNATs are involved in regulation of amino acid and purine metabolism. Structural alignment shows that the ACT-GNAT *Ma*PatB (Micau_1670) from *Micromonospora aurantiaca* and the *Streptomyces lividans Sl*PatB (EFD70633) have a glutamate at the analogous position to *Hs*pCAF/*Hs*Gcn5 suggesting that this act as catalytic base during catalysis for deprotonation and activation of the substrate amino group (Xu et al., 2014; VanDrisse and Escalante-Semerena, 2018). For both enzymes the acetyl-CoA synthetase (Acs) was shown to be a substrate (Xu et al., 2014).

In contrast, the type III enzyme *Myxococcus xanthus Mx*PAT (Mxan_3215) that contains a Rossmann-fold domain for specific NADP^+^-binding N-terminally to the GNAT domain is negatively regulated by NADP^+^, i.e., the acetyltransferase activity is inhibited upon NADP^+^-binding (Supplementary Figure 1B). For the enzyme *M. xanthus Mx*PAT (Mxan_3215) (Phyre2: V116/S156 are at position E570/D610 in *Hs*pCAF) no catalytic glutamate is present as shown by primary sequence alignment or by structural modeling with known catalytic base glutamate containing protein GNATs (Liu et al., 2015). This suggests that also for the NADP^+^-GNAT enzymes other strategies are employed to catalyze acetyl-group transfer. To really show how these NADP^+^ regulated acetyltransferases catalyze the acetyl transfer structural data including complexes with/without NADP^+^, substrate and acetyl-CoA/CoA is needed.

All of these type III acetyltransferases share a similar mode of action. Binding of a ligand, such as NADP^+^, amino acids or cAMP to the N-terminal domain results in allosteric regulation of the GNAT catalytic activity. The outcome can be activation of acetyltransferase activity as observed for ACT-domain containing and cAMP-binding GNATs or also inactivation of the acetyltransferase activity as observed by the NADP^+^-binding GNATs. For all of these enzymes, binding to the ligands binding site is resulting in conformational changes that affect acetyltransferase activity maybe by affecting substrate binding, by altering the electrostatics of the active site or the arrangement of the catalytic machinery in a more/less competent state for catalysis. For all of these type III enzymes, this mode of regulation allows a tight control of the GNAT activity to the availability of the ligands, which are direct indicators of the cellular metabolic state.

### An Active Site Tyrosine That Might Act as General Acid Is Essential for Catalysis

**FIGURE 3:**
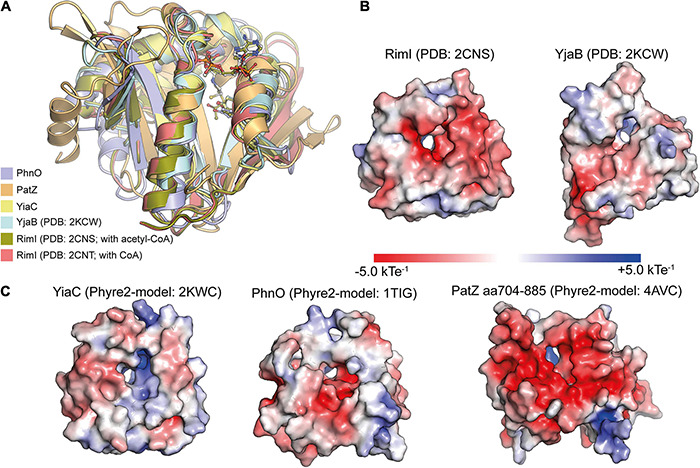
Structural characterization of *E. coli* GNAT protein acetyltransferases. **(A)** *E. coli* GNAT domains of protein acetyltransferases are structurally very similar. For the KATs PhnO, PatZ (aa704-885) and YiaC, Phyre2 models were created. These were superimposed with the structurally characterized KATs RimI (PDB: 2CNS and 2CNT) and YjaB (PDB: 2KCW). The catalytically important tyrosine residue suggested to act as general acid supporting resolving of the tetrahedral intermediate superimposes well. The KATs show a high degree of structural similarity showing root-mean-square-deviations (RMSD) between 0.064 and 2.319 Å toward YiaC [structural models were created with Phyre2 (Kelley et al., 2015); the figure was generated with PyMOL v.2.3.4) (Schrödinger LLC, 2000)]. **(B,C)** The APBS Electrostatics plugin in PyMOL was used to plot the electrostatic potential on the surfaces of the experimentally determined structures of the KATs RimI (PDB: 2CNS) and YjaB (PDB: 2KWC) **(B)** or the Phyre2-generated structural models of YiaC, PhnO, and PatZ **(C)** (Jurrus et al., 2018). All structures were oriented toward the binding site. The electrostatics on the substrate binding area differs considerably suggesting that these KATs use diverse substrates. Moreover, also the electrostatics within the active site differs suggesting that it might support catalysis to different extent. The figure was generated with PyMOL v.2.3.4 (Schrödinger LLC, 2000).

The class IV enzymes, RimI, YiaC, YjaB, and PhnO were recently shown to act as N-(ε)-lysine acetyltransferases using an elegant experimental system (Christensen et al., 2018). An *E. coli* gutted strain was engineered that carried genomic deletions in genes *pta*, encoding phosphotransacetylase needed for biosynthesis of acetyl-phosphate, in *cobB*, encoding the sirtuin deacetylase CobB, in *acs*, encoding acetyl-CoA synthetase and in *patZ* (*yfiQ*), encoding acetyltransferase PatZ (Christensen et al., 2018). This strain has a reduced systemic background in non-enzymatic acetylation and it should furthermore accumulate acetylation catalyzed by different novel KATs as the deacetylase CobB is absent. This enabled the identification of RimI, YjaB, YjaC, and PhnO as novel protein lysine acetyltransferases. To validate the activities catalytic mutants were created. These mutants were selected based on structural models (Christensen et al., 2018). For switching off catalytic activity, either the supposed general base glutamate in PhnO, or a tyrosine residue present in all four KATs, was selected that is supposed to act as general acid to protonate the CoA sulfhydryl group to resolve the tetrahedral reaction intermediate (Figure 2D). For PhnO mutation of either the suspected catalytic glutamate (E78A) or the tyrosine (Y128A) completely abolished systemic acetylation of whole cell lysate. For RimI, YiaC, and YjaB the observed systemic acetylation activity was abolished with mutation of the postulated general acid tyrosine (Christensen et al., 2018). Structural analyses using the experimentally determined structures of RimI and YjaB and models obtained with Phyre2 show that these GNATs are structurally very similar (Figure 3A). The hydroxyl groups of a tyrosine (YjaB: Y117, RimI/YjaC: Y115, PhnO: Y128) points toward the acetyl-group of acetyl-CoA substrate and might therefore have a role for substrate binding or during catalysis as general acid as suggested (Christensen et al., 2018). To finally judge this, a structure of the ternary complex consisting of GNAT, substrate and acetyl-CoA would be desirable. This Tyr is replaced by Phe in *Hs*Gcn5 and *Hs*pCAF. An acidic residue acting as general base cannot be identified in the novel KATs RimI and YiaC (RimI: T65; YiaC: A72) suggesting that another catalytic mechanism is used by these protein GNATs maybe involve the acidic active site electrostatics, other residues or a catalytic water molecule acting as nucleophile for catalysis (Figures 3B,C). The Thr65 in RimI could act as remote base by activating a water molecule for catalysis. A similar mechanism has been observed for GNATs involving a serine residue (Figure 2C; Ud-Din et al., 2015). For the KATs PhnO and YjaB an acidic residue that would have the capacity to act as general base is present as found by alignment of their structures and their primary sequences with *Hs*pCAF and *Hs*Gcn5 (YjaB: D74, PhnO: E78). Notably, although RimI and YjaB are dimeric, their active sites are at the opposite site of the dimer interface suggesting that oligomerization does not directly influence their activity. However, for human GNAT enzymes presence in multi-protein complexes was shown which also affects substrate specificity and catalytic activity. If these novel KATs are present in complexes with other proteins and if this affects the catalytic activity and/or substrate specificity needs further investigation. Moreover, also substrate binding might result in an arrangement of the catalytic residues into a competent state for catalysis in an induced fit mechanism as was also proposed for human HAT1 (Wu et al., 2012).

Recently, a novel catalytic mechanism was reported for the *P. aeruginosa* PA3944 GNAT, which shows activity toward polymyxin antibiotics (Baumgartner et al., 2021). This catalytic mechanism involves a catalytic serine residue directly acting as nucleophile resulting in the formation of a covalent acyl-enzyme intermediate (Figure 2C). It was shown that the glutamate originally regarded as catalytic base plays a role in substrate recognition or stabilization (Baumgartner et al., 2021). If this mechanism also applies to certain GNAT protein acetyltransferases needs further investigation.

### The GNAT C-Terminal Carboxylate Acts as Remote Base Activating an Active Site Water

For the enzyme Eis of *Mycobacterium tuberculosis* (*Mt*Eis), belonging to type V GNATs, the catalytic mechanism to acetylate aminoglycosides was analyzed biochemically and structurally (Chen W. et al., 2011; Houghton et al., 2013). Eis forms a hexamer in solution and only the N-terminal GNAT domain is active as shown by mutational studies (Chen W. et al., 2011; Houghton et al., 2013). As shown for the NADP^+^-GNATs, also Eis does not use a catalytic glutamate for catalysis. Instead *Mt*Eis activates a catalytic water, the hydroxide acting as general base, by the C-terminal α-carboxyl group of Phe402 acting as remote base (Chen W. et al., 2011). This water molecule is coordinated by His119 *via* its main chain amide, and it furthermore orients the aminoglycosides amino group *via* its main chain carbonyl group. Catalysis proceeds *via* formation of a tetrahedral intermediate which is resolved by a tyrosine, Tyr126 in *Mt*Eis, acting as general acid protonating the CoA sulfhydryl group for collapse of the intermediate (Figure 2D; Chen W. et al., 2011). Notably, the substrate binding cavity is highly negatively charged to allow an efficient electrostatic attraction of the positively charged (poly)amine group containing aminoglycosides at physiological pH.

## N-Terminal Acetyltransferases in Bacteria

The enzymes RimI, RimJ, and RimL were reported as protein N-(α)-acetyltransferases toward the ribosomal proteins S18, L5 and S12, respectively (Yoshikawa et al., 1987; Tanaka et al., 1989). RimI was furthermore shown to N-(α) acetylate the amino termini of GroEL1 and GroS/GroES in *Mycobacterium tuberculosis* (Pathak et al., 2016). Recently, RimI was additionally shown to act as N-(ε)-lysine acetyltransferase (Christensen et al., 2018). The acetyltransferase RimL, but neither RimJ nor RimI, was furthermore shown to acetylate the peptide antibiotic microcin C (McC) in *E. coli*, providing some resistance to the translation inhibitor McC (Kazakov et al., 2014). While in eukaryotes N-terminal acetylation is widespread and almost 80% of all human proteins carry an N-terminal acetylation, it is less prevalent in bacteria. In fact, recent systemic mass spectrometric data obtained with *Mycobacterium tuberculosis*, *Acinetobacter baumannii*, and *Pseudomonas aeruginosa* showed that approximately 10% of the proteins were N-terminally acetylated (Ouidir et al., 2015; Kentache et al., 2016; Thompson et al., 2018). However, it must be noted that this might be species dependent and N-terminal acetylation has to be studied systematically in bacterial physiology. It is likely that more N-terminal acetyltransferases and N-terminally acetylated proteins might be detected in the future. Most performed systemic mass spectrometric analyses did not focus on N-terminal acetylation and might therefore miss many sites. Moreover, N-terminal acetylation might also be dependent on the physiological state. In contrast to reported post-translational N-terminal acetylation in bacteria, in eukaryotes, N-terminal acetylation occurs both, post-translationally and co-translationally. In eukaryotes, N-terminal acetylation was reported to affect protein folding, protein-protein interactions, protein-membrane recruitment and protein turnover *via* the N-end rule pathway affecting recruitment of ubiquitin E3 ligases targeting the protein for proteasomal degradation (Soppa, 2010; Nguyen et al., 2018). Which role N-terminal acetylation has in bacterial physiology and if it has similar roles as those described in eukaryotes must be studied in the future.

A dual function for GNATs as N-(α)-/N-(ε)-acetyltransferase is known for several acetyltransferases. As stated above, RimI is active in acetylation of N-(ε)-amino groups of lysine side chains and also in acetylation of N-terminal amino groups in proteins acting additionally as N-(α)-acetyltransferase. Along that line, also for the reported lysine acetyltransferase YiaC an activity as N-terminal acetyltransferase was recently shown suggesting a role of N-terminal acetylation in bacteria (Christensen et al., 2018; Parks and Escalante-Semerena, 2020). YiaC was shown to act as N-terminal acetyltransferase for the long isoform of CobB in *Salmonella enterica* impairing CobB deacetylase activity (Parks and Escalante-Semerena, 2020). Notably, also in plastids of plants a novel family of GNAT protein acetyltransferases was discovered with a dual N-terminal and lysine acetyltransferase activity suggesting that these enzymes developed during evolution prior to development of photosynthetic plants (Bienvenut et al., 2020).

Importantly, so far no deacetylase for N-terminal acetylation was identified neither in mammals nor in bacteria. Chemically it is surprising that no enzyme has been discovered so far that is capable to remove N-terminal acetyl groups. In contrast to lysine acetylation that can be removed by deacetylases and which is reversible, this makes an N-terminal acetylation irreversible. In eukaryotes, further acylations, such as myristoylation and propionylation, occur on N-terminal amino groups affecting processes such as subcellular localization or protein-protein interactions (Foyn et al., 2013; Udenwobele et al., 2017). Future studies will show if also bacterial protein N-termini are modified by diverse acylations exerting functionally different roles.

## The Bacterial Sirtuin Deacetylases Use NAD^+^ as Co-Substrate for Catalysis

**FIGURE 4:**
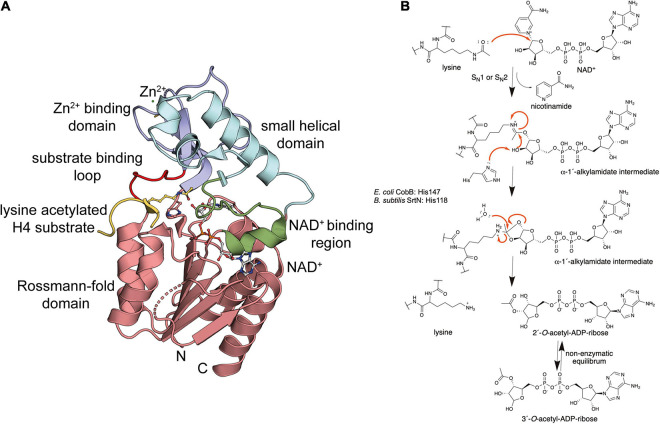
Bacterial sirtuins are NAD^+^-dependent lysine-deac(et)ylases. **(A)** Structure of the *E. coli* sirtuin deacetylase CobB in complex with an lysine acetylated human histone 4 substrate (PDB: 1S5P). The CobB structure was superimposed to a structure of human SIRT2 in complex with NAD^+^ (PDB: 4RMG) to show localization of NAD^+^. CobB contains a Rossmann-fold domain (salmon) composed of a six-stranded parallel β-sheet flanked by several α-helices containing the NAD^+^-binding site. An NAD^+^-binding region (green) contributing to NAD^+^-binding connecting Rossmann-fold and small helical domain (light blue). The Zn^2+^-binding domain (dark blue) contains a structural Zn^2+^-ion that is coordinated by two pairs of conserved Cys-residues (only two are visible in this structure). The substrate binding loop (red) connects the Zn^2+^-binding domain and the Rossmann-fold domain [the figure was generated with PyMOL v.2.3.4 (Schrödinger LLC, 2000)]. **(B)** Catalytic mechanism exerted by sirtuins. Sirtuins are NAD^+^-dependent lysine deac(et)ylases enzymes that use NAD^+^ as stoichiometric co-substrate for catalysis. Initially, the carbonyl-oxygen of the lysine’s ac(et)yl-group as a nucleophile performs an attack of the electrophilic C-1′ of the NAD^+^ ribose. This results in fast release of nicotinamide subsequent formation of a C-1′-*O*-alkylamidate intermediate. Several steps are needed to resolve this intermediate. These include a hydrolysis step, as shown, resulting in formation of the deac(et)ylated lysine and 2′-*O*-acetyl-ADP-ribose, which exists in a non-enzymatic equilibrium with 3′-*O*-acetyl-ADP ribose [figure redrawn and modified from Smith and Denu (2006), Ali et al. (2018), Teixeira et al. (2020), and Blasl et al. (2021)].

Most Gram-negative and Gram-positive bacteria encode for one or two sirtuins (SIRT: silent information regulator) (Poulose and Raju, 2015). This low number suggests that bacterial sirtuins either control specific physiological processes having a very narrow substrate range or that these are evolutionary developed to show a high degree of substrate promiscuity. The latter would imply that bacterial sirtuins act as detoxifying enzymes to remove systemic lysine acylation occurring if acyl-CoA is accumulating under conditions such as metabolic fuel switching. Notably, an enzyme with overall high level of substrate promiscuity might also have specific substrates which are more efficiently converted or for which substrate specificity is created by subcellular localization or by transcriptional regulation of their expression levels. Amongst the genome-sequenced bacteria several obligate intracellular pathogenic bacteria such as species from the genera *Rickettsia*, *Chlamydia*, *Mycoplasma*, and *Borrelia* lack a sirtuin encoding gene (Greiss and Gartner, 2009). Bacterial sirtuins were structurally and functionally characterized. The sirtuins CobB from *E. coli* and SrtN from *B. subtilis* are the best studied bacterial sirtuins. Bacterial sirtuins are as their eukaryotic counterparts NAD^+^-dependent protein deacetylases (Gardner and Escalante-Semerena, 2009). Most sirtuins catalyze NAD^+^-dependent deacylation of lysine side chains in proteins resulting in formation of nicotinamide, 2′/3′-*O*-acetyl-ADP-ribose and the deacylated substrate (Figure 4B and Supplementary Figure 2; Gardner and Escalante-Semerena, 2009). For some sirtuins also mono-ADP-ribosyltransferase (MARylation) activity was described. For mammalian sirtuins this activity is often less efficient compared to the sirtuin deacylase activity questioning the physiological significance of this activity (Hawse and Wolberger, 2009; Blasl et al., 2021).

### Classification of Prokaryotic Sirtuins

CobB was originally identified in *S. typhimurium* LT2 to act in cobalamin biosynthesis and propionate catabolism (Tsang and Escalante-Semerena, 1996). It was shown that CobB possesses weak ADP-ribosyltransferase activity and later CobB was found to have robust NAD^+^-dependent deacetylase activity for acetyl-CoA synthetase in *S. enterica* (Frye, 1999; Imai et al., 2000; Landry et al., 2000; Smith et al., 2000; Starai et al., 2002). Sirtuins were first described in budding yeast *Saccharomyces cerevisiae* and it was shown that the yeast SIR2 had a strong impact on replicative lifespan (Kaeberlein et al., 1999). Later, it was shown that SIR2 is an NAD^+^-dependent lysine deacetylase (Imai et al., 2000). In mammals, sirtuins are classified into class III protein deacetylases, while classes I, II and IV are the classical Zn^2+^-dependent lysine deacetylases (KDACs). As KDACs were initially identified as histone deacetylases they were originally named as histone deacetylases (HDACs). Humans encode seven sirtuins that are categorized based on phylogenetic analyses. Eukaryotic sirtuins within class III of lysine deacetylases were classified in sirtuin subclasses I-IV (Olesen et al., 2018). Subclass Ia contains SIRT1, that has a robust deacetylase activity and it primarily located in the nucleus, subclass Ib encompasses the robust lysine deacetylases SIRT2 (cytosolic) and SIRT3 (mitochondrial). The other subclasses show catalytic activities with different acyl-chain preferences (Blasl et al., 2021). The subclass II enzyme SIRT4 is mitochondrial and it was shown to possess weak deacetylase activity but removes longer acyl-chains from lysine side chains and it has mono-ADP-ribosyltransferase (MAR) activity (Frye, 1999; Ahuja et al., 2007). The subclass III enzyme SIRT5, which is also localized to the mitochondrial matrix, has efficient lysine deacylase activity toward negatively charged acylations such as malonyl-, glutaryl-, and succinyl-groups. Subclass IV is divided in the subclass IVa enzyme SIRT6, localized in the nucleus with preferences for longer fatty acylated substrates and with reported MAR activity, and the subclass IVb enzyme SIRT7, which enriched in the nucleoli with activities as deacetylase, desuccinylase, and activity toward longer acyl chains (Liszt et al., 2005).

Phylogenetic analyses revealed that all archaeal sirtuins belong to subclasses III and U (U: undifferentiated) and the bacterial sirtuins are classified into subclasses II, III, M (M: macrodomain-linked), U and the subclass of Sir2-like sirtuins (Olesen et al., 2018). Most prokaryotic sirtuins belong to the mammalian sirtuin subclasses II and III, with subclass III containing the far most prokaryotic enzymes (Frye, 2000). Further bacterial sirtuins belong to subclass M, subclass U and to subclass of Sir2-like enzymes (Frye, 2000; Olesen et al., 2018). Subclass III genes are found in nearly all Gram-negative bacteria and in archaea suggesting that it either developed prior to the divergence of the domains during evolution or that the gene was transferred by lateral transfer from bacteria to archaea or vice versa (Frye, 2000). So far, no prokaryotic subclass I enzyme could be identified (Frye, 2000). The fifth subclass, subclass U, is found in several firmicutes, i.e., Gram-positive bacteria, such as *B. subtilis*, *Staphylococcus aureus*, and in the Gram-negative species *Thermotoga maritima* (Frye, 2000). These enzymes were classified into the separate subclass U as these enzymes show sequence motifs that are in between subclasses II/III and I/IV (Frye, 2000). The *B. subtilis* sirtuin SrtN was phylogenetically analyzed to also belong to subclass U (Gardner and Escalante-Semerena, 2009; Greiss and Gartner, 2009; Olesen et al., 2018). The catalytic activity of SrtN is similar to mammalian SIRT4 acting as lipoamidase (Ahuja et al., 2007; Anderson et al., 2017a; Rowland et al., 2017). Both, *E. coli* CobB and *B. subtilis* SrtN are active as lipoamidase affecting the lipoylation levels and activities of pyruvate dehydrogenase and α-ketoglutarate dehydrogenase as also reported for mammalian SIRT4 (Mathias et al., 2014; Rowland et al., 2017). Recently, the enzyme Sir2La from *Lactobacillus acidophilus* NCFM was identified as first subclass U sirtuin with dual activity as efficient deacylase, debutyrylase and depropionylase (Olesen et al., 2018).

The macrodomain-linked sirtuins SirTMs (subclass M) were found to be particularly present in pathogenic organisms such as pathogenic bacterial families including *Chlostridiaceae*, *Enterococcaceae*, *Lachnospiraceae*, *Spirochaetaceae*, and *Veillonellaceae* and in diverse pathogenic fungal families (Rack et al., 2015). They are encoded in an operon together with a macrodomain and in bacterial genera *Lactobacillus*, *Staphylococcus* and *Streptococcus* additionally with GcvH-L (glycine cleavage system H-like) and with LplA2 (lipoate protein ligase A).

### Substrate Preference of Bacterial Sirtuins

The *E. coli* enzyme CobB shows homologies and similar substrate preferences as mammalian SIRT5 (Du et al., 2011; Fischer et al., 2012). In analogy, it was reported that CobB possesses a robust deacetylase, de-2-hydroxyisobutyrylase, desuccinylase and demalonylase activity (Figure 1C; Peng et al., 2011; Colak et al., 2013; Dong et al., 2019). *E. coli* CobB was shown to act as deacetylase for acetyl-CoA synthetase (Acs) resulting in activation of Asc activity (Zhao et al., 2004). However, in contrast to SIRT5, which only possesses weak deacetylase activity, CobB shows comparable deacetylase and desuccinylase efficiencies (Zhao et al., 2004; Colak et al., 2013). Recently, CobB was shown be an efficient lipoamidase in analogy to mammalian SIRT4 acting on important lipoylated metabolic complexes, such as the α-ketoglutarate dehydrogenase (KDH) complex, the pyruvate dehydrogenase (PDH) complex and the glycine cleavage (GCV) complex and as de-2-hydroxyisobutyrylase modulating enolase activity (Rowland et al., 2017; Dong et al., 2019). Furthermore, CobB was shown to act as de-homocysteinylase and it is supposed to revert protein lysine propionylation (Mei et al., 2016; Sun et al., 2016).

SirTMs do not possess any deacylase activity, which is most likely due to the absence of the catalytic histidine residue (Rack et al., 2015; Olesen et al., 2018). The SirTMs from the Gram-positive bacterial pathogens *Staphylococcus aureus* and *Streptococcus pyogenes* possess an mono-ADP-ribosyltransferase (MAR) activity toward the also in the operon encoded protein GcvH-L. This activity is dependent on prior lipoylation catalyzed by LplA2 (Rack et al., 2015). The operon encoded macrodomains, such as YmdB from *E. coli*, are able to reverse the MARylation acting as MAR hydrolase (Rack et al., 2015). For some of these operon encoded macrodomains, such as YmdB from *E. coli* and MacroD from *S. aureus*, an activity as *O*-acetyl-ADP-ribose deacetylase was reported, resulting in formation of ADP-ribose and acetate (Supplementary Figure 2; Chen D. et al., 2011). Thereby, this constitutes a system to regulate the cellular levels of *O*-acetyl-ADP-ribose that is formed during sirtuin-catalyzed deacetylation. In mammalian cells ADP-ribose was shown to act as a second messenger amongst others acting on ADP-ribose-gated calcium channels (Russo et al., 1998; Perraud et al., 2001; Borra et al., 2002). What exactly the physiological role of the *O*-acetyl-ADP-ribose and/or ADP-ribose is in bacterial cells, if these act as signaling molecules or second messengers, needs further investigation. One possibility is that ADP-ribose is the physiologically active second messenger formed by deacetylation of *O*-acetyl-ADP-ribose originating from sirtuin catalysis as postulated for eukaryotes (Borra et al., 2002; Chen D. et al., 2011). Physiologically, SirTMs were shown to be involved in reactive-oxygen species (ROS) stress response in bacteria (Rack et al., 2015). If these operon encoded SirTMs have additional substrates needs further investigation.

## Structure and Catalytic Mechanism of Bacterial Sirtuins

Our knowledge on the structure, function and the catalytic mechanism of sirtuins is based on the successful structural characterization of all mammalian sirtuins and of bacterial sirtuins. These analyses revealed that bacterial sirtuins are structurally very similar to mammalian sirtuins supporting the same evolutionary origin (Frye, 2000; Smith et al., 2000; Greiss and Gartner, 2009; Olesen et al., 2018). Structures of various sirtuins of different organisms are solved by X-ray crystallography in their apo states and in complexes with NAD^+^, nicotinamide and substrate peptides (Zhao et al., 2003, 2004; Sanders et al., 2010; Du et al., 2011; Cao et al., 2015; Dai et al., 2015; Gai et al., 2016; Knyphausen et al., 2016a; You et al., 2017, 2019). CobB is a conserved sirtuin amongst prokaryotes and CobB from *E. coli* was the first bacterial sirtuin that was structurally characterized (Figure 4A; Zhao et al., 2004). Overall CobB is structurally composed of 9 α-helices and 10 β-strands that form 2 domains: a Rossmann-fold domain for NAD^+^-binding, and a Zn^2+^-binding domain (Zhao et al., 2004). The Rossmann-fold domain is structurally highly similar to the mammalian sirtuins, while the Zn^2+^-binding domain shows some variability compared to the archaeal and mammalian sirtuins (Figure 4A). This variability suggests that it plays a role in CobB specific functions such as subcellular localization, binding of other regulatory proteins or substrate binding (Zhao et al., 2004). The Rossmann-fold domain consists of a central parallel β-sheet composed of six β-strands, which is flanked by four α-helices on each side of the β-sheet. This fold is created by duplication of the topology β-α-β-α-β. The Zn^2+^-binding domain in CobB is composed of three antiparallel β-strands, a short β-strand and three α-helices (Figure 4A). The Zn^2+^-ion is not directly involved in catalysis but it is important for the structural integrity of the domain. It is coordinated by two pairs of conserved cysteine residues (Zhao et al., 2004; Spinck et al., 2020). The co-factor binding loop connects the Rossmann-fold domain and the Zn^2+^-binding domain (Figure 4A). This loop is directly involved in NAD^+^-binding. Upon binding of NAD^+^ the flexible loop gets ordered and the sirtuin adopts an ordered conformation compatible to bind to the substrate. The substrate recognition is achieved with residues on the surface of the sirtuin and it also includes residues in the cavity lining the ac(et)yl-lysine binding pocket leading into the active site. The NAD^+^-binding site in the Rossmann-fold domain is inverted compared to other NAD^+^-binding domains. The N-terminal part of the β-sheet binds to the nicotinamide moiety and the C-terminal part to the adenine base of the NAD^+^. The NAD^+^-binding site in sirtuins is subdivided into three sites: the A site binds the adenine-ribose, the B-site the nicotinamide-ribose and the C-site the nicotinamide moiety of the NAD^+^ (Sanders et al., 2010). The NAD^+^ phosphates are bound by an invariant Gly-X-Gly motif that is conserved in mammalian and bacterial sirtuins.

A strategy for development of selective and potent sirtuin inhibitors is the development of peptide-based mechanistic inhibitors applying acetyl-lysine analogs such as trifluoroacetyl-lysine and thioacetyl-lysine embedded into a protein/peptide substrate sequence (Smith and Denu, 2007b; Smith et al., 2008; Knyphausen et al., 2016a; Kuhlmann et al., 2017). These analogs have a strong electron withdrawing potential resulting in a strong reduction in the nucleophilicity of the carbonyl oxygen of the acetyl-group. As a consequence, these analogs result in a by several orders of magnitude reduced sirtuin-catalyzed deacetylation rate (Smith and Denu, 2007a, b). Application of these mechanism-based inhibitors on bacterial sirtuins might constitute a novel strategy for therapeutic interventions.

For the mammalian sirtuins the catalytic mechanism is still under debate. Experimental results support either an S_N_1, a concerted S_N_2 or a dissociative S_N_2-like mechanism for lysine diacylation (Sauve, 2010; Feldman et al., 2012). As bacterial sirtuins are structurally and based on their primary sequence very similar to the mammalian sirtuins, a similar catalytic mechanism is likely. In fact, it can be assumed that the mammalian sirtuins evolutionary originate from the bacterial enzymes. It is believed that the mitochondrial sirtuins derived from the bacterial enzymes and the mammalian sirtuins in the nucleus and or cytosol developed subsequently with the translocation of genomic information from the mitochondria to the nucleus (Frye, 2000). During catalysis of lysine deacylation the carbonyl oxygen of the substrate acyl-lysine performs nucleophilic attack on the electrophilic C-1′ of the ribose of NAD^+^ (Figure 4B and Supplementary Figure 2). This results in fast release of nicotinamide, which can be used as non-competitive sirtuin inhibitor with an reported inhibition constant, K_*i*_, in the range of 50–100 μM (Gallego-Jara et al., 2017). Intracellular concentrations of nicotinamide were reported to be in the same range (30–70 μM) in *E. coli* during exponential growth suggesting that nicotinamide plays a regulatory role for CobB activity *in vivo* (Gallego-Jara et al., 2017). A C-1′-*O*-alkylamidate intermediate is formed that collapses by attack of the C-2′ hydroxyl of the NAD^+^ ribose on the *O*-alkylamidate carbon. This results in formation of a cyclic 1′,2′-intermediate, which is resolved by attack of an active site water molecule, which is activated by a histidine acting as general base (*E. coli* CobB: His147; *B. subtilis* SrtN: His118; Figure 4B). This attack results in formation of the reaction products 2′-*O*-ac(et)yl-ADP-ribose and the deacylated lysine side chain. The 2′-*O*-ac(et)yl-ADP-ribose is exists in a non-enzymatic equilibrium with 3′-*O*-acetyl-ADP-ribose. The formed *O*-acetyl-ADP-ribose was to elicit biological responses in eukaryotes and ADP-ribose acts as second messenger in mammalian cells (Supplementary Figure 2; Perraud et al., 2001; Borra et al., 2002). Future studies are needed to uncover the physiological role of *O*-acetyl-ADP-ribose and/or ADP-ribose in bacteria.

## Acetyltransferases and Deacetylases Are Sensors of the Metabolic State

All acetyltransferases use ac(et)yl-CoA as the donor molecule for the ac(et)ylation of the N-terminal amino group of proteins, of lysine side chains in proteins and of small molecules including aminoglycosides, vitamins, and polyamines such as spermine, cadaverine, spermidine, and putrescin (Figure 1D; Vetting et al., 2005; Favrot et al., 2016; Shirmast et al., 2021). Sirtuins use NAD^+^ as co-substrate to remove the ac(et)yl-group from lysine side chains. The following section gives a comprehensive summary how these enzymes sense the metabolic state to translate this directly into altered protein activities by affecting the lysine acetylation status.

### Bacterial Protein Acetyltransferases Sense the Cellular Acetyl-CoA Levels

**FIGURE 5:**
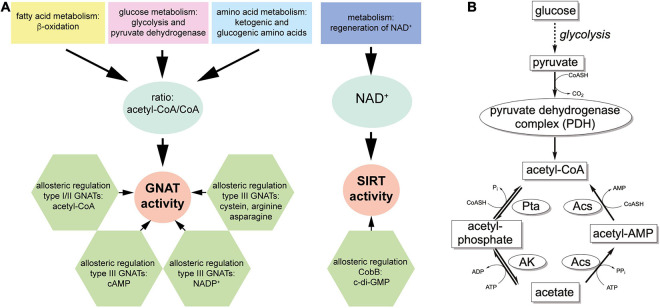
Ac(et)ylation is a modification of molecules that is tightly connected to the cellular metabolic state. **(A)** Left panel: GNATs depend on ac(et)yl-CoA as donor molecule for the transfer of the ac(et)yl group to the substrate amino group. Shown is only acetyl-CoA but dependent on the specificity of the GNATs for different acyl-CoAs, also other acyl groups can be transferred. Acetyl-CoA is generated in the metabolism of all major nutrient classes. Dependent on the metabolic state, the intracellular concentration of ac(et)yl-CoA fluctuates. The catalytic efficiency of the GNATs depend on the intracellular acetyl-CoA/CoA ratio rather than the acetyl-CoA concentration. This is reflected by the similar K_M_-values of GNATs for ac(et)yl-CoA and CoA. Moreover, several GNATs are regulated by binding of metabolic molecules such as cAMP, NADP^+^, acetyl-CoA and amino acids to accessory, allosteric sites. Binding of the ligands to the allosteric site modulates GNAT activity constituting another layer for regulation of GNAT activity. Further regulatory systems include post-translational modifications of GNATs, not shown here. Right panel: sirtuin activity is also tightly connected to the cellular metabolic state. Sirtuins use NAD^+^ as stoichiometric co-substrate for catalysis and other endogenous regulators such as c-di-GMP for CobB also exist. As described for GNATs, also sirtuins are regulated by post-translational modifications which are not shown here. **(B)** Acetyl-phosphate is the major factor for non-enzymatic acetylation in bacteria. Acetyl-CoA is formed during metabolism of all major nutrient classes. Shown is the formation of acetyl-CoA through glycolysis starting from glucose to form pyruvate, which is oxidatively decarboxylated to form acetyl-CoA by the pyruvate dehydrogenase complex (PDH). Acetyl-CoA can subsequently further metabolized either by oxidative phosphorylation under aerobic conditions to drive formation of ATP and regeneration of NAD^+^ or it is converted to acetate by phosphotransacetylase (Pta) catalyzing the formation of acetyl-phosphate and acetate kinase (AK) yielding acetate. Both reactions are reversible, i.e., AK and Pta can also convert acetate to acetyl-CoA under consumption of ATP. Alternatively, the acetate can be converted by acetyl-CoA synthetase (Acs) to form acetyl-CoA. Due to the production of pyrophosphate (PP_i_) in the first half reaction yielding acetyl-adenylate (acetyl-AMP), this reaction is almost irreversible. Under conditions of carbon overflow, the intracellular acetate concentration (>5 mM) increases so that also the reaction catalyzed by AK and Pta to produce acetyl-CoA from acetate becomes important. This results in accumulation of acetyl-phosphate under conditions of carbon overflow causing systemic non-enzymatic acetylation.

Acetyl-CoA is the central metabolite and a second messenger, integrating all main metabolic pathways involved in carbohydrate, fatty acid and protein metabolism (Figure 5A). Acetyl-CoA is produced under aerobic conditions in carbohydrate metabolism by oxidation of glucose through glycolysis and subsequent oxidative carboxylation of pyruvate by the pyruvate dehydrogenase (PDH) complex. Under anaerobic conditions bacteria can also convert pyruvate to acetyl-CoA by pyruvate-formate lyase (PFL) or by pyruvate-ferredoxin-oxidoreductase (PFO). Moreover, acetyl-CoA is formed in β-oxidation of fatty acids and by decomposition of ketogenic amino acids either directly (Leu, Ile, Trp) or indirectly *via* formation of acetoacetyl-CoA and subsequent conversion to acetyl-CoA by thiolase (Leu, Lys, Phe, Trp, Tyr). Most amino acids are glucogenic and can be converted to glucose *via* gluconeogenesis, which can be metabolized to indirectly yield acetyl-CoA (Figures 5A,B; Krivoruchko et al., 2015). The amount of product formed can only be modified by altering the concentrations of the reaction partners or the products, while the equilibrium of the reaction is not affected. Acetyl-CoA can also be produced by acetyl-CoA synthetase (Acs) in an irreversible ATP-consuming reaction that is composed of two half reactions: (1) formation of acetyl-adenylate (acetyl-AMP) from acetate and ATP, (2) reaction of acetyl-AMP with coenzyme A (CoASH) to form acetyl-CoA and AMP (Figure 5B; Starai et al., 2002; Wolfe, 2005; Krivoruchko et al., 2015). Next to this Acs a second acetyl-CoA synthetase is reported in some archaea such as *Pyrococcus furiosus* and eukaryotic protists such as *Entamoeba histolytica* that coverts acetate and CoASH in a single step to acetyl-CoA (Reeves et al., 1977). A reversible reaction to produce acetyl-CoA is *via* conversion of acetate and phosphate derived from ATP to yield the high-energy metabolite acetyl-phosphate (AP) and ADP by acetate kinase (AK) and conversion of AP to acetyl-CoA by phosphotransacetylase (Pta) (Figure 5B). Notably, this pathway is important for acetyl-CoA production in cells under conditions of carbon overflow in which high cellular acetate concentrations (>5 mM) are accumulating (Krivoruchko et al., 2015; Schilling et al., 2015). In contrast, under conditions of low acetate concentrations (~0.2 mM) acetyl-CoA synthetase (Acs *E. coli*: K_M_ 0.2 mM; Acs *S. enterica*: 6 mM; Acs *M. tuberculosis*: 1.2 mM) produces acetyl-CoA for anabolic reactions (Figure 5B; Brown et al., 1977; Kumari et al., 1995, 2000; Reger et al., 2007; Li et al., 2011). Notably, it is surprising that the K_M_-values toward acetate reported for Acs of different bacterial species vary strongly considering the high degree of sequence identity *E. coli*: 0.2 mM; *S. enterica*: 6 mM; *M. tuberculosis*: 1.2 mM) (Brown et al., 1977; Reger et al., 2007; Li et al., 2011). Finally, acetyl-CoA is produced by ATP-citrate lyase in reductive tricarboxylic acid cycle (reverse TCA cycle) that is used by some autotrophic bacteria such as *Chlorobium* species for carbon dioxide assimilation (Wahlund and Tabita, 1997; Kanao et al., 2001, 2002; Hugler and Sievert, 2011). In eukaryotes ATP-citrate lyase catalyzes the conversion of citrate, that is transported from the mitochondrial matrix into the cytosol, and CoASH to form oxaloacetate and acetyl-CoA in the cytosol (Wei et al., 2020). Besides from these main routes of acetyl-CoA production, other specific routes were described in specific bacterial strains. As an example, an alternative acetate-driven TCA cycle was shown to exist in diverse bacterial species that live as symbionts in animals and insects. In this cycle the acetate-succinate-CoA transferase (ASCT) replaces succinyl-CoA synthetase resulting in formation of acetyl-CoA and succinate from succinyl-CoA and acetate (Kwong et al., 2017).

### Origins of Other Acyl-CoA Species in Bacteria for Enzymatic and Non-enzymatic Acylation

In mammals, KATs were shown to use different acyl-CoA molecules as donors for protein acetylation, such as propionyl-CoA, butyryl-CoA, malonyl-CoA, hydroxyisobutyryl-CoA, and crotonyl-CoA (Figure 1C). Next to acetyl-CoA, GNATs in eukaryotes and prokaryotes were reported to also catalyze acylation using different acyl-CoA molecules as donor molecules for acyl group transfer such as butyryl-CoA and propionyl-CoA (Figure 1C). As an example, *Se*Pat uses acetyl-CoA to acetylate acetyl-CoA synthetase (Acs) and propionyl-CoA to propionylate propionyl-CoA synthetase (PrpE). In both cases acylation in the C-terminus of acyl-CoA synthetase results in its inactivation (Garrity et al., 2007; Takenoya et al., 2010). Recently, crotonylation was identified as widespread acylation in *Streptomyces roseosporus* (Sun et al., 2020). The GNAT acyl-transferase Kct1 from *S. roseosporus* was identified as efficient crotonyl-transferase for the glucose kinase converting glucose to glucose 6-phosphate resulting in inhibition of its catalytic activity (Sun et al., 2020).

So far bacterial protein acetyltransferases were not systematically analyzed concerning their acyl-chain preferences and future studies are needed to elucidate the activities and specificities for diverse bacterial acetyltransferases. These data will unravel the physiological roles of the respective GNATs and will furthermore show how substrate specificity is created for the diverse GNAT types. In bacteria diverse other acyl-CoA molecules apart from acetyl-CoA are produced in metabolism (Figure 1C). Malonyl-CoA can be produced from acetyl-CoA by carboxylation and under consumption of ATP by the biotin-dependent enzyme acetyl-CoA carboxylase (Tong, 2005; Polyak et al., 2012). Propionyl-CoA is produced upon degradation of branched chain amino acids (Ile, Thr, Val) and Met, odd-chain fatty acids and by the oxidation of the side chain of cholesterol (Kazakov et al., 2009; Wilburn et al., 2018). Propionyl-CoA can be converted to succinyl-CoA by the enzymes propionyl-CoA carboxylase (PCC), methylmalonyl-CoA epimerase (MCEE), and methylmalonyl-CoA mutase (MCM) (Haller et al., 2000). Succinyl-CoA is also an intermediate of the tricarboxylic acid (TCA)-cycle formed by oxidative decarboxylation of α-ketoglutarate by α-ketoglutarate dehydrogenase (KDH) (Thomas, 1974; Wolodko et al., 1986). Moreover, in many bacteria succinyl-CoA is formed in the reductive TCA cycle from succinate by succinyl-CoA synthetase (succinate-CoA ligase) in an ATP/GTP-dependent reaction (Jenkins and Weitzman, 1986). Crotonyl-CoA can be produced by acyl-CoA synthetase from the short-chain fatty acid crotonate. Moreover, crotonyl-CoA is formed during fermentation of butyrate, in fatty acid synthesis from β-hydroxybutyryl-CoA and also during degradation of the amino acids lysine and tryptophan. Some bacteria use the ethylmalonyl-CoA pathway for the assimilation of acetate. During the course of this pathway crotonyl-CoA is formed by condensation of two molecules acetyl-CoA catalyzed by β-ketothiolase and subsequent NADH-dependent reduction of the reaction product acetoacetyl-CoA to crotonyl-CoA by acetoacetyl-CoA reductase (Erb et al., 2009; Schink, 2009).

### Intracellular Prevalence of Acyl-CoA Molecules in Bacteria

The presence and intracellular concentrations of these acyl-CoA molecules are direct indicators for the cellular metabolic state. A systematic evaluation of cellular concentrations of various acyl-CoAs in different growth phases and under different physiological conditions has not been performed in bacteria so far. This in combination with a thorough biochemical characterization of the enzymes is needed to judge the physiological importance of GNAT-catalyzed acyl-transfer. Some data on concentrations of acyl-CoA and CoA in bacteria is available. In *E. coli*, the concentrations of acetyl-CoA and malonyl-CoA were reported to fluctuate between 200–600 μM and 4–90 μM, respectively (Chohnan et al., 1997, 1998; Hou et al., 2015). Acetyl-CoA concentrations were highest during exponential growth phase and they declined during stationary growth phase. This shows that the acetyl-CoA level directly follows the available glucose levels. If other carbon sources are used, such as acetate and glycerol, the overall cellular acyl-CoA levels were strongly reduced (Takamura and Nomura, 1988). For the bacterial GNAT acetyltransferases K_M_ values of the GNAT domain for acetyl-CoA were reported that are in the micromolar range suggesting that the GNAT activities can directly and precisely be adjusted to alterations and availability of cellular acetyl-CoA concentrations and/or the cellular GNAT protein level (Nambi et al., 2010; Xu et al., 2014).

Studies in *E. coli* grown on different carbon sources showed that the overall acetyl-CoA level always exceeded the cellular malonyl-CoA levels. Furthermore, acetyl-CoA production increased strongly using various monosaccharides as carbon source such as D-glucose, D-mannose and D-fructose, while almost no acetyl-CoA production was induced using either succinate or acetate as carbon source. Under these conditions the cellular concentration of CoA exceeded the concentrations of acetyl-CoA/malonyl-CoA approximately 2-fold/20-fold (Takamura and Nomura, 1988; Chohnan et al., 1997, 1998). This ensures that the GNAT enzymes are not strongly active under these conditions. For the mammalian KATs, it was reported that the acetyl-CoA/CoA ratio is an important regulatory mechanism as affinities of KATs for acetyl-CoA and CoA are in the same order of magnitude. This suggests a possibility for a feedback regulatory mechanism of KAT activity by product inhibition due to increase in CoA concentration (Denisov and Sligar, 2012; Henry et al., 2015; Pietrocola et al., 2015). For exponentially growing *E. coli* cells intracellular concentrations of acetyl-CoA and CoA of 0.41 and 1.4 mM were reported (Bennett et al., 2009). This fluctuates dependent on the metabolic state and the nutrient availability. A similar regulatory mechanism likely exists for the activity of bacterial GNATs by the intracellular acetyl-CoA/CoA ratio rather than the concentration of acetyl-CoA alone.

Intracellular concentrations of propionyl-CoA were shown to be in the range of 40–200 μM in *Haloferax mediterranii*, but this might fluctuate dependent on the bacterial species and the physiological state (Hou et al., 2015).

It was shown that that under conditions in which intracellular acetyl-CoA levels are diminished, lysine succinylation becomes more prevalent showing that the availability of various acyl-CoA types can affect overall acylation with diverse acylations (Kosono et al., 2015; Mizuno et al., 2016). All acyl-CoA molecules are reactive thioesters that have the potential to modify lysine side chains either non-enzymatically or maybe also enzymatically. This needs further future investigation how diverse lysine acylations regulate protein function and how these protein acylations are regulated in bacteria.

For many mammalian KATs it was shown that the activities decreased with acyl-CoA chain length (Simithy et al., 2017). This means that acetyl-CoA is the preferred donor molecule for acetylation if it is present at least in equimolar concentrations in cells. If this is true also for the bacterial enzymes or if some bacterial GNAT enzymes have been evolved toward preferences for longer acyl-chain lengths needs to be evaluated in the future.

### Bacterial GNAT-Related Protein Acetyltransferases Are Allosterically Regulated by Acetyl-CoA/CoA, cAMP, Amino Acids, and NADP^+^

Several bacterial GNATs are regulated by binding of ligands such as acetyl-CoA, cAMP, amino acids and NADP^+^ to regulatory domains N- or C-terminal to the catalytic GNAT domain. as reported above. All of these ligands are themselves direct indicators for the cellular physiological and metabolic state (Figure 5A).

GNATs of type I and II use their N- or C-terminal NDP-forming acyl-CoA synthetase domain as a sensor for cellular ac(et)yl-CoA (Thao and Escalante-Semerena, 2012; de Diego Puente et al., 2015). For *S. enterica Se*Pat it was shown that it binds two molecules of ac(et)yl-CoA with the N-terminal regulatory and the C-terminal GNAT domain, respectively. Mutational analyses revealed that the N-terminal domain is essential for the catalytic activity in *E. coli* PatZ and *Se*Pat (Thao and Escalante-Semerena, 2011; de Diego Puente et al., 2015). For *Se*Pat it was shown that the N-terminal domain binds ac(et)yl-CoA with a nanomolar affinity (K_D_: 290 nM), while binding to the GNAT domain was approximately one order of magnitude lower (K_D_: 2.4 μM). This shows that the regulatory NDP-forming acyl-CoA synthetase domain in type I and type II GNATs might reflect a metabolic sensory domain that allows a tight control of the enzymatic activity avoiding a background activity under conditions of low cellular acetyl-CoA concentrations.

GNATs of type III are able to sense amino acids cysteine, arginine and asparagine, *via* their N-terminal ACT domains or cellular cAMP levels *via* their N-terminal cAMP-binding domains (Supplementary Figure 1A; Nambi et al., 2010; Shimada et al., 2011; Xu et al., 2011, 2014; Gancedo, 2013). Binding of these ligands was shown to stimulate their GNAT activity *via* an allosteric mechanism (Nambi et al., 2010; Xu et al., 2014). For the ACT-domain containing ACT-GNAT *Ma*Kat from *Micromonospora aurantiaca* (Micau_1670) affinities toward Cys and Arg were reported in the micromolar range (80–210 μM) similar to their reported intracellular cytosolic concentrations (*E. coli* 100–200 μM) (Park and Imlay, 2003; Caldara et al., 2008; Xu et al., 2014). The cyclic nucleotide cAMP is an important second messenger in mammalian cells and in bacteria. It is formed by adenylyl cyclase and can directly mediate signal transduction processes. In bacteria cAMP is generated by adenylate cyclase if cellular glucose uptake by the phosphotransferase system declines (Supplementary Figure 2). If the cell is energy deprived, cAMP accumulates in the bacterial cytosol (Saier, 1996a, b; Saier and Crasnier, 1996; Saier and Ramseier, 1996; Ye and Saier, 1996; Lin et al., 2004). For *M. smegmatis* intracellular concentrations of cAMP in the range of 100 μM–1 mM were reported (Dass et al., 2008). In glucose-fed *E. coli* cells the cAMP concentration is 35 μM, which increased to 146 μM with growth on acetate as carbon source (Bennett et al., 2009). The affinity of the cAMP-binding domain of the *M. smegmatis* cAMP-GNAT MSMEG_5458 was reported to be in the range of 100 nM suggesting complete saturation under these intracellular concentrations (Nambi et al., 2010; Lee et al., 2012). While binding of cAMP to MSMEG_5458 only resulted in a moderate, 3-fold increase in the GNAT activity for the *M. tuberculosis* cAMP-GNAT enzyme Rv0998 a strong increase in the acetyltransferase activity was shown upon cAMP-binding (Nambi et al., 2010, 2013). This cAMP accumulation results in activation of metabolic routes that allow utilization of alternative carbon sources (Shimada et al., 2011; Gancedo, 2013). Notably, under persistent conditions of glucose deprivation and starvation under which also the cellular ATP levels decrease drastically a decline of the cellular cAMP levels is observed as adenylyl cyclase uses ATP for production of cAMP (Nambi et al., 2010). As an example cAMP is used in bacteria to bind and to activate an important transcription factor, CAP/CRP, which modulates the expression of more than 100 genes (*E. coli* K12: 378 genes) (Bruckner and Titgemeyer, 2002; Shimada et al., 2011; Molina-Quiroz et al., 2018). Binding of cAMP to CAP/CRP induces a conformational change that allows to bind to the target DNA promotor sites which in turn recruits the RNA polymerase holoenzyme to initiate or repress transcription of target genes. CAP/CRP is a major regulator for metabolic adaptation to various carbon sources in bacteria. In *E. coli* it was shown that during growth on glucose upon entry into the stationary growth phase the transcription of the acetyltransferase *pat* gene was initiated by binding of cAMP to CAP/CRP. Alternatively, growth on acetate induces *pat*-expression in *E. coli via* the cAMP-CAP route. Generally, cAMP is the major regulator for regulation of carbon flux in glucose limited cultures in *E. coli*.

In contrast, some GNATs, such as cAMP-GNAT *Mt*Pat (Rv0998), are shown to be directly regulated allosterically by cAMP on the post-translational level. In both cases, activation of GNAT activity by rise in the cellular cAMP level results in inactivation of acetyl-CoA synthetase (Acs) activity through acetylation of a lysine residue in the C-terminus (*E. coli* K609. *M. tuberculosis*: K617, *S. enterica*: K609). Furthermore, for *Salmonella enterica* acetyl-CoA synthetase *Se*Acs it was shown that cAMP can act as direct competitive inhibitor by binding to its ATP/AMP pocket (Han X. et al., 2017). To this end, cAMP impairs Acs activity by applying three mechanisms: *acs* activity is reduced by inducing *gnat* expression *via* activation of cAMP-CAP (transcriptional regulation), directly by binding to and allosteric activation of cAMP-GNAT activity resulting to Acs acetylation (post-translational regulation) and its inactivation and by directly binding to the active site and competitively inhibiting Acs. These three mechanisms ultimately result in inhibition of Acs activity and therefore to a reduced cellular acetyl-CoA level. While for *E. coli* Acs, the K_M_ value toward acetate is reported to be 0.2 mM, the K_M_ value of *Se*Acs for acetate is in the millimolar range suggesting that Acs activity is important for generation of acetyl-CoA under conditions of high cellular acetate concentrations and low cAMP levels generated if glucose deprivation persists and cellular ATP level declines (Brown et al., 1977; Reger et al., 2007; Han X. et al., 2017).

Type III GNAT Mxan_3215 (*Mx*PAT) was shown to be negatively regulated by binding to the coenzyme NADP^+^ (Supplementary Figures 1B, 2; Liu et al., 2015). The affinity of *Mx*PAT to NADP^+^ was shown to be 2.9 μM in the same range of intracellular concentrations reported for *E. coli* (0.14–31.1 μM) suggesting that the activity of the NADP^+^-GNAT can be modified under physiological conditions by fluctuations in the intracellular NADP^+^ concentration (Bennett et al., 2009; Liu et al., 2015).

The type V GNATs Eis and Eis2 from *M. tuberculosis* and *M. abscessus*, respectively, were structurally characterized (Chen W. et al., 2011; Kim et al., 2012; Ung et al., 2019). *Mycobacterium* Eis GNATs were shown to act as aminoglycoside acetyltransferases and protein acetyltransferases acetylating proteins needed for host cell infection (Kim et al., 2012; Kumar et al., 2012; Houghton et al., 2013; Ung et al., 2019). The structures revealed that only the N-terminal GNAT domain crystallized with CoA or acetyl-CoA suggesting that either the affinity of the central GNAT domain for CoA and acetyl-CoA is very low or it is in an incompetent conformation for binding (Supplementary Figure 2; Chen W. et al., 2011; Houghton et al., 2013; Ung et al., 2019). To this end, most likely binding of acetyl-CoA/CoA to the central domain in Eis does not play a regulatory role under physiological conditions.

### Bacterial Sirtuins Sense the Cellular NAD^+^ Level and Can Be Regulated by c-di-GMP

All sirtuins use an NAD^+^-dependent catalytic activity to either catalyze lysine deacylation or weak ADP-ribosylation (Supplementary Figure 2 and Figure 5A). The K_M_ values of sirtuins for NAD^+^ were reported to reside in the micromolar range (~30–900 μM) (Hirschey et al., 2011; Feldman et al., 2015; Olesen et al., 2018). For mammalian sirtuins the affinities for NAD^+^ were shown to depend on presence of the substrate, the identity of substrate and on the substrate concentration (Fischer et al., 2012). It is likely that a similar influence can also be observed for bacterial sirtuins and that K_M_-values/affinities for NAD^+^ are similar in eukaryotic and prokaryotic sirtuins. The observed NAD^+^ K_M_-values/affinities are within the range of intracellular NAD^+^ concentrations (*E. coli*: up to 2.6 mM; *L. acidophilus*: 0.2–5 mM) suggesting that the activities of bacterial enzymes are regulated by the availability of intracellular NAD^+^ (Bennett et al., 2009; Olesen et al., 2018). For the mammalian sirtuins, binding to NADH was shown to be substantially weaker and the intracellular concentrations of free NADH are much lower compared to NAD^+^ (Schmidt et al., 2004; Cambronne et al., 2016; Madsen et al., 2016; Anderson et al., 2017b). This was also confirmed in bacteria. *E. coli* and *L. acidophilus* showed an intracellular NADH concentration of 83 μM or it was almost undetectable, respectively (Bennett et al., 2009; Olesen et al., 2018). In analogy to the mammalian sirtuins, this suggests that the activity of bacterial sirtuins is regulated by the cellular NAD^+^ concentration rather than the NAD^+^/NADH ratio (Figure 5A; Schmidt et al., 2004; Madsen et al., 2016; Anderson et al., 2017b).

Mammalian sirtuins are mostly composed of the catalytic domain and additional N- and C-terminal extensions of various lengths. These additional regions were shown to affect sirtuin deacylase activity as autoregulatory domains, as binding sites for allosteric regulators such as small molecules or nucleic acids, as regions important for their subcellular localization or as regions of post-translational modifications regulating catalytic activity (North and Verdin, 2007; Han et al., 2008; Pandithage et al., 2008; Li et al., 2015; Tong et al., 2016, 2017; Huang et al., 2018; Shang et al., 2020). Most bacterial sirtuins do not contain extensive regions apart from their catalytic domain. However, CobB was shown to exist in two isoforms, a long (CobB_L_) and an N-terminally by 37 residues shorter short isoform (CobB_S_) (Tucker and Escalante-Semerena, 2010; Umehara et al., 2018). CobB_L_ was shown to be N-terminally acetylated by the acetyltransferase YiaC/NatA impairing CobB_L_’s catalytic activity, shown to have a dual activity acetylating N-α- and N-ε-amino groups in proteins (Parks and Escalante-Semerena, 2020). The N-terminal region was shown to be important for binding to the intracellular signaling molecule c-di-GMP (Figure 5A and Supplementary Figure 2). Binding of c-di-GMP to CobB_L_ resulted in increased acetylation of known CobB substrates such as acetyl-CoA synthetase, CheY and NhoA (Xu et al., 2019). Binding constants were in the low micromolar range (K_D_: 4.7 μM), which is in in the same range as intracellular c-diGMP concentrations in bacteria in the sub- to low micromolar range (Simm et al., 2004; Romling et al., 2013; Xu et al., 2019). Again, this shows that enzymatic activity can be modulated by dynamic changes in the cellular concentration of the regulator under physiological conditions.

## Classical Deacetylases Are Zn^2+^-Dependent Enzymes

**FIGURE 6:**
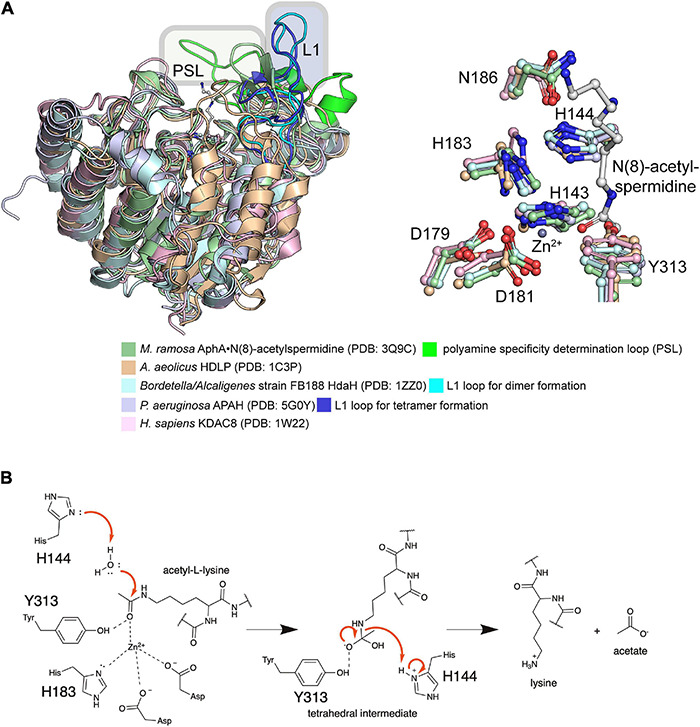
Bacterial Zn^2+^-dependent classical deacetylases. **(A)** Structural analyses of bacterial classical deacetylases. Left panel: several bacterial classical deacetylases were structurally characterized. The enzyme AphA from *M. ramosa* encodes for a acetyl-polyamine amidohydrolase (AphA) that was categorized into class II. *A. aeolicus* histone deacetylase-like protein (HDLP) was classified to class I and *Bordetella*/*Alcaligenes* strain FB188 histone deacetylase-like amidohydrolase (HdaH) to class IIb. Both enzymes are active as protein deacetylases. The class IIb enzyme *P. aeruginosa* APAH (PA3774) was shown to be inactive toward polyamines and suggested to act as protein deacetylase. For comparison the structures were superimposed to the structure of class I KDAC8 from human. Notably, the L1 loop in bacterial classical deacetylases is a structural feature needed for oligomerization of and catalytic activity and the polyamine specificity determination loop (PSL) conveys substrate specificity toward polyamines. Right panel: The active site is totally conserved in the enzymes compared. The only difference is the residue N186 in *P. aeruginosa* APAH PA3774 (and *M. ramosa* AphA, *Bordetella*/*Alcaligenes* strain FB188 HdaH), which is a aspartate in KDAC8 (D183), and *A. aeolicus* HDLP (D173). Functionally, both asparagine and aspartate contact, orient and polarize the general base histidine (*P. aeruginosa* APAH PA3774 H144) [the figure was generated with PyMOL v.2.3.4 (Schrödinger LLC, 2000)]. **(B)** Catalytic mechanism exerted by bacterial classical deacetylases. As the active site is totally conserved between mammalian and bacterial classical deacetylases, a similar catalytic mechanisms can be assumed. Classical deacetylases use a catalytic Zn^2+^-ion that is coordinated by an two Asp, one His, one Tyr and the ac(et)yl-moiety of the substrate amino group, i.e., derived from a polyamine of a protein lysine side chain. The Zn^2+^-ion contacts the carbonyl oxygen thereby polarizing the carbonyl group of the acetyl-group. This results in an increase in the electrophilicity at the carbonyl C-atom enabling nucleophilic attack of an active site water molecule. This water molecule is activated by the histidine residue (human KDAC8: H143; *P. aeruginosa* APAH PA3774: H144). This histidine acts as general base abstracting a proton from the water thereby increasing its nucleophilicity. A second histidine (human KDAC8: H142; *P. aeruginosa* APAH PA3774: H143) fulfills the role of electrostatic catalyst. Both histidine residues are integral parts of Asp-His charge-relay systems. A tetrahedral oxyanion intermediate is build that is stabilized by a tyrosine (human KDAC8: Y306; *P. aeruginosa* APAH PA3774: Y313). This is resolved by the catalytic histidine (human KDAC8: H143; *P. aeruginosa* APAH PA3774: H144) acting as proton donor (catalytic acid) to the substrate amino group finally resulting in release of acetate and the deacetylated amino group [figure is redrawn and modified from Ali et al. (2018) and Blasl et al. (2021)].

Humans encode for 11 classical, Zn^2+^-dependent lysine deacetylases. As these enzymes were originally found to be important as histone deacetylases they were originally called histone deacetylases (HDACs). However, recent data obtained with quantitative mass-spectrometry revealed that these enzymes have various non-histone substrates in several cellular compartments, the denomination as lysine deacetylases (KDACs) is more suitable. KDACs are metalloenzymes, structurally different from sirtuins and they exert a different catalytic strategy involving a catalytic Zn^2+^-ion to achieve deacylation of substrate lysine side chains. Also in bacteria, classical Zn^2+^-dependent deacetylases were discovered, which were structurally and with respect to the active site organization highly similar to the mammalian enzymes (Figures 6A,B).

### Classification of Classical Deacetylases

Based on sequence, structure and function mammalian KDACs are classified into the classes I, II, and IV, while sirtuins build class III (Blasl et al., 2021). All mammalian KDACs contain a conserved catalytic domain of approximately 300–400 amino acids. Only KDAC6 contains two catalytic domains. Mammalian KDACs of class I (nuclear localization) are related to *S. cerevisiae* Rpd3 (reduced potassium dependency 3) and encompass KDAC1, 2, 3, and 8. Class II enzymes are related to yeast Hda1 (histone deacetylase 1). Class II is subdivided in subclass IIa including KDAC4, 5, 7, and 9 and subclass IIb with KDAC6 and KDAC10. Class IIb enzymes contain an N-terminal extension preceding the catalytic domain. For all members of class II it was shown that they shuttle between the nucleus and the cytosol. Class IV contains only a single enzyme, KDAC11, which was shown to localize to the nucleus. This classification was done based on structure, function and catalytic mechanism (de Ruijter et al., 2003). Recent mass-spectrometric data showed the presence of thousands of acetylation sites in all kingdoms of life, in proteins covering all essential cellular functions and in all cellular compartments (Choudhary et al., 2009, 2014; Lundby et al., 2012; Hansen et al., 2019). Mammalian KDACs are present in multiprotein-repressor complexes that determine their substrate specificity and the enzymatic activity. Several potent and selective mammalian KDAC-inhibitors are in clinical trials or are approved by the FDA (Food and Drug Administration) (Nebbioso et al., 2017; Lopez et al., 2018; Beyer et al., 2019). KDAC-inhibitors were shown to be neuroprotective in models of neurodegeneration (Ziemka-Nalecz et al., 2018). Classical KDAC inhibitors can be classified according to their chemical structures in hydroxamates, such as trichostatin A (TSA) or SAHA (vorinostat), short-chain fatty acids, such as butyrate, cyclic peptides or benzamides, such as MS-275 (etinostat) (Supplementary Figure 2; Yoshida et al., 2017; Zhang et al., 2018). Compounds were developed for classical KDACs that are very potent and selective with IC_50_ values in the low nanomolar range (Li and Seto, 2016; Pasyukova and Vaiserman, 2017; Biran et al., 2018; Bourguet et al., 2018; Li et al., 2018; McIntyre et al., 2019). Hydroxamates, such as suberoylanilide hydroxamic acid (SAHA) and trichostatin A are pan inhibitors inhibiting almost all classical KDACs to a similar extend (Supplementary Figure 2). These inhibitors chelate the active-site Zn^2+^-ion, and replace the active site water molecule needed for nucleophilic attack of the electrophilic carbonyl carbon of the acetyl-group (Lombardi et al., 2011b; Yoshida et al., 2017; Zhang et al., 2018). Future studies are needed to show if bacterial KDACs are also inhibited using the inhibitors developed for mammalian enzymes or if these can be used in a drug-repurposing strategy.

### Catalytic Mechanism of Bacterial Classical Deacetylases

During catalysis of classical KDACs a water molecule is activated by two histidine residues of a conserved tandem His-motif. One histidine (human KDAC8: H143; *P. aeruginosa* APAH: H144) acts as general base abstracting a proton from the catalytic water molecule thereby increasing its nucleophilicity for attack at the electrophilic carbonyl carbon of the acetyl-group (Figure 6B). The second histidine residue (human KDAC8: H142; *P. aeruginosa* APAH: H143) is important to orient and polarize the catalytic water molecule acting as electrostatic catalyst. Both histidine residues are polarized and oriented by two aspartate residues/one asparagine and one aspartate, respectively (human KDAC8: D183, D176; *P. aeruginosa* APAH: N186, D179) (Figure 6B). Another histidine is involved in coordination of the active site Zn^2+^-ion (human KDAC8: H180; *P. aeruginosa* APAH: H183). The active site Zn^2+^-ion coordinates and polarizes the attacking water molecule (Lombardi et al., 2011b). Furthermore, the Zn^2+^-ion, together with a Tyr (human KDAC8: Y306; *P. aeruginosa* APAH: Y313), which is replaced by a His in class IIa enzymes, increases the electrophilicity of the carbonyl carbon of the acetyl-group by contacting the carbonyl oxygen. During catalysis the Zn^2+^-ion and the Tyr stabilize the tetrahedral oxyanion intermediate. This is resolved by proton transfer of the histidine that initially acted as general base, now fulfilling the role as catalytic acid (Figure 6B). Specificity of the inhibitors is furthermore created by approaching sequence and structural differences between the KDAC-isoforms (Marmorstein, 2001; Marmorstein and Zhou, 2014; Li and Sun, 2019).

### Prokaryotic Zn^2+^-Dependent Classical Deacetylases Act as Polyamine Deacetylases and Protein Lysine Deacetylases

The knowledge on classical deacetylases in prokaryotes is limited and only a few classical deacetylases were identified and characterized in bacteria so far. In *E. coli*, no classical deacetylase is encoded. The Zn^2+^-dependent acetylpolyamine amidohydrolase AphA from *Mycoplana ramosa* was amongst the first classical KDACs identified (Sakurada et al., 1996). Activity studies revealed that *M. ramosa* AphA is effective on deacetylation of polyamines such as acetyl-spermidine, acetyl-spermine or acetyl-putrescin (Figure 1D). *M. ramosa* was shown to grow on medium with spermidine or putrescine as sole carbon source suggesting that AphA plays an important role during growth on mono- or diacetylated polyamines (Sakurada et al., 1996). Later structural data showed that *M. ramosa* AphA forms a dimer and that dimer formation is important for its catalytic activity. Moreover, a hydroxamate inhibitor was potent to inhibit *M. ramosa* catalytic activity showing a similar mode of action as shown for the mammalian KDACs (Lombardi et al., 2011a). Based on sequence and structure *M. ramosa* AphA was classified into class II KDACs (Lombardi et al., 2011a). In the same year the first mammalian KDAC, HDAC1/KDAC1, was isolated that had the activity to deacetylate histones (Taunton et al., 1996). The class I enzyme HDLP (Histone deacetylase-like protein) from the Gram-negative bacterium *Aquifex aeolicus* and the class IIb enzyme HdaH (Histone deacetylase-like amidohydrolase) from the Gram-negative *Bordetella*/*Alcaligenes* strain FB188 were amongst the first KDACs that were identified as protein deacetylases and structurally characterized (Finnin et al., 1999; Hildmann et al., 2004; Nielsen et al., 2005; Meyners et al., 2014; Mahindra et al., 2019). As all mammalian classical deacetylases all bacterial KDACs adopt an α/β-fold consisting of a central parallel β-sheet that is surrounded by α-helices (Figure 6A; Finnin et al., 1999; Somoza et al., 2004; Nielsen et al., 2005; Kramer et al., 2016b). All bacterial KDACs are, as their mammalian counterparts, metalloenzymes coordinating a Zn^2+^-ion that is essential for catalysis (Finnin et al., 1999; Nielsen et al., 2005; Kramer et al., 2016b). *Bordetella*/*Alcaligenes* FB188 HdaH was shown to possess activity as protein deacetylase with preference for basic residues directly neighboring to the acetylated lysine side chains (Hildmann et al., 2004; Nielsen et al., 2005). Both enzymes, *Aquifex aeolicus* HDLP and HdaH from *Bordetella*/*Alcaligenes* FB188 were potently inhibited by the hydroxamate mammalian KDAC inhibitors TSA and SAHA (Finnin et al., 1999; Nielsen et al., 2005). Three putative acetylpolyamine amidohydrolases (APAHs) were reported to be encoded in the human pathogen *Pseudomonas aeruginosa*, namely PA0321, PA1409, and PA3774 (Winsor et al., 2011; Kramer et al., 2016b). While PA0321 and PA1409 are active in deacetylating polyamines, such acetyl-putrescine and acetyl-cadaverine, PA3774 does not use polyamines as substrates (Kramer et al., 2016a). PA1409 was additionally capable in deacetylating N(1)-acetyl-spermidine and N(1)-acetyl-spermine (Kramer et al., 2016a). The K_M_ values of PA0321 and PA1409 for polyamines are 0.2–0.5 mM, which is approximately one order of magnitude lower than the reported intracellular polyamine concentrations suggesting that the enzyme shows full activity under physiological conditions (Patel et al., 2006; Kramer et al., 2016a). Polyamines were reported to be important for cell growth and cell proliferation in archaea, eukaryotes and bacteria (Michael, 2016). In bacteria polyamines affect biofilm formation and they play an important role in translation in eukaryotes and archaea (Patel et al., 2006; Michael, 2016). Apart from their role in biofilm formation, the role of polyamines in bacteria is only marginally understood (Michael, 2016). A possible role might be that polyamines form a buffer for non-enzymatic acetylation under physiological conditions that favor non-enzymatic, chemical acetylation of proteins such as high concentrations of reactive acyl-CoA thioesters. Thereby, polyamines would constitute a detoxification system to prevent systemic protein dysfunction due to unspecific, non-enzymatic acetylation. Notably, all three *P. aeruginosa* enzymes showed activity toward a fluorescent Boc-AcK-AMC substrate, which also opens the possibility that these enzymes are capable to also deacetylate lysine side chains that are located at the far termini of the proteins (Kramer et al., 2016a). *P. aeruginosa* enzyme HdaH (PA3774) can be classified into class IIb KDACs and it shows enzymatic activity as lysine deacetylase and has no activity toward acetylated polyamines (Kramer et al., 2016a, b). Due to the structural and sequence similarity of PA3774 to HdaH from *Bordetella*/*Alcaligenes* FB188 a similar substrate spectrum is assumed (Hildmann et al., 2004; Kramer et al., 2016a, b). Substrates for *P. aeruginosa* PA3774 were postulated such as the histone-like DNA-binding protein HU. However, this was not experimentally validated and further studies are needed to unravel the physiological substrates of *P. aeruginosa* PA3774 and *Bordetella*/*Alcaligenes* FB188 HdaH (Hildmann et al., 2004; Kramer et al., 2016a, b). The structure of PA3774 shows a special feature, an elongated surface loop (L1 loop) that mediates oligomerization (Figure 6A; Kramer et al., 2016b). A tetramer is formed consisting of two “head-to-head” dimers (Kramer et al., 2016b). A similar oligomerization was also observed in the structure of HdaH from *Bordetella*/*Alcaligenes* FB188 (Nielsen et al., 2005). This oligomeric state might affect substrate binding and thereby it might constitute an important mechanisms for the determination of substrate specificity (Kramer et al., 2016b). In contrast, the structure of the *M. ramosa* AphA polyamine deacetylase shows formation of a dimer. However, the dimer is formed *via* a loop insert not present neither in *P. aeruginosa* PA3774 nor in *Bordetella*/*Alcaligenes* FB188 HdaH resulting in a different dimer arrangement. Interestingly, this loop insert is also present in *P. aeruginosa* PA0321 and PA1409 suggesting that this is one molecular determinant of substrate specificity toward acetylated polyamines (Figure 6A; Kramer et al., 2016a, b). For *Aeromonas hydrophila*, a Gram-negative opportunistic human pathogen propagating extracellularly and surviving intracellularly in host phagocytes, the class II enzyme AcuC was shown to be needed for biofilm formation and virulence suggesting that targeting AcuC activity might be a promising strategy to treat bacterial infections (Jiang et al., 2017). For the classical KDACs that were structurally characterized all essential catalytic residues are conserved (Figure 6B).

So far, none classical KDAC of a Gram-positive bacterial species has been structurally characterized. *B. subtilis* encodes a classical KDAC, AcuC that is encoded as part of the *acuABC* operon (acu: acetoin-utilization) (Grundy et al., 1993; Gardner and Escalante-Semerena, 2009). The gene products of the *acuABC*-operon were initially thought to be important for growth and sporulation in acetoin and butanediol. AcuA was shown to be an type III GNAT and AcuC a Zn^2+^-dependent classical deacetylase deacetylating and activating acetyl-CoA synthetase (AcsA). The gene *acsA* encoding AcsA is reversely transcribed upstream of the *acuABC* operon (Gardner et al., 2006). The role of AcuB is not known but the genomic organization of *acuB* within the *acuABC* operon suggests that it plays a role on regulation of AcsA acetylation or deacetylation. A similar genomic organization is also present in *S. aureus*. However, the *acuB* gene is missing in *S. aureus*. AcuC from Gram-positive *B. subtilis* and *S. aureus* can be classified into the class I of mammalian KDACs (Leipe and Landsman, 1997; Thiagalingam et al., 2003). AcsA was shown to be acetylated by AcuA inhibiting AcsA and AcuC is the deacetylase reversing this acetylation thereby activating AcsA (Gardner et al., 2006). Apart from AcsA, only TufA was identified as additional substrate for AcuC so far, which was also shown to be deacetylated by SrtN in *B. subtilis* (Suzuki et al., 2019). For AcuA no further substrates were found so far not excluding that further substrates apart from AcsA exist.

As a summary, although some bacterial classical KDACs were structurally and functionally characterized, their physiological roles are only marginally understood. To this end, future studies should focus on investigation of their physiological roles including identification of substrates.

## Acetylphosphate—The Major Driver for Non-Enzymatic Ac(Et)Ylation in Bacteria

**FIGURE 7:**
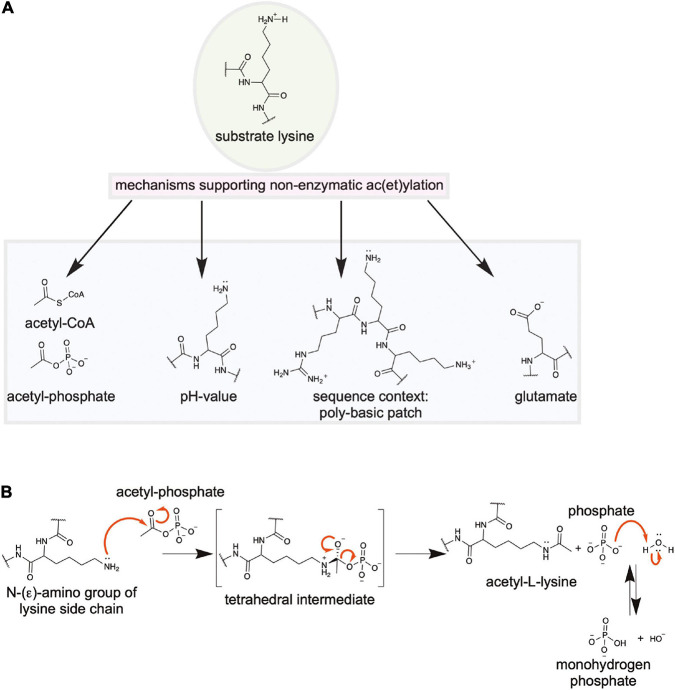
Non-enzymatic N-(ε)-lysine and/or N-(α)-N-terminal ac(et)ylation. **(A)** Several mechanisms contribute to the efficiency of non-enzymatic acetylation. The high energy ac(et)yl-CoA thioesters and the mixed anhydride acetyl-phosphate are very reactive molecules. Upon increase of the intracellular concentrations the level of systemic lysine and/or N-terminal ac(et)ylation is increased. Furthermore, an alkaline pH-value supports non-enzymatic ac(et)ylation as it affects the protonation state of the ε- and α-amino groups. Under more alkaline conditions the amino groups are in a more deprotonated state increasing their nucleophilicity prone for non-enzymatic ac(et)ylation. Moreover, the sequence context of the lysine side chain affects its reactivity. The presence of the substrate lysine side chain in poly-basic patches containing several basic residues such as arginine and lysine lowers the lysine side chains pK_a_ value resulting in a more reactive, deprotonated state under physiological pH. Finally, spatial vicinity and proper orientation of the substrate amino group to an acidic residue such as glutamate can favor its deprotonation increasing its nucleophilicity. **(B)** Reaction mechanism for non-enzymatic ac(et)ylation of substrate amino groups. In bacteria most of non-enzymatic acetylation is due to acetyl-phosphate. However, also ac(et)yl-CoA contributes to non-enzymatic ac(et)ylation in bacteria. Shown is the reaction of non-enzymatic lysine acetylation by acetyl-phosphate. A tetrahedral intermediate is formed following the nucleophilic attack of the deprotonated lysine side chain to the electrophilic carbonyl carbon of acetyl-phosphate. This intermediate decomposes to form ac(et)yl-lysine and CoA (figure redrawn and modified from Ali et al. (2018) and Blasl et al. (2021)].

Progress in quantitative mass-spectrometry enabled the identification of thousands of lysine acetylation/acylation sites in all domains of life. Ac(et)ylation of lysine side chains can be catalyzed enzymatically by lysine acetyltransferases such as the GNATs explained above using the respective ac(et)yl-CoA as donor molecule for the acylation of the ε-amino group of lysine side chains. However, next to enzymatic ac(et)ylation, lysine side chains and the α-amino groups in proteins can be ac(et)ylated non-enzymatically/chemically particularly under conditions that favor this reaction (Figure 7A). Notably, non-enzymatic acetylation does not mean that it is physiologically unimportant. In fact, non-enzymatic acetylation can even occur site-specifically and it can occur as part of a regulated cellular program such as metabolic fuel switching (Baeza et al., 2015; Olia et al., 2015). Systemic non-enzymatic acetylation can occur upon elevation of the concentration of cellular ac(et)yl-CoA. This depends on the cellular metabolic state under which various ac(et)yl-CoA molecules accumulate as explained above. Ac(et)yl-CoA molecules are highly reactive thioesters prone to react with nucleophilic groups such as ε-amino groups in lysine side chains or α-amino groups in proteins. As stated above, the concentrations of acetyl-CoA and malonyl-CoA in *E. coli* fluctuate between 200–600 μM and 4–90 μM, respectively (Chohnan et al., 1997, 1998; Hou et al., 2015). In exponentially growing, glucose-fed cultures of *E. coli*, acetyl-CoA concentrations of 610 μM were reported (Bennett et al., 2009). Acetyl-CoA concentrations depend on the metabolic state, it is highest during exponential growth phase and it declines during the stationary growth phase. While in mammals acetyl-CoA is the driver for non-enzymatic acetylation in eukaryotes, acetyl-phosphate was shown to be the major driver for non-enzymatic protein acetylation in bacteria (Verdin and Ott, 2013; Weinert et al., 2013a). In an exponentially growing, glucose-fed *E. coli* culture the concentration of acetyl-phosphate was reported to be 0.03–1.3 mM (McCleary and Stock, 1994; Pruss and Wolfe, 1994; Bennett et al., 2009). Acetyl-phosphate is produced in a reversible pathway of reactions catalyzed by acetate kinase (AK) and phosphotransacetylase (Pta) (Figure 5B). Pta catalyzes the formation of acetyl-phosphate from acetyl-CoA and orthophosphate, Pi (Verdin and Ott, 2013; Weinert et al., 2013a; Kuhn et al., 2014). Acetate kinase converts acetyl-phosphate to acetate and ATP (Figure 5B). The acetate can then be used as substrate for acetyl-CoA synthetase to produce acetyl-CoA. This pathway is used under conditions of carbon overflow for acetyl-CoA production in cells in which high cellular acetate concentrations (>5 mM) are accumulating (Krivoruchko et al., 2015; Schilling et al., 2015). The Michaelis-Menten constants, K_M_-values, of AK for acetate and of Pta for acetyl phosphate are reported to be relatively high, in the millimolar range (~7–10 mM) (Brown et al., 1977; Valgepea et al., 2010; Krivoruchko et al., 2015). It is the steady-state of both reactions that determine the cellular concentration of acetyl-phosphate. If acetate concentration is high under conditions such as carbon overflow, intracellular acetyl-phosphate can accumulate as AK produces acetyl-phosphate from acetate and ATP but Pta converts acetyl-phosphate slowly to acetyl-CoA due to its high, millimolar K_M_ for acetyl-phosphate. Notably, although it is suggested that acetyl-phosphate mainly drives non-enzymatic acetylation in bacteria it should be noted that of course accumulation of ac(et)yl-CoA will result in non-enzymatic acetylation in bacteria as described in eukaryotic cells as laws of chemistry hold independent of the cell type (Figure 7A).

Next to the accumulation of the ac(et)yl-donor molecules in the cell, the efficiency of non-enzymatic acetylation also depends on the protein primary sequence and the three dimensional structure. It was shown that presence of lysine side chains in poly-basic patches reduces the lysine side chain’s pK_a_ value increasing its nucleophilicity and reactivity making the ε-amino group of the lysine side chain prone for non-enzymatic ac(et)ylation by ac(et)yl-CoA and/or acetyl-phosphate (Figures 7A,B; Baeza et al., 2015; Olia et al., 2015; Schilling et al., 2015). This shows that a sequence might have evolved for non-enzymatic acetylation. Moreover, the spatial localization of the lysine side chain within the protein fold might influence its reactivity. Acidic side chains such as glutamate and aspartate in suitable distance and orientation to the lysine side chain within the protein fold may influence the electrostatics acting as general base like catalysts abstracting a proton and thereby increasing the lysine side chains reactivity (Figure 7A; Wagner and Hirschey, 2014; Baeza et al., 2015, 2016; Drazic et al., 2016).

Finally, for mitochondria it was shown that the high concentrations of acetyl-CoA in the matrix and the slightly more basic pH compared to the cytosol/nucleus (mitochondrial matrix: pH 7.8; cytosol/nucleus: pH 7.4) drives non-enzymatic acetylation. The pH value affects the protonation state of the lysine side chain and a reduction in the pH favors deprotonation and thereby increasing its nucleophilicity (Figure 7A). Most bacterial species are neutrophilic and live in habitats with a neutral pH value, i.e., the have an optimal pH range in between pH 6.0 and pH 8.0 grow (Padan et al., 1981, 2005; Macnab and Castle, 1987; Slonczewski et al., 2009; Panta and Doerrler, 2021). However several species are able to live in quite acidic or alkaline environments. Bacterial species are able to tolerate and to deal with rapidly changing pH values in their habitats (Padan et al., 1981, 2005; Macnab and Castle, 1987; Slonczewski et al., 2009; Panta and Doerrler, 2021). Their cytoplasmic membrane is impermeable for protons and ATP-dependent proton pumps and antiporters such as the Na^+^/H^+^- and the K^+^/H^+^-antiporters are used to ensure a constant neutral internal pH value. However, also short intervals of slightly basic internal pH values might be sufficient to support systemic non-enzymatic ace(et)ylation in neutrophilic bacteria. Moreover, alkaliphilic bacteria such as *Bacillus alcalophilus* maintain a constant internal basic pH value of pH 9.0–pH 9.5 suggesting that in these bacteria non-enzymatic acetylation might contribute strongly to overall lysine ac(et)ylation (Padan et al., 1981, 2005; Macnab and Castle, 1987; Slonczewski et al., 2009; Panta and Doerrler, 2021). Alternatively, these species might have evolved strategies to cope with these alkaline pH conditions regarding lysine ac(et)ylation. This was not investigated so far. The mechanism underlying non-enzymatic ac(et)ylation is similar to the enzymatically catalyzed reaction and proceeds *via* formation of an tetrahedral intermediate (Figure 7B).

As a summary, non-enzymatic ac(et)ylation is an important modification that modifies protein function. It can occur site-specifically in proteins that evolved in their sequence and structure for non-enzymatic ac(et)ylation to precisely regulate protein function. However, under conditions that favor non-enzymatic ac(et)ylation, this non-enzymatic ac(et)ylation also results in a systemic decline in protein functionalities due to the global accumulation of non-enzymatic acetylation in the proteome in a non-regulated manner. This is supported by recent quantitative mass-spectrometric analyses, which resulted in the identification and quantification of thousands of lysine ac(et)ylation sites in organisms of all domains of life. These data revealed the presence of a systemic low stoichiometry background lysine ac(et)ylation in proteomes of diverse organisms, which are most likely due to non-enzymatic ac(et)ylation (Choudhary et al., 2009; Weinert et al., 2011, 2013a,b, 2015, 2017; Lundby et al., 2012; Hansen et al., 2019). Organisms have invented strategies to cope with these processes. As an example, the mitochondrial sirtuin SIRT3 was shown to affect the acetylation state of almost 20% of all acetylated mitochondrial proteins suggesting that SIRT3 has evolved for high degree of substrate promiscuity to act as a detoxification enzyme in this metabolically highly active cell organelle (Hebert et al., 2013; Marcus and Andrabi, 2018). Similarly, most bacteria only encode for a single sirtuin deacetylase. For *E. coli*, CobB was shown to affect the ac(et)ylation state of thousands of proteins and deletion of *cobB* resulted in upregulation of 10% of all acetylation sites in *E. coli* under the conditions of the study (Weinert et al., 2013a). Moreover, CobB removes diverse acyl-groups from lysine side chains suggesting to play a similar role as detoxifying enzyme suppressing low stoichiometry ac(et)ylation that might occur due to non-enzymatic ac(et)ylation by accumulation of ac(et)yl-CoA and acetyl-phosphate (Peng et al., 2011; AbouElfetouh et al., 2015; Mei et al., 2016; Weinert et al., 2017; Dong et al., 2019; Wei et al., 2019). Furthermore, it was shown that CobB does neither show preference nor discriminate between enzymatic and non-enzymatic acetylation sites (AbouElfetouh et al., 2015).

## Are There Ac(Et)Yl-Lysine Reader Domains in Bacteria?

In eukaryotes next to the lysine ac(et)yltransferases (writers) and deac(et)ylases (erasers) also reader domains for ac(et)yl lysine were reported. These were the bromodomains (BRDs) and the YEATS (Yaf9, ENL, AF9, Taf14, Sas5) domains. Bromodomains were first identified in the protein Brahma in *Drosophila* (Tamkun et al., 1992). This protein is part of a multi-protein complex involved in chromatin remodeling. Today it is known that BRDs are specific acetyl-L-lysine binding domains composed of approximately 110 residues forming an unusual left-handed four-helix bundle (Dhalluin et al., 1999; Mujtaba et al., 2007). The binding site of the acetyl-L-Lysine is a hydrophobic cavity formed by loops connecting the α-helices. The specificity for binding to the acetylated protein is conferred by residues flanking the acetyl-L-lysine interacting with residues on the surface of the different bromodomains (Dhalluin et al., 1999; Mujtaba et al., 2007). So far a total of 61 BRDs were identified in 46 human proteins (Filippakopoulos et al., 2012; Filippakopoulos and Knapp, 2014). Many of the BRD-containing proteins were involved in chromatin remodeling, several KATs contain a BRD (Filippakopoulos et al., 2012).

So far, no BRD was identified in bacteria. Although the sequences do vary considerably, two conserved residues in the human BRDs are particularly important for binding of the substrate acetyl-L-lysine, an Asn forming hydrogen bonds with the acetyl-moiety and a Tyr establishes a water network in the acetyl-L-lysine binding cavity (Mujtaba et al., 2007). Future studies should reveal if there are BRDs encoded in the bacterial genome, either as individual proteins or as part for multi-domain proteins.

Apart from BRDs the YEATS domain was recently identified as a acetyl-L-lysine reader domain in human and in S. cerevisiae (Li et al., 2014). Structurally the YEATS domain does not resemble a BRD and the acetyl-L-lysine recognition is different. The YEATS domain adopts an immunoglobulin (IG)-fold composed of an eight-stranded antiparallel β-sheet. Similar to the BRD, binding to the acetyl-L-lysine is achieved *via* loops emanating from the IG-fold. For binding of the lysine acetylated substrate a serine-lined aromatic cage is used (Li et al., 2014). Analyses of the acetyl-lysine bearing proteins binding to YEATS domains shows overrepresentation of an arginine N-terminal to the acetyl-L-lysine. In analogy to the BRDs, the YEATS domain is found in several proteins that are involved in chromatin remodeling.

Future studies should show if bacteria employ ac(et)yl-L-lysine reader domains, either BRDs or YEATS-domains or unrelated domains, for recruitment of proteins by post-translational lysine ac(et)ylation. Moreover, signal transduction cascades are possible with presence of these recruitment domains similar to phosphorylation-dependent signal transduction cascades (Tang et al., 2007).

## Ce-Clan Related Enzymes With Dual Deubiquitinase And/Or Acetyltransferase Activity in Gram-Negative Pathogenic Bacteria

**FIGURE 8:**
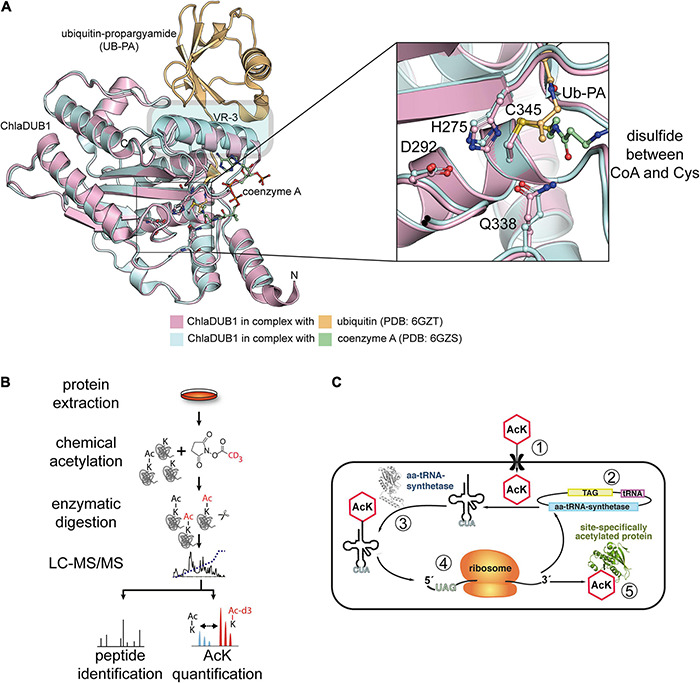
CE-clan related bacterial virulence factors with dual deubiquitinase (DUB)- and acetyltransferase (AcT)- activity and technological advances to study lysine ac(et)ylation. **(A)** Structure of the catalytic domain of *Chlamydia trachomatis* ChlaDUB1 (PDB: 6GZT; PDB: 6GZS). ChlaDUB1 was shown to act as DUB with specificity for K63-linked ubiquitin chains and as acetyltransferase for lysine, threonine and serine side chains. To discriminate this activity from lysine acetyltransferase activity, the abbreviation AcT is used. Notably, both activities, namely a deubiquitination, i.e., a hydrolysis reaction, and the transfer of an acetyl-group, i.e., a condensation reaction, is catalyzed by the same active site. The enzyme is a CE-clan related protease using a catalytic triad (order: His-Asp-Cys) for catalysis. Mutational studies showed that mutation of the catalytic cysteine to alanine (ChlaDUB1: C345A) abolished both activities. In contrast to other CE-clan related virulence factors that only are active as DUB or AcT, ChlaDUB1 contains an α-helix (VR-3: variable region-3) that mediates binding toward ubiquitin or acetyl-CoA/CoA using different surfaces. The structure with ubiquitin was obtained by using a ubiquitin activity-based probe ubiquitin-propargylamide (Ub-PA), resulting in covalent linkage of the Ub-PA to the active site Cys. Coenzyme A (CoA) was also covalently bound by formation of a disulfide bond between the CoA cysteamine and the active site Cys [the figure was generated with PyMOL v.2.3.4 (Schrödinger LLC, 2000)]. **(B)** Improved mass-spectrometry workflows to obtain information of the lysine ac(ety)lation stoichiometry on a systemic scale. Initially, mass-spectrometry was used to identify lysine acetylation sites in proteins without obtaining information on the dynamics or stoichiometry. Workflows that were developed, such as SILAC, allow to systemically compare alterations in lysine ac(et)ylation in diverse cellular states. Finally, chemical labeling of all lysine side chains (and N-terminal amino groups) in proteins (before enzymatic digestion) using isotopically labeled molecules such as N-acetoxy-succinimide or acetic-anhydride allows to perform absolute quantification of ac(et)ylation on a global scale. This workflow can be combined with SILAC and internal standards to generate powerful workflows to assess the kinetics and stoichiometry of ac(et)ylation. **(C)** Genetically encoding N-(ε)-ac(et)yl-lysine in proteins using the genetic code expansion concept (GCEC). By applying an orthogonal and synthetically evolved ac(et)yl-lysyl-tRNA (AcKRS)/tRNA_CUA_ pair based on the pyrrolysyl-tRNA_CUA_ (PylRS)/PylT pair from *Methanosarcina barkeri* or *M. mazei* acetyl-L-lysine and other lysine acylations, such as, propionyl-, butyryl-, crotonyl-lysine can be site-specifically incorporated into any protein as response to an amber stop codon. The system is orthogonal in all used model organisms. (1) Cells are fed with acetyl-L-lysine, deacetylases can be inhibited by addition of selected deacetylase inhibitors, (2) cells express the evolved AcKRS/tRNA_CUA_ pair, (3) AcKRS charges the cognate amber suppressor tRNA_CUA_ with the ac(et)yl-lysine, (4) Ac(et)yl-lysine is co-translationally incorporated into proteins as response to an amber stop codon using the host cells translation machinery. Using this technology enables to obtain natively-folded and quantitatively lysine ac(et)ylated proteins for structural and functional studies [figure modified from Lammers et al. (2019) and Blasl et al. (2021)].

Based on the classification of the MEROPS database the CE-clan contains several protein families of cysteine endopeptidases that are only distantly related. This CE-clan was found to contain several enzymes from human Gram-negative bacterial pathogens that act as efficient deubiquitinase (DUB), some act as acetyltransferases (AcT) and others have a dual activity acting as DUB and AcT. These enzymes are structurally not related to other lysine acetyltransferases, they exert a different catalytic mechanisms and they have the capability to acetylate serine and threonine residues next to lysine side chains (Zhou et al., 2005; Mittal et al., 2010; Ma and Ma, 2016; Pruneda et al., 2016, 2018; Hermanns and Hofmann, 2019; Hermanns et al., 2020). Phylogenetic analyses revealed that most CE-clan DUBs contained a conserved aromatic residue in the active site that distinguishes them from other cysteine protease families (Hermanns et al., 2020). This aromatic residue allows cleavage at the C-terminal di-Gly motif in ubiquitin (Hermanns et al., 2020). All of these proteins are used by human pathogenic bacteria and they are injected into host cells to allow an efficient infection process. Some were shown to interfere with mitogen-activated protein kinase (MAPK)-signaling and with NFκB-signaling interfering with the host cells inflammatory response. The CE-clan enzymes use a catalytic mechanisms that involves a catalytic triad in the order His-Asp/Asn-Cys in the primary sequence, with the Cys acting as nucleophile (Hermanns and Hofmann, 2019; Figure 8A). A Gln following the His of the catalytic triad is often involved in formation of the oxyanion hole during catalysis (Hermanns and Hofmann, 2019). It is remarkable that these enzymes can catalyze these different reactions, namely a hydrolysis reaction, i.e., deubiquitinase, and a condensation reaction, i.e., the acetyl group transfer, using the same active site. Structurally these CE-clan related DUBs/AcTs are composed of two subdomains, a β-barrel subdomain containing the His-Asp/Asn of the catalytic triade and an α-helical bundle subdomain containing the catalytic Cys. The substrate binding occurs between both subdomains (Figure 8A).

The first enzyme to be identified of this class was the *Yersinia pestis* virulence factor YopJ. Although initially assumed to act as SUMO protease or DUB, today it is assumed that YopJ sole enzymatic activity of physiological significance is its acetyltransferase activity (Orth et al., 2000; Zhou et al., 2005). YopJ is injected into host cells acting as acetyltransferase for kinase MEK2 and for both subunits of the NFκB kinase IKK (Mittal et al., 2006). Notably, YopJ acts as Ser/Thr-acetyltransferase in both cases and acetylation of Ser residues in the activation loops of MEK2 and of a Thr residue in IKK causing inactivation of both kinases. The Ser/Thr residues are in the kinases’ activation loops and acetylation blocks their phosphorylation which is needed for kinase activation. Thereby, YopJ reduces the inflammatory response and induces apoptosis in immune host cells (Mittal et al., 2006). Later studies showed that YopJ activity is activated by inositol hexakisphosphate (IP6) suggesting a mode by which activation of YopJ is restricted to host cells as bacteria do not contain IP6 (Mittal et al., 2010). Three YopJ-related acetyltransferases were structurally characterized, the *S. typhimurium* protein AvrA, the protein HopZ1 from the plant pathogen *Pseudomonas syringae* and the PopP2 from the plant pathogen *Ralstonia solaneacearum* (Zhang et al., 2016; Zhang Z. M. et al., 2017; Labriola et al., 2018). Similar as observed for YopJ, it was shown that the activity of AvrA was dependent on the presence of IP6 and AvrA inactivated MAP-kinase signaling through acetylation and inactivation of MKK4/7 (Labriola et al., 2018). Besides from these CE-clan related bacterial effectors also the *Legionella* effectors Lpg2907 (LegCE) and Lpg1949 were shown to be dedicated acetyltransferases (Pruneda et al., 2018; Hermanns et al., 2020). For Lpg1949 we showed that it is active in acetylating MEK6 (Hermanns et al., 2020). If these AcTs have further substrates in the host cells has not been systematically analyzed so far.

Various CE-clan related bacterial proteases with deubiquitinase and/or acetyltransferase activity were biochemically and structurally characterized so far. The structures of catalytic domains of the CE-clan DUBs SseL, ChlaDUB1, and RickCE were solved by X-ray crystallography (Figure 8A; Pruneda et al., 2016). These have various preferences for cleaving ubiquitin, or the ubiquitin-like proteins (ULPs) SUMO, NEDD8, and ISG15 (Pruneda et al., 2016, 2018). SseL from *Salmonella typhimurium*, ElaD from *E. coli* and ShiCE from *Shigella flexneri* are Ub-specific DUBs, while ChlaDUB1 from *Chlamydia trachomatis* and RickCE from *Rickettsia bellii* were shown to cleave Ub- and also NEDD8-chains (Catic et al., 2007; Rytkonen and Holden, 2007; Rytkonen et al., 2007; Pruneda et al., 2016; Fischer et al., 2017). Most bacterial DUBs show preferences for K63-linked Ub-chains, while some do show lower activity also toward K48- and K11-linked Ub-chains. The *Legionella* effector SdeA cleaves K63-, K48-, and K11-linked Ub-chains while ChlaDUB1, ElaD, and ShiCE are specific for only K63-linked chains. It is believed that the activity of most DUBs from Gram-negative pathogenic bacteria to cleave K63-linked Ub-chains in is to remove ubiquitin chains of bacteria-containing vacuoles to avoid their degradation through the host cell and thereby supporting their intracellular maintenance (Fischer et al., 2017; Kubori et al., 2018).

The *Chlamydia trachomatis* effector ChlaDUB1 was recently characterized as K63-Ub-chain specific DUB. Moreover, it was shown that ChlaDUB1 is an active acetyltransferase (Pruneda et al., 2018). The crystal structures of ChlaDUB1 in complexes with coenzyme A and ubiquitin revealed how these two enzymatic activities are exerted by the same active site (Figure 8A; Pruneda et al., 2018). The structures revealed the presence of an α-helix (VR-3: variable region-3) not present in other CE-related DUBs from bacteria. Residues of two faces of this α-helix mediate binding toward ubiquitin and coenzyme A. the active site including the catalytic triad H275/D292/C345 is in the right location to catalyze acetyl-transfer from acetyl-CoA and cleavage of K63-linked Ub-chains (Figure 8A; Pruneda et al., 2018). ChlaDUB1 was shown that the induced Golgi fragmentation in the host cells was due to its DUB activity opening the question for the role of the AcT activity during infection (Pruneda et al., 2018). Some studies suggest a role of ChlaDUB1 on inhibition of NFκB signaling but if this is mediated *via* its AcT activity needs further investigation (Le Negrate et al., 2008). While these analyses revealed the molecular basis for the dual DUB/AcT activity for ChlaDUB1 from *Chlamydia trachomatis*, the molecular mechanisms underlying the specificity of bacterial CE-clan related bacterial DUBs toward different Ub-chains is still not completely resolved. Furthermore, it is only incompletely understood which cellular processes are tackled by the different DUBs and AcTs to support an efficient infection. Along this line, it will be interesting to investigate why some bacterial species such as *Rickettsia* or *Legionella* encode several CE-clan related DUBs with slightly different Ub-chain type preferences. A possible mechanism of bacterial CE-clan DUBs/AcTs is furthermore that the AcT activity is used to block subsequent ubiquitination by the host cell. This model needs to be analyzed in the future.

## Advances in Mass-Spectrometry and Synthetic Biology Drive Our Knowledge on Lysine Ac(Et)Ylation in Bacteria

Critical for the progress in a research field is the continuous development and progress of novel technologies. In the field of lysine ac(et)ylation it was the huge progress in the development in quantitative mass-spectrometry and in synthetic biology that helps to get a better understanding of this important post-translational modification in all domains of life. The following sections describes developments in two major fields contributing to this progress, namely quantitative mass-spectrometry and synthetic biology.

### Mass-Spectrometry: Improved Workflows Allow to Study Stoichiometry of Lysine Ac(et)ylation

Beginning of 1960s lysine acetylation was discovered to occur on histones affecting RNA synthesis (Phillips, 1963; Allfrey and Mirsky, 1964; Allfrey et al., 1964). Afterward, it were only individual proteins identified as being lysine acetylated such as α-tubulin (L’Hernault and Rosenbaum, 1985). In the year 2000 the deacetylase SIR2 from *S. cerevisiae* was shown to be an NAD^+^-dependent lysine deacetylase with implications on the aging process (Kaeberlein et al., 1999; Imai et al., 2000). In the same period the first bacterial proteins were identified as being lysine acetylated, namely acetyl-CoA synthetase from *S. enterica* and the chemotaxis protein CheY from *E. coli* (Ramakrishnan et al., 1998; Barak and Eisenbach, 2001; Starai et al., 2002). The technological progress in mass-spectrometry enabled the identification of thousands of lysine ac(et)ylation sites in diverse bacterial species. This was due to the development of tools such improved mass-spectrometers concerning its dynamic range and sensitivity and the availability of specific antibodies recognizing anti-acetyl-L-lysine and other lysine acylations suited for immunoenrichment of ac(et)ylated peptides. However, as these antibodies are not unbiased regarding the sequences surrounding the acetyl-L-lysine often mixtures of available antibodies were used for immunoenrichment on the peptide level prior to LC-MS/MS analyses. This immunoenrichment step strongly improved the depth of the analyses represented by the number of acetylated peptides identified. Mass-spectrometric screens enabled the identification of thousands of lysine acetylation sites in bacteria. The first systemic mass-spectrometric screen performed on an *E. coli* proteome resulted in the identification of 125/138 acetylation sites in 85/91 proteins, respectively, predominantly involved in enzymes of the main metabolic pathways (Yu et al., 2008; Zhang et al., 2009). Today, more than 50 acetylomes were reported from diverse Gram-positive and Gram-negative bacterial strains and the number is continuously growing (Nakayasu et al., 2014, 2017; Christensen et al., 2019a, b). For most of these screens an immunoenrichment on the peptide level following proteolytic digest was performed with an anti-acetyl-lysine antibody. Notably, the fact that the number of acetylated proteins was strongly reduced in workflows without this immunoenrichment shows that the overall stoichiometry of the acetylation is low. Along this line, many of the sites identified in these workflows might not be of physiological significance as these are pure background noise present due to non-enzymatic acetylation by acetyl-phosphate and/or acetyl-CoA. It is one of the biggest challenges in the research field to unravel which of the many ace(et)ylation substrate sites are of real physiological importance. To this end, a huge study performed with 48 phylogenetically unrelated bacterial species analyzed the acetylomes without immunoenrichment and identified more than 9,000 acetylation sites (Nakayasu et al., 2017; Christensen et al., 2019a, b). It is likely that these sites detected without enrichment are important regulatory sites and in fact this study showed that many sites are conserved in enzymes of the main metabolic pathways (Nakayasu et al., 2017).

The workflows were improved to be able to determine the dynamics of lysine ac(et)ylation by chemical labeling on the peptide level as done in iTRAC or TMT labeling or by metabolic labeling such as done in SILAC (stable-isotope labeling by amino acids in cell culture), comparing different interventions (Ong and Mann, 2007; Choudhary et al., 2009; Sol et al., 2012; Hebert et al., 2013; Baeza et al., 2014; Carabetta and Cristea, 2017). There are several great reviews summarizing the mass-spectrometry screens performed on diverse bacterial species to assess dynamics of acetylomes comparing different interventions or physiological states (Carabetta and Cristea, 2017; Christensen et al., 2019a, b). A major technological breakthrough in acetylation research was the development of mass-spectrometric workflows that allow the determination of lysine ac(et)ylation stoichiometry on a systemic scale (Figure 8B; Baeza et al., 2014, 2015, 2016, 2020; Nakayasu et al., 2014; Weinert et al., 2015, 2017, 2018; Hansen et al., 2019; Lindahl et al., 2019). These are based on labeling of all accessible lysine side chains by isotopically labeled N-acetoxy-succinimide or acetic anhydride (Supplementary Figure 2) at the protein level prior to tryptic digest. Some workflows even combined SILAC and chemical acetylation or included internal standards to assess the dynamics of acetylation and obtain information on the stoichiometry of acetylation (Figure 8A; Weinert et al., 2015, 2017, 2018; Hansen et al., 2019; Lindahl et al., 2019). This allows to perform absolute quantification of the endogenous acetylation level and comparison of different states. Even time-resolved studies were performed in mammals to unravel the role of lysine acetyltransferases and the major cellular substrates and targets (Weinert et al., 2018; Baeza et al., 2020; Haws et al., 2020). The knowledge on the achieved acetylation stoichiometry is essential to assess the physiological importance of a specific lysine ac(et)ylation event. As an example, if a post-translational acetylation has a loss-of-function effect on a protein function, the modification must be present in rather high stoichiometry to be of physiological significance. Alternatively, if several enzymes of a metabolic pathway are affected by lysine acetylation to some extend it can accumulate to a significant outcome at the end of the pathway. The same is true for multi-subunit proteins, in which the acetylation in one subunit might affect the activity of the whole protein in a cooperative manner. If a modification creates a new functionality, in the sense of a gain-of-function, the stoichiometry does not need to reach high stoichiometries to be of significance. As an example, in a amplifying signal transduction cascade, such as present in chemotactic signaling in *E. coli*, affecting the activity of a minor proportion of one enzyme, such as activation of CheY by acetylation, might be sufficient to evoke a substantial response (Barak et al., 2006; Yan et al., 2008). The so far mostly for eukaryotic systems applied improved mass-spectrometric workflows that allow the systemic determination of ac(et)ylation stoichiometries combined with workflows that assess the ac(et)ylation dynamics comparing different interventions or physiological states, should be also applied to bacterial systems to narrow down which of the thousands of identified lysine ac(et)ylation sites are of physiological significance.

### Synthetic Biology: Genetically Encoding Acetyl-L-Lysine in Proteins Allows to Unravel the Real Consequences of Lysine Acetylation on Protein Function

Our understanding on the mechanism how lysine acetylation mechanistically regulates protein function was mainly driven by the development of a system that allows to genetically encode acetyl-L-lysine in proteins—the genetic code expansion concept (GCEC) (Figure 8C). This is based on a synthetically evolved acetyl-lysyl-tRNA-synthetase (AcKRS)/tRNA_CUA_ (PylT) pair from *Methanosarcina barkeri* or *Methanosarcina mazei* originating on the pyrrolysyl-tRNA_CUA_-synthetase (PylRS)/tRNA_CUA_ (PylT) pair. Methanogenic archaea of the genus *Methanosarcina* incorporate pyrrolysine as 22nd proteinogenic amino acid into enzymes involved in methane metabolism (Atkins and Gesteland, 2002; Hao et al., 2002). To this end, they encode PylRS, which charges the cognate tRNA_CUA_, PylT, with pyrrolysine, which is incorporated into the enzymes as response to an amber stop codon (5′-UAG-3′). PylRS was evolved synthetically to charge the cognate PylT with acetyl-L-lysine instead of pyrrolysine (Neumann et al., 2008, 2009; Nguyen et al., 2009). As the PylRS/PylT pair from *Methanosarcina* is orthogonal in all model organisms this allows to incorporate acetyl-L-lysine as response to an amber stop codon into the protein of interest. Importantly, the system allows to obtain protein that is site-specifically and quantitatively lysine-acetylated and natively folded. We used the system in *E. coli* to produce proteins in yield and in a quality sufficient to perform biochemical and biophysical studies including structural analyses by X-ray crystallography (Figure 8C; Lammers et al., 2010; de Boor et al., 2015; Knyphausen et al., 2016a; Kuhlmann et al., 2016a, b; Lammers, 2018). We solved the first crystal structures of proteins carrying one or even two post-translationally relevant acetyl-lysines (Lammers et al., 2010; Kuhlmann et al., 2016a, b). Based on these studies we were able to derive mechanisms by which lysine acetylation regulate protein function (Lammers et al., 2010; de Boor et al., 2015; Knyphausen et al., 2016a; Kuhlmann et al., 2016a, b). Acetylation of the ε-amino group of lysine side chains neutralizes the positive charge at the lysine side chain, it alters the size of the amino acid, it affects the hydrophobicity of the side chain, it alters the surface complementarity and it also crosstalks with other post-translational modifications. We used the GCEC to produce site-specifically lysine-acetylated and natively-folded proteins as substrates for KDAC/sirtuin catalyzed deacetylation (de Boor et al., 2015; Knyphausen et al., 2016a; Lammers, 2018). This allows to analyze the molecular determinants of substrate specificity for the deacetylases. These studies revealed that the three-dimensional structure is next to the primary structure a major determinant for the substrate specificity (Knyphausen et al., 2016a; Lammers, 2018). Variants of AcKRS/PylT were developed that allow the incorporation of the acetyl-L-lysine analogs thioacetyl-lysine and trifluoroacetyllysine. As stated above these analogs are almost not deacylated by sirtuins representing valuable tools to block deacetylation increasing the lifetime of a lysine acetylation at a specific site for investigations *in vivo* or *in vitro* (Hancock et al., 2010; Venkat et al., 2017b). Notably, these analogs are better substrates compared to acetyl-L-lysine for some classical KDACs due to their increased electrophilicity at the carbonyl carbon of the thio-/trifluoroacetyl group (Lahm et al., 2007; Smith et al., 2008). Preparation of thioacetylated or trifluoroacetylated proteins in huge quantities and in high purity would allow to structurally characterize sirtuin-substrate complexes which was not successful so far. The available structural data is restricted on sirtuin complexes with acylated peptides (Spinck et al., 2020).

Recently, a novel prokaryotic class of deacetylases was suggested to be encoded in Gram-negative bacteria (Tu et al., 2015). The representative protein YcgC in *E. coli* was analyzed and shows no similarity in sequence and structure to any known deacetylases. It was suggested that YcgC exerts a catalytic mechanism involving a catalytic serine residue (Tu et al., 2015). Amongst the suggested substrates was the transcriptional regulator RutR involved in transcriptional regulation of pyrimidine metabolism. RutR was suggested to be quantitatively lysine acetylated on two lysine residues, K52 and K62, in the region of the helix-turn-helix motif important for DNA binding. Our laboratory applied the GCEC to produce K52- and K62-acetylated RutR proteins (RutR AcK52 and RutR AcK62) and used these proteins to assess YcgC and CobB catalyzed deacetylation. We showed that YcgC does not show any deacetylase activity toward acetylated RutR, while CobB is capable to deacetylate RutR (Kremer et al., 2018).

Moreover, the GCEC can be applied to assess the real consequences of lysine acetylation rather than performing studies with the Lys to Gln or Lys to Arg mutants, which are often applied for studies *in vivo* and *in vitro* to either mimic a lysine acetylation or to conserve the non-acetylated, positively charged state, respectively (Figure 1B; de Boor et al., 2015; Knyphausen et al., 2016b). These mutants were shown to be sometimes poor tools to study lysine acetylation. As an example, the structure of Gln does not resemble acetyl-L-lysine in size (Figure 1B). If the sole mechanism how lysine acetylation affects a protein function is charge neutralization, this might be reflected by mutation of Lys to Gln. However, if the lysine acetylation also acts *via* a steric contribution, a Gln is a poor mimic for acetylation (de Boor et al., 2015; Knyphausen et al., 2016b). Along that line, the mutation of Lys to Arg, which was initially used to preserve and assess the non-acetylated state, might reflect the steric contribution of lysine acetylation as Arg resembles acetyl-L-lysine more than Gln (Figure 1B). This has to be considered when analyzing the results obtained by these mutational studies. In a study analyzing the influence of acetylation of K180 (AcK180) on the DNA-binding capacity of the *E. coli* transcription factor RcsB, the mutants RcsB K180Q and RcsB K180R were analyzed (Thao et al., 2010). In this example, both RcsB K180Q and RcsB K180R almost completely abolished DNA-binding. This shows that RcsB AcK180 mechanistically impairs DNA-binding *via* charge neutralization (represented by K180Q) and *via* a steric contribution (represented by K180R) (Thao et al., 2010).

In another study the genetic code expansion was applied for incorporation of acetyl-L-lysine into *E. coli* isocitrate dehydrogenase (ICDH) at diverse sites. Notably, while some lysine acetylation events impaired the catalytic activity of ICDH some increased the catalytic activity in comparison to the non-acetylated enzyme, i.e., these show a gain-of-function. This activity stimulating effect was attributed to an potential affinity increasing effect toward the cofactor NADP^+^ and an influence on active site conformation (Venkat et al., 2018).

Other reports applying the GCEC for were performed in *Corynebacterium glutamicum* phosphoenylpyruvate carboxylase showing that acetylation at K653 impairs the enzymatic activity and that this acetylation is reversible (Nagano-Shoji et al., 2017). Moreover, genetically encoding acetyl-L-lysine results in the decrease in *E. coli* alanyl-tRNA-synthetase and tyrosyl-tRNA-synthetase activity (Venkat et al., 2017a; Umehara et al., 2018).

Further systems were developed that allow to genetically encode unnatural amino acids in proteins. The tyrosyl-tRNA-synthetase/tRNA_CUA_ from *Methanocaldococcus jannaschii* was evolved to allow incorporation of the photoactivatable crosslinkers *p*-benzoyl-L-phenylalanine and *p*-azido-L-phenylalanine proteins (Kauer et al., 1986; Chin et al., 2002a, b). This enables to photo-crosslink interaction partners and to stabilize otherwise transient interactions. This has been applied to human KDAC8 to identify novel substrates (Lopez et al., 2017). Next to photocrosslinkers, other genetic code expansion systems allow the site-specific incorporation of fluorescent amino acids, some of which are even photocageable, such as *o*-nitrobenzoyl-lysine, to follow protein dynamics in cells enabling light control of cellular processes using live cell fluorescence microscopy (Gautier et al., 2010, 2011; Lopez et al., 2017; Illes et al., 2021; Wolffgramm et al., 2021). Moreover, systems to genetically encode ubiquitin, phosphoserine and phosphothreonine were also developed that enables to study the impact of ubiquitination and phosphorylation on protein function (Chin, 2011, 2017; Park et al., 2011; Rogerson et al., 2015; Zhang M. S. et al., 2017; de la Torre and Chin, 2021). As stated above using mimetic mutations such as Ser/Thr to Glu/Asp, to study Ser/Thr phosphorylation can be misleading as described for mimetic mutations in studying lysine acetylation. The efficiency of multiple site incorporation of different amino acids in proteins was enhanced using different strategies. *E. coli* strains were developed that carry a genomic deletion of the gene encoding for release factor 1 (RF1), which recognizes the amber stop codon (5′-UAG-3′) thereby competing with the amber suppressor tRNA_CUA_ (Johnson et al., 2011). Moreover, *E. coli* strains were developed that carry whole genome deletion of amber stop codons avoiding incorporation of the unnatural amino acid at unwanted sites in endogenous proteins (Mukai et al., 2010). Often decoding the amber stop codon is no problem, as the amber stop codon is the least often used stop codon in *E. coli.* Moreover, the other stop codons opal (5′-UGA-3′) and ochre (5′-UAA-3′), rare sense codons or even quadruplet codons can be (re)assigned for unnatural amino acid incorporation (Neumann et al., 2010; Johnson et al., 2011; Heinemann et al., 2012; Wang et al., 2012; Mukai et al., 2015; Ho et al., 2016; Schmitt et al., 2018). Finally, even orthogonal ribosomes were created that are directed to an orthogonal messages allowing multisite incorporation of unnatural amino acids decoding several amber stop codons (Wang et al., 2007). The development of multiple orthogonal systems that allow the incorporation of different unnatural amino acids into proteins at different sites allows to study the crosstalk of different post-translational modifications. As an example, this could be applied to the *E. coli* chemotaxis regulator CheY, which is known to be regulated by phosphorylation and acetylation (Barak and Eisenbach, 2004). Our laboratory showed for the regulator RhoGDIα of the GTP-binding protein RhoA that lysine acetylation interferes with RhoGDIα SUMOylation (Kuhlmann et al., 2016a).

Future studies should involve the genetic code expansion concept to unravel how function of bacterial proteins and as a consequence also physiological process in bacteria are regulated by lysine acetylation. Most genetic code expansion systems, such as the *Methanosarcina* AcKRS/PylT system, were shown to be orthogonal in all model organisms tested and can be applied for studies *in vitro* and *in vivo* (Hancock et al., 2010; Greiss and Chin, 2011; Bianco et al., 2012; Elsasser et al., 2016; Han S. et al., 2017; Brown et al., 2018; Reille-Seroussi et al., 2019; Nikic-Spiegel, 2020). Application of genetic code expansion tools such as genetically encoded photoactivatable crosslinkers or fluorescent amino acids can be applied to bacteria to deepen our understanding on the physiological impact of lysine ac(et)ylation to control cellular processes.

## Physiological Roles of Post-Translational Lysine Ac(Et)Ylation in Bacteria

Lysine ac(et)ylation was shown to be an important post-translational modification in all domains of life. It rivals in terms of numbers of proteins that are ac(et)ylated and in number of sites post-translational phosphorylation (Kouzarides, 2000). As shown for phosphorylation, where kinases and phosphatases act to achieve reversibility in the PTM, for ac(et)ylation, lysine ac(et)yltransferases and deac(et)ylases were known that confer reversibility. As explained in the upper sections the unique feature of lysine ac(et)ylation is its close interconnection to the cellular metabolic state. In fact, regulation by lysine ac(et)ylation can be regarded as a system to adapt cellular physiology to the cellular metabolic state. This is reflected on several layers: (a) regulation of KAT activity by the intracellular a(et)yl-CoA/CoA ratio, (b) allosteric regulation of KAT activity by amino acids, ac(et)yl-CoA, cAMP, NADP^+^, (c) regulation of sirtuin activity by the cellular NAD^+^ concentration, and (d) regulation of sirtuin activity by allosteric modulators such as c-di-GMP. In the following section some important examples will be explained to highlight the importance of post-translational lysine ac(et)ylation for bacterial physiology. Recent proteomics data revealed that metabolism, transcription and translation are major cellular processes affected by lysine ac(et)ylation in bacteria (Weinert et al., 2017; Lindahl et al., 2019). Several excellent reviews on the influence of lysine acetylation on bacterial physiological processes are published (Carabetta and Cristea, 2017; Christensen et al., 2019b; VanDrisse and Escalante-Semerena, 2019). To this end, in the following section only selected examples are explained for which the role of lysine ac(et)ylation is understood at the mechanistic level.

## Metabolism

The paradigm on the regulation of metabolism by lysine acetylation is the enzyme acetyl-CoA synthetase (Acs), which was initially studied on *S. enterica* Acs. It was shown in Gram-negative and Gram-positive bacteria that AMP-forming Acs is regulated by acetylation of a conserved lysine residue in its C-terminus resulting in catalytic inactivation (Starai et al., 2002, 2005; Barak et al., 2004; Gardner and Escalante-Semerena, 2009; Tucker and Escalante-Semerena, 2014; Han X. et al., 2017; VanDrisse and Escalante-Semerena, 2018). Acetyl-CoA synthetase catalyzes the formation of acetyl-CoA and ADP from acetate, ATP and CoA *via* two half reactions (Figure 5B). The first half reaction results in activation of acetate by adenylation to form the mixed anhydride acetate-AMP under consumption of ATP and release of pyrophosphate. The pyrophosphate can drive the reaction making it irreversible by pyrophosphatase converting pyrophosphate to two molecules of orthophosphate. In the second half reaction the acetate is transferred from acetyl-AMP to CoA to form acetyl-CoA, an activated thioester. Thereby, the Acs-catalyzed formation of acetyl-CoA fulfills exclusively anabolic functions (Kumari et al., 2000). The K_M_ value of *E. coli* Acs for acetate is approximately 0.2 mM suggesting that Acs is important for production of acetyl-CoA to drive metabolic pathways such as TCA cycle and the glyoxylate shunt for energy production and biosynthesis under conditions of low intracellular acetate concentrations (Kumari et al., 2000; Valgepea et al., 2010). As stated above, K_M_ values for acetate are substantially higher, in the millimolar range, for *S. enterica* and *M. tuberculosis* Acs (Brown et al., 1977; Reger et al., 2007; Li et al., 2011). In the exponential growth phase under conditions of carbon overflow, the AK/Pta pathway is activated to metabolize acetyl-CoA as it exceeds the amphibolic capacity of the main metabolic pathways, i.e., the TCA cycle, resulting in formation of acetyl-phosphate and acetate, which is excreted into the medium (Kumari et al., 2000; Schilling et al., 2015). When *E. coli* cells enter stationary growth phase they start to reabsorb acetate to reach concentrations sufficient to activate the Acs pathway but not the acetate-kinase/phosphotransacetylase pathway, as *E. coli* Acs has a K_M_ of 0.2 mM and AK/Pta have K_M_-values value of 7–10 mM for acetate (Kumari et al., 2000; Valgepea et al., 2010). The regulation of Acs by lysine acetylation is evolutionary conserved and was reported to occur in bacteria, plants, mammals and archaea (Starai et al., 2002; Hallows et al., 2006; Cao et al., 2019). The crystal structure of K609-acetylated Acs from *S. enterica* showed no significant conformational differences to the non-acetylated enzyme suggesting that the acetylation at K609 plays a role on the catalytic mechanism (Gulick et al., 2003). Mechanistically the acetylation in the C-terminus (*S. enterica*: K609) by *Se*Pat inactivates Acs activity by affecting the first half reaction, i.e., formation of acetyl-AMP from acetate and ATP as suggested from the crystal structure (Gulick et al., 2003; Starai and Escalante-Semerena, 2004b). K609 In *S. enterica* Acs is suggested to be involved in proper productive orientation and alignment for catalysis which is abolished in the acetylated state (Gulick et al., 2003; Cao et al., 2019). Several reports suggest that acetyl-CoA synthetase itself has the capacity to act as lysine acetyltransferase. As an example, the chemotaxis regulator CheY was shown to be acetylated by acetyl-CoA synthetase (Barak et al., 2004). Another report even claims that CheY has autoacetylation activity without acetylating other proteins and it is deacetylated by Acs (Barak et al., 2006). In that context a possible explanation might be that CheY is prone for non-enzymatic acetylation due to acetyl-CoA accumulating locally by Acs activity. Acs was shown to be regulated by cAMP at various layers on the transcriptional and post-translational level. Firstly, cAMP mediates CAP/CRP mediated expression of *acs* under conditions of carbon limitation (Beatty et al., 2003; Browning et al., 2004). Secondly, cAMP acts as competitive inhibitor of Acs binding to the AMP/ATP-binding pocket (Starai and Escalante-Semerena, 2004a; Han X. et al., 2017). Thirdly, cAMP-binding to Acs induces a conformational change that favors it for *Se*Pat-catalyzed acetylation and disfavoring it for CobB mediated deacetylation (Starai et al., 2002; Starai and Escalante-Semerena, 2004b). This results in an overall activation of Acs activity. Indirectly, cAMP also induces transcription of the *pat*-gene also resulting in an increase in Acs activity.

Mass-spectrometry based screens revealed that several metabolic pathways, such as TCA cycle, pyruvate metabolism and glycolysis, and many enzymes involved in these pathways are enriched in lysine acetylation (Baeza et al., 2014). For most of the acetylations in metabolic enzymes, an activity decreasing effect was observed and only few result in an activation or in a modulation of enzyme activity. As examples, isocitrate dehydrogenase was found to be targeted by lysine acetylation at several sites, some being activating (gain-of-function) an some inactivating (loss-of-function). For fructose-1,6-bisphosphate aldolase (short: aldolase) it was observed that acetylation at several sites differentially affect the binding to F-actin and aldolase enzymatic activity (Venkat et al., 2018; Barbosa Leite et al., 2020). For the enzyme xanthine phosphoribosyltransferase, involved in purine metabolism, a change in stoichiometry of acetylation of 39% was discovered dependent on the presence of CobB. If this acetylation affects enzyme structure and function has not been further explored (Baeza et al., 2014). Often metabolic enzymes are acetylated at several sites, albeit with low overall stoichiometry (Baeza et al., 2014). In that context it should be stressed that for switching off an enzyme function (loss-of-function), high stoichiometries are needed. However, if there are several sites of low stoichiometry, these might have an additive effect and might therefore be of physiological significance. If several enzymes in a metabolic pathway are acetylated each contributing to some extend to modulate enzyme activity, these might sum up to result in a strong overall impact affecting metabolic flux. For glycolysis it was shown that non-enzymatic acetylation resulting in low stoichiometry acetylation affects glycolytic flux (Kulkarni et al., 2017; Li et al., 2019). Moreover, if multi-subunit metabolic enzymes are showing cooperativity in their activity, the acetylation on one subunit might affect the overall activity. These studies revealed that often enzymes that are involved in the generation of utilization of acetyl-CoA are targeted by reversible lysine acetylation, as observed for Acs (Baeza et al., 2014). During metabolism diverse high-reactive acyl-thioesters are produced and consumed. All of these acyl-thioesters can account for non-enzymatic lysine acylation affecting protein structure and function and thereby also metabolic flux (Baldensperger and Glomb, 2021). As an example, 1,3-bisphosphoglycerate formed by glycerinaldehyd-3-phosphate dehydrogenase during glycolysis was shown to act as donor molecule for non-enzymatic lysine 3-phosphoglycerinylation resulting in impairment of their catalytic activity ultimately decreasing glycolytic flux under conditions of high glucose levels (Moellering and Cravatt, 2013; Baldensperger and Glomb, 2021). This suggests a feedback control mechanism for these enzymes *via* lysine ac(et)ylation. If and to which extend these modifications are present in bacteria and if there are enzymes capable to remove these modifications needs to be analyzed systematically in the future.

## Transcription

Lysine acetylation was initially identified on histones regulating RNA synthesis. Today it is known that lysine ac(et)ylation in the histone tails is an important regulator for gene expression affecting chromatin dynamics directly and also indirectly recruitment of chromatin modifying enzymes such as KATs and methyltransferases. While bacteria do not contain histones, they have basic histone-like proteins that fulfill functions in DNA-stabilization, -topology and -organization, transcription, translation and replication. Recent mass-spectrometric data show that the histone-like protein HU in *E. coli* is lysine acetylated implicating functions similar to those described for eukaryotic histones (Weinert et al., 2013a). In *M. tuberculosis* the acetyltransferase Eis was reported to acetylate HU in the C-terminal domain affecting DNA-binding potential (Ghosh et al., 2016). The sirtuin deacetylase Rv1151c in *M. tuberculosis* was able to reverse Hu acetylation. This enables a full acetylation-deacetylation cycle in *M. tuberculosis* HU similar as observed for mammalian histones allowing to reversibly condense and decondense chromatin (Ghosh et al., 2016). HU is involved in formation of a multiprotein complex needed for regulation of the initiation of transcription of the *gal* operon (Geanacopoulos et al., 2001; Kar and Adhya, 2001). The *gal*-operon encodes enzymes for utilization of galactose as carbon source. It was shown that the expression of the *gal*-operon is controlled by the central cAMP-activated transcriptional regulator CAP/CRP (Passner et al., 2000). Binding of CAP/CRP to the gal promotor results in activation of *gal*-operon transcription. CAP/CRP was shown to be involved in regulation of expression of more than 100 genes, many of which are involved in energy metabolism (Martinez-Antonio and Collado-Vides, 2003; Browning et al., 2004; Shimada et al., 2011). Mechanistically, binding of CAP/CRP to the promotor regions of the target genes/operons enables recruitment of DNA-dependent RNA-polymerase (RNAP) to initiate transcription. CAP/CRP was shown to directly be regulated by lysine acetylation (Davis et al., 2018). Performing mutational studies including Lys to Gln/Arg to mimic the acetylated and non-acetylated state acetylation of CAP/CRP at K100 was suggested to impair transcription of class II promotors predominantly due to charge neutralization while the impact on class I promotors was diverse, mostly resulting in transcriptional activation in stationary growth phase but to transcriptional inactivation in exponential growth phase (Davis et al., 2018). Structural modeling suggested that acetylation of CAP/CRP at K100 might interfere with binding to the N-terminal domain of RNAP (Davis et al., 2018). K100-acetylation of CAP/CRP was shown to be driven non-enzymatically by acetyl-phosphate suggesting its accumulation during carbon overflow when intracellular acetyl-phosphate concentrations are high (Schilling et al., 2015; Davis et al., 2018). It is not known if CAP/CRP acetylation can be reverted enzymatically.

Several other transcriptional regulators involved in metabolism, biofilm formation, virulence and cell division were shown to be lysine acetylated. The response regulator and transcriptional regulator RcsB is involved in various processes such as biofilm formation and cell division (Mika and Hengge, 2013; Pannen et al., 2016). It was reported to be enzymatically acetylated at K180 (RcsB AcK180) by *S. enterica* Pat or *E. coli* PatZ. CobB was able to deacetylate RscB AcK180 *in vitro*. K180-acetylation resulted in diminished *flhDC* promotor binding (Thao et al., 2010). Moreover, RcsB was reported to be regulated by non-enzymatic phosphorylation and acetylation mediated by the high energy molecule acetyl-phosphate (Hu et al., 2013). This acetylation was discovered at RscB K154 (RcsB AcK154). Again, applying a mutational analysis RcsB K154Q and K154R were analyzed to assess a potential role of acetylation on its activity. It was shown *in vivo* that acetyl-phosphate driven acetylation of RcsB on K154 results in an impaired transcription from the small RNA promotor *rprA* most likely by interfering with DNA-binding (Hu et al., 2013). Using a genetic approach it was furthermore suggested that RcsB AcK154 is deacetylated by CobB (Hu et al., 2013).

The transcription factor and response regulator PhoP is involved in response to various environmental stress conditions and for bacterial virulence was shown to be regulated by lysine acetylation (Li et al., 2021). Acetylation of *S. typhimurium* PhoP at K88 (PhoP AcK88) and K102 (PhoP AcK102) resulted in impairment of PhoP activity. Borth, K88- and K102-acetylation were mediated by acetyl-phosphate and the acetylation levels decreased strongly upon PhoP activating conditions such as low Mg^2+^ levels, acid stress and phagocytosis of *S. typhimurium* by macrophages (Ren et al., 2019; Li et al., 2021). Moreover, *S. typhimurium* expressing a PhoP K88Q acetylation mimetic mutant showed attenuated virulence in mice infectivity suggesting that approaching PhoP K88 acetylation status might constitute an interesting therapeutic intervention to treat *S. typhimurium* infection (Li et al., 2021). Mechanistically, PhoP K88-acetylation interferes with PhoP dimerization and DNA-binding (Li et al., 2021). Another study showed that acetylation of PhoP at K201 is mediated by Pat and is removed by CobB as shown indirect using a CobB s. typimurium deletion strain (Ren et al., 2016). PhoP K201 is directly located in the DNA-binding region and K201-acetylation impaired DNA-binding with direct consequences on the *S. typhimurium* stress response and pathogenesis (Ren et al., 2016).

Finally, the *E. coli* transcriptional regulator RutR was identified as being lysine acetylated on two lysines, K52 and K62, within the helix-turn-helix motif essential for DNA-binding (Tu et al., 2015). RutR is a transcription factor important for pyrimidine metabolism (Tu et al., 2015). It was suggested that RutR acetylation can be reverted by a putative novel deacetylase in *E. coli* called YcgC (original name: DhaM). YcgC was originally characterized as component of the dihydroxyacetone kinase complex catalyzing the phosphoryl group transfer toward dihydroxyacetone using phosphoenolpyruvate as a donor molecule. YcgC (DhaM) is the phosphoryl donor in this process (Gutknecht et al., 2001; Shi et al., 2011). However, our laboratory showed that YcgC is no deacetylase (Kremer et al., 2018). Future studies are needed to show mechanistically how lysine acetylation affects RutR function.

These are some important examples how post-translational lysine acetylation of histone-like proteins and of transcription factors is used to directly translate the cellular metabolic state into altered and adjusted gene expression levels. This allows the cells to quickly adjust the cells to altered environmental conditions.

## Translation

Another important cellular process, in which acetylation plays an important role in bacteria is translation. Here, we explained earlier already that the enzymes RimI, RimJ and RimL are active as N-terminal acetyltransferases acetylating the ribosomal proteins S18, L5, and S12, respectively (Yoshikawa et al., 1987; Tanaka et al., 1989). It is still not completely understood, what role this N-terminal acetylation has *in vivo* but it might affect translational efficiency.

Several important players in translation were shown to be targeted by lysine acetylation, amongst them several aminoacyl-tRNA-synthetases, translational elongation/release/initiation factors as shown by mass-spectrometry (Weinert et al., 2013a; Zhang et al., 2013; Baeza et al., 2014). As an example, the GTP-binding protein and elongation factor EF-Tu (elongation factor thermal unstable) brings the aminoacyl-tRNAs, i.e., the tRNAs that are charged by aminoacyl-tRNA synthetases with the cognate amino acid, to the ribosomal A-site. Upon GTP-hydrolysis to GDP and P_i_, which is stimulated by binding to the ribosome, EF-Tu releases the aminoacyl-tRNA at the A-site. EF-Tu is subsequently reloaded with GTP by the guanine-nucleotide exchange factor EF-Ts (elongation factor temperature sensitive). Several mass-spectrometric screens identified lysine acetylation sites in *E. coli* EF-Tu, which might regulate EF-Tu aminoacyl-tRNA-binding, GTP loading or nucleotide exchange (Zhang et al., 2009; Weinert et al., 2013a). Moreover, *B. subtilis* EF-Tu (TufA) was shown to be lysine acetylated and lysine succinylated in domain-3 with a negative effect on translation and growth (Suzuki et al., 2019). The role of acetylation on function of *E. coli* alanyl-tRNA-synthetase and tyrosyl-tRNA synthetase was studied by applying the genetic code expansion concept. For both, acetylation was shown to reduce enzymatic activity (Venkat et al., 2017a; Umehara et al., 2018). These studies allowed to unravel the real impact of lysine acetylation on aminoacyl-tRNA synthetase function rather than performing mutational studies (Venkat et al., 2017a; Umehara et al., 2018). For tyrosyl-tRNA synthetase acetylation at three positions, K85, K235 and K238, resulted in a reduction of catalytic activity. None of the sites was enzymatically acetylated but chemically by treatment of the recombinantly expressed and purified enzyme by acetyl-CoA and acetyl-phosphate. CobB was able to remove acetylation from these positions (Venkat et al., 2017a).

Finally, several bacteria encode toxin-antitoxin systems that allow the bacteria to induce a persister state by downregulation of essential cellular processes such as translation. As an example, *S. enterica* was reported to induce a persister state through activation of the TacT toxin-TacA antitoxin system. TacA forms a complex with TacT. TacT is a GNAT acetyltransferase that was shown to acetylate aminoacyl-tRNAs at free amino groups which results in blocking the formation of peptide bonds by the ribosome (Jurenas et al., 2017; Yeo, 2018). Moreover, TacT was shown to lysine acetylate TacA within the TacT•TacA complex which stimulates the TacT acetyltransferase activity. Further toxin-antitoxin systems with acetyltransferase activity were described in *E. coli* (Hall et al., 2017; Goormaghtigh et al., 2018).

The toxin AtaT is a GNAT acetyltransferase that acetylates the initiator methionine-tRNA, resulting in arrest of translation initiation by preventing the interaction with the initiation factor 2 (IF2) (Jurenas et al., 2017). While toxin-antitoxin systems were known to be involved in maintenance of plasmids in bacterial populations, recently, toxin-antitoxin gene pairs were identified as part of a genetic mobile element, the transposon Tn3, suggesting a role in stabilizing the transposon and enabling a stable invasion during transposition (Kamruzzaman and Iredell, 2019; Lima-Mendez et al., 2020). Several excellent reviews are published on the different bacterial toxin-antitoxin including a classification, which is beyond the scope of this review (Hall et al., 2017; Jurenas et al., 2017; Goormaghtigh et al., 2018; Yeo, 2018).

## Conclusion and Perspectives

Lysine ac(et)ylation is an important post-translational modification in all domains of life. Research performed on lysine ac(et)ylation in bacteria suggests that lysine ac(et)ylation is important for various essential cellular processes. It acts as a sensor for the metabolic state and translates this directly into altered protein functionalities to adjust cellular processes to altering conditions. Of particular interest are mass-spectrometry-based studies that performed determination of kinetics and stoichiometry of lysine acetylation (Baeza et al., 2014; Weinert et al., 2017, 2018; Baeza et al., 2020). These screens revealed that the major fraction (~80%) of acetylated proteins occur at stoichiometry of 0–10% in *E. coli*. However, there are 16% of acetylations that accumulate to stoichiometries between 10 and 20% and approximately 4% of all sites accumulate to stoichiometries of >20% (Baeza et al., 2014; Weinert et al., 2017). Importantly, the stoichiometries range from <1 to 98% suggesting that there are several sites, which are of great physiological impact under the conditions analyzed (Baeza et al., 2014). If judging the overall physiological significance of lysine ac(et)ylation it is important to consider that the mass-spectrometric studies are only representing the actual conditions under which these studies are performed. Different conditions and interventions might uncover different physiologically important acetylation sites. However, from what is known about lysine ac(et)ylation it is a highly dynamic modification often occurring on time dimensions of minutes to hours which strongly depend on the metabolic state of the organisms (Baeza et al., 2020). Sites that persist or accumulate on longer time dimensions might often represent non-enzymatic ac(et)ylations at lower stoichiometry that cells have to cope with to avoid a continuous sneaking of protein functions with age. To this end, it is likely that many more proteins will be discovered for which lysine ac(et)ylation represents an important post-translational modification to regulate structure and function. This is one of the biggest challenges in the research filed to uncover those ac(et)ylation sites of the thousands identified that of real physiological importance. This should be judged by different criteria: (1) the ac(et)ylation should affect protein function, (2) the ac(et)ylation should be regulated enzymatically and/or non-enzymatically, (3) the acetylation should accumulate to stoichiometries *in vivo* suitable and appropriate for the physiological function. As stated above, a high stoichiometry is not an essential requirement for a physiological importance. However, for a loss-of-function effect a high stoichiometry should be reached, while for a gain-of-function, a acetylation does not need to reach high stoichiometries and a sub-pool of modified protein molecules might be sufficient for a physiological outcome. To identify those sites that are of physiological significance, further mass-spectrometry based studies should focus on interventions that allows to detect physiologically relevant sites such as conditions under which the expression or subcellular localization of a acetyltransferase or deacetylase/sirtuin in affected. Systemic studies that are performed under conditions of metabolic fuel switching that are often conducted for days in cells or even weeks/months in mice will mostly result in systemic acetylation at low occupancy. In that context, finding many sites by mass-spectrometry is not a criterion of quality in the sense that those sites are of physiological importance. It is far better to find a few physiologically important acetylation sites than finding thousands that are background noise due to chemical acetylation. Moreover, studies with mammalian cells showed that lysine acetylation occurs in diverse kinetic profiles following an intervention such as serum stimulation. Some accumulate to high stoichiometries rapidly within minutes to hours such as sites on proteins affecting translation (Weinert et al., 2018; Baeza et al., 2020). These sites are most likely sites which are enzymatically catalyzed and are therefore highly interesting. Particularly for bacteria, which have to deal with quickly altering environmental conditions, those mass-spectrometry workflows including information on the kinetics and the stoichiometry will be highly important in the future.

Moreover, to study the real consequences of lysine-ac(et)ylation for protein the genetic code expansion concept (GCEC) should find a broader use. As lysine ac(et)ylation uses diverse mechanisms to regulate protein function, each ac(et)ylation has to be analyzed on a protein and even on a site-specific basis. We observed in several cases that using Lys to Gln and Lys to Arg mutations can be misleading resulting in misinterpretation of the influence of lysine acetylation. The *Methanosarcina* AcKRS/PylT system can be used in all model organisms *in vitro* and even *in vivo*. However, while some groups used the GCEC to study the role of lysine acetylation *in vitro* using recombinantly expressed proteins almost no study applied the technology for studies in bacteria *in vivo*. Other important questions in the field are how post-translational lysine ac(et)ylation interferes and crosstalks with other post-translational modifications. Moreover, it will be interesting to elucidate how other lysine acylations than acetyl lysine regulate protein function. To this end, the development of further synthetic biological systems than allow the efficient incorporation of other lysine acylations site-specifically in proteins, such as lysine malonylation or lysine succinylation, will be important. Other synthetic biological tools such as genetically encoding photoactivatable crosslinkers combined with mass-spectrometry can be applied to enable the identifications of further substrates and interaction partners of acetyltransferases, classical deacetylases and sirtuins. This is particularly powerful as these systems allow to stabilize otherwise very transient interactions. This should also be applied to CE-clan related DUBs/AcTs from pathogenic Gram-negative bacteria for the identification of novel substrates and interaction partners and for the characterization of the physiological processes. Finally, future studies will show if there are further acetyltransferases or deacetylases or even ac(et)yl lysine reader domains such as bromodomains, YEATS domains or structurally unrelated domains encoded by bacteria that allow the recruitment of proteins *via* lysine ac(et)ylation or even the formation of signal transduction cascades. As a summary, many open questions exist in the research field that need to be addressed in the future.

## Author Contributions

ML made the concept and design of the review, wrote the manuscript, prepared figures, revised the manuscript, and submitted the manuscript.

## Conflict of Interest

The author declares that the research was conducted in the absence of any commercial or financial relationships that could be construed as a potential conflict of interest.

## Publisher’s Note

All claims expressed in this article are solely those of the authors and do not necessarily represent those of their affiliated organizations, or those of the publisher, the editors and the reviewers. Any product that may be evaluated in this article, or claim that may be made by its manufacturer, is not guaranteed or endorsed by the publisher.

## Supplementary Material

Supplementary Figure 1 Structural characterization of bacterial type III GNATs. **(A)** The N-terminal cAMP-binding domain in *M. tuberculosis Mt*Pat (Rv0998) (PDB: 4AVC) is structurally highly similar to cAMP-binding domains of *Bos taurus* protein kinase A (PKA) (PDB: 1RGS), *E. coli* CAP/CRP (PDB: 1O3T), and *M. musculus* EPAC2 (PDB: 3CF6). The cAMP-binding domain in all of these proteins is involved in regulation of their activities. Binding of cAMP to *Mt*Pat results in allosteric activation of the C-terminal GNAT domain. **(B)** *Myxococcus xanthus Mx*PAT (Mxan_3215) shows structural similarity to the N-terminal domain of human epimerase SDR39U1 (PDB: 4B4O) as shown using Phyre2. The NADP^+^-binding Rossmann-fold domain in *Mx*PAT is located N-terminally to the C-terminal GNAT domain. Binding of NADP^+^ results in inhibition of the GNAT catalytic activity. **(C)** *Bacillus subtilis* encodes for a GNAT (*Bs*AcuA) and a classical deacetylase (*Bs*AcuC) as part of the *acuABC* operon. The role of the *Bs*AcuB encoded by *acuB* is not understood. Structural modeling with Phyre2 reveals similarities to a tandem CBS (cystathionine-beta-synthase) domain and a C-terminal ACT domain. This opens the hypothesis that *Bs*AcuB might regulate *Bs*AcuA GNAT and or *Bs*AcuC activity *in trans* rather than *in cis*. The structural models were created with Phyre2 (Kelley et al., 2015) [the figure was generated with PyMOL v.2.3.4 (Schrödinger LLC, 2000)].

Supplementary Figure 2 Chemical structures of molecules discussed in this review. **(A)** Molecules involved in deac(et)ylation catalyzed by sirtuin deac(et)ylases. NAD^+^ is the stoichiometric co-substrate for sirtuins, nicotinamide is a non-competitive inhibitor of sirtuins, 2′-*O*-acetyl-ADP-ribose is a product of the sirtuin catalyzed reaction and can be converted to ADP-ribose by macrodomains, such as YmdB from *E. coli* and MacroD from *S. aureus*. Both, 2′-*O*-acetyl-ADP-ribose and ADP-ribose might act as signaling molecules in bacteria. c-di-GMP was recently shown to act as a modulator of the activity of the long isoform of CobB. **(B)** Molecules important for GNAT catalysis and regulation. Acetyl-CoA is the acetyl-group donor molecule for GNATs. Other acyl-CoA molecules might also be used by bacterial GNATs. This needs additional research. NADP^+^ and 3′,5′-cyclic AMP (cAMP) were shown to be allosteric regulators for GNAT activity in bacteria. **(C)** Potent inhibitors for classical deacetylases in bacteria. Mammalian KDACs were shown to be selectively and potently inhibited by the hydroxamates suberoylanilide hydroxamic acid (SAHA; vorinostat) and trichostatin A (TSA). The mode of action involves the chelation of the catalytic Zn^2+^-ion. SAHA and TSA were shown to be also potent in inhibiting bacterial classical deacetylases. Further studies are needed for development of enzyme specific inhibitors. **(D)** Isotopically labeled molecules applied for chemical labeling in mass-spectrometry workflows to uncover systemic acetylation stoichiometry, including acetic anhydride-d_6_, acetic anhydride-^13^C,d_6_, and *N*-acetoxy-d_3_-succinimide.
