# Supplementary material for: Post-translational Lysine Ac(et)ylation in Bacteria: A Biochemical, Structural, and Synthetic Biological Perspective
Source: Front Microbiol. 2021 Oct 11;12:757179. doi: 10.3389/fmicb.2021.757179 (PMC8556138; doi:10.3389/fmicb.2021.757179)
Supplement: Supplementary Figure 1 — Structural characterization of bacterial type III GNATs. (A) The N-terminal cAMP-binding domain in M. tuberculosis MtPat (Rv0998) (PDB: 4AVC) is structurally highly similar to cAMP-binding domains of Bos taurus protein kinase A (PKA) (PDB: 1RGS), E. coli CAP/CRP (PDB: 1O3T), and M. musculus EPAC2 (PDB: 3CF6). The cAMP-binding domain in all of these proteins is involved in regulation of their activities. Binding of cAMP to MtPat results in allosteric activation of the C-terminal GNAT domain. (B) Myxococcus xanthus MxPAT (Mxan_3215) shows structural similarity to the N-terminal domain of human epimerase SDR39U1 (PDB: 4B4O) as shown using Phyre2. The NADP+-binding Rossmann-fold domain in MxPAT is located N-terminally to the C-terminal GNAT domain. Binding of NADP+ results in inhibition of the GNAT catalytic activity. (C) Bacillus subtilis encodes for a GNAT (BsAcuA) and a classical deacetylase (BsAcuC) as part of the acuABC operon. The role of the BsAcuB encoded by acuB is not understood. Structural modeling with Phyre2 reveals similarities to a tandem CBS (cystathionine-beta-synthase) domain and a C-terminal ACT domain. This opens the hypothesis that BsAcuB might regulate BsAcuA GNAT and or BsAcuC activity in trans rather than in cis. The structural models were created with Phyre2 (Kelley et al., 2015) [the figure was generated with PyMOL v.2.3.4 (Schrödinger LLC, 2000)]. [file Presentation_1.pdf]

Supplementary Material

Post-translational lysine ac(et)ylation in bacteria: a structural, synthetic biological and functional perspective.

Michael Lammers*

Institute for Biochemistry, [1]Synthetic and Structural Biochemistry, University of Greifswald, Greifswald, Germany

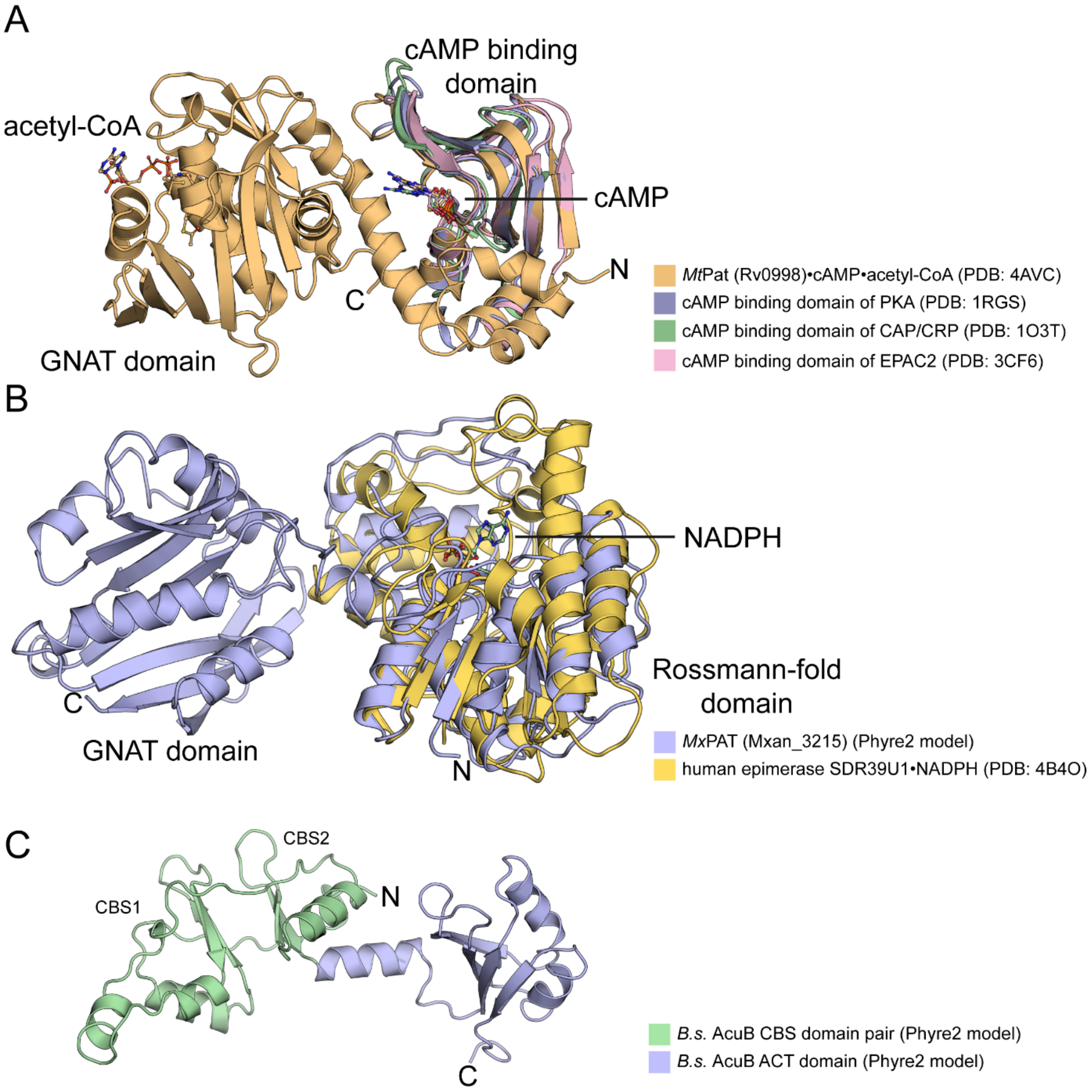

Supp. Figure 1: Structural characterization of bacterial type III GNATs.

(A) The N-terminal cAMP binding domain in *M. tuberculosis* MtPat (Rv0998) (PDB: 4AVC) is structurally highly similar to cAMP binding domains of *Bos taurus* protein kinase A (PKA)

(PDB: 1RGS), *E. coli* CAP/CRP (PDB: 1O3T) and *M. musculus* EPAC2 (PDB: 3CF6). The cAMP binding domain in all of these proteins is involved in regulation of their activities. Binding of cAMP to *Mt*Pat results in allosteric activation of the C-terminal GNAT domain.

(B) *Myxococcus xanthus Mx*PAT (Mxan_3215) shows structural similarity to the N-terminal domain of human epimerase SDR39U1 (PDB: 4B4O) as shown using Phyre2. The $NADP^+$-binding Rossmann-fold domain in *Mx*PAT is located N-terminally to the C-terminal GNAT domain. Binding of $NADP^+$ results in inhibition of the GNAT catalytic activity.

(C) *Bacillus subtilis* encodes for a GNAT (*Bs*AcuA) and a classical deacetylase (*Bs*AcuC) as part of the a*cuABC* operon. The role of the *Bs*AcuB encoded by *acuB* is not understood. Structural modeling with Phyre2 reveals similarities to a tandem CBS (cystathionine-beta-synthase) domain and a C-terminal ACT domain. This opens the hypothesis that *Bs*AcuB might regulate *Bs*AcuA GNAT and or *Bs*AcuC activity *in trans* rather than *in cis*. The structural models were created with Phyre2 (Kelley et al., 2015).

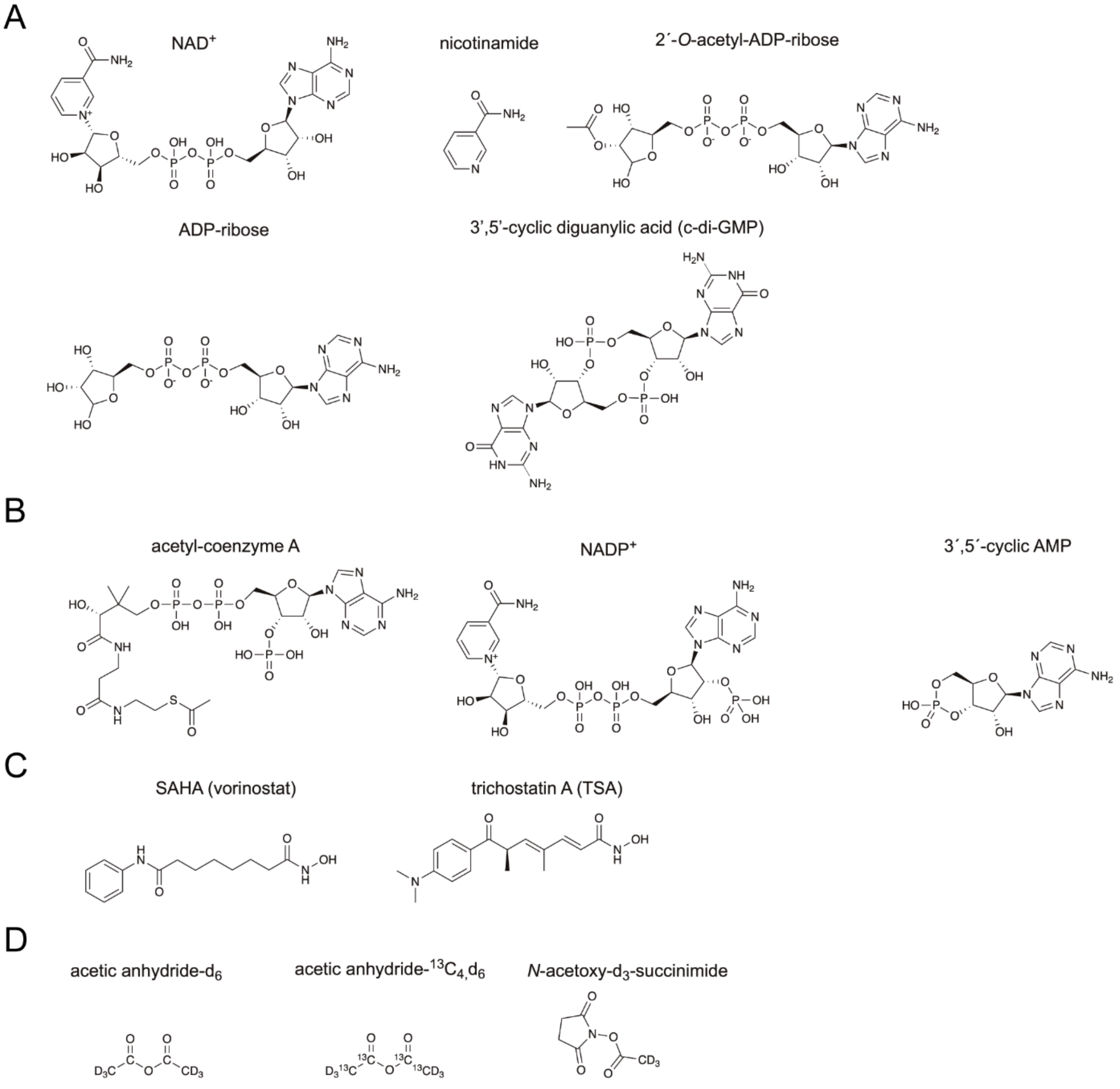

Supp. Figure 2: Chemical structures of molecules discussed in this review.

(A) Molecules involved in deac(et)ylation catalyzed by sirtuin deac(et)ylases. NAD^+ is the stoichiometric co-substrate for sirtuins, nicotinamide is a non-competitive inhibitor of sirtuins, 2’-*O*-acetyl-ADP-ribose is a product of the sirtuin catalyzed reaction and can be converted to ADP-ribose by macrodomains, such as YmdB from *E. coli* and MacroD from *S. aureus*. Both, 2’-*O*-acetyl-ADP-ribose and ADP-ribose might act as signaling molecules in bacteria. c-di-GMP was recently shown to act as a modulator of the activity of the long isoform of CobB.

(B) Molecules important for GNAT catalysis and regulation. Acetyl-CoA is the acetyl-group donor molecule for GNATs. Other acyl-CoA molecules might also be used by bacterial

GNATs. This needs additional research. $NADP^+$ and 3',5'-cyclic AMP (cAMP) were shown to be allosteric regulators for GNAT activity in bacteria.

(C) Potent inhibitors for classical deacetylases in bacteria. Mammalian KDACs were shown to be selectively and potently inhibited by the hydroxamates suberoyl anilide hydroxamic acid (SAHA; vorinostat) and trichostatin A (TSA). The mode of action involves the chelation of the catalytic Zn^{2+}-ion. SAHA and TSA were shown to be also potent in inhibiting bacterial classical deacetylases. Further studies are needed for development of enzyme specific inhibitors.

(D) Isotopically labeled molecules applied for chemical labeling in mass spectrometry workflows to uncover systemic acetylation stoichiometry, including acetic anhydride-d_6, acetic anhydride-$^{13}C,d_6$ and *N*-acetoxy-d_3-succinimide.

Reference:

Kelley, L.A., Mezulis, S., Yates, C.M., Wass, M.N., and Sternberg, M.J. (2015). The Phyre2 web portal for protein modeling, prediction and analysis. *Nat Protoc* 10(6)**,** 845-858. doi: 10.1038/nprot.2015.053.
